# An atlas of plant selenium metabolism

**DOI:** 10.1111/nph.71087

**Published:** 2026-03-16

**Authors:** Jeroen van der Woude, Mark G. M. Aarts, Michela Schiavon, Antony van der Ent

**Affiliations:** ^1^ Laboratory of Genetics Wageningen University and Research Droevendaalsesteeg 1 6708 PB Wageningen the Netherlands; ^2^ Department of Agricultural, Forest and Food Sciences (DISAFA) University of Turin Grugliasco Turin 10095 Italy

**Keywords:** abiotic stress, Arabidopsis, elementome, hyperaccumulator, metabolic pathway, selenium, stress tolerance, sulfur

## Abstract

Selenium (Se) is not only a rare and toxic element but also an essential micronutrient for humans and animals that is often in short supply. Terrestrial plants do not require Se, but it can have growth‐promoting or negative effects, depending on the exposure level. In this Tansley review, we draw up a comprehensive metabolic map of the known plant Se metabolism and the sulfur (S) metabolism which it largely mirrors. We compile the current knowledge of plant selenometabolites, enzymes that handle this element and genes that affect Se uptake and tolerance. Large literature datasets are used to place Se in the overall elemental composition of land plants and to compare the transcriptome of Se‐exposed *Arabidopsis thaliana* to the S deficiency response. Focus is placed on Se hyperaccumulator species, which can attain extremely high concentrations of Se in their tissues. We identify seven broad tolerance strategies to prevent Se toxicity, which itself has two faces: the oxidative stress of inorganic Se and the S‐mimicking properties of organic Se compounds. This review, supplementary datasets and figures are intended as a comprehensive resource to guide plant Se research and help improve crop Se levels for a healthy future.

**Table:** 

	Contents	
	Summary	2041
I.	Selenium biochemistry: a false mirror of sulfur biochemistry	2041
II.	Selenium in plants: toxic or beneficial?	2042
III.	Selenium in agricultural and ecological settings	2047
IV.	Hypertolerance and accumulation: the arsenal of Se hyperaccumulators	2049
V.	Digging deeper: open questions in plant Se physiology	2053
VI.	Conclusions and outlook	2055
	Acknowledgements	2055
	References	2055

## I. Selenium biochemistry: a false mirror of sulfur biochemistry

### 1. Selenium is a rare and reactive sulfur‐analog

Selenium (Se) and sulfur (S) share a similar (bio)chemistry, but their geochemical abundances differ dramatically: S is *c*. 300‐fold more common (2920 μg S g^−1^) in the Earth's crust compared with Se (9.6 μg Se g^−1^) (Morgan & Anders, 1980). There are also chemical differences between Se and S, particularly in redox behavior, bond energies and reaction rates (Wessjohann *et al*., 2007; Reich & Hondal, 2016). Both these similarities and differences contribute to Se toxicity in biological systems. Inorganic Se can generate oxidative stress, while organic Se species may inadvertently replace S in biomolecules, leading to dysfunctional proteins and S metabolites (Van Hoewyk, 2013). Despite its high toxicity, Se has been used by biological life since the Last Universal Common Ancestor (LUCA) *c*. 4000 million years ago (Weiss *et al*., 2016). Today, biological Se utilization is mainly associated with redox biochemistry, where Se can offer a catalytic advantage over S by up to 300‐fold (Wessjohann *et al*., 2007) or increase the oxygen tolerance of certain enzymes (Reich & Hondal, 2016). Meanwhile, the specific incorporation of the so‐called 21^st^ amino acid selenocysteine (Sec) in proteins is a costly metabolic process, which requires the recoding of a stop‐codon (UGA) into a Sec‐codon during ribosomal translation (Reich & Hondal, 2016; Mariotti *et al*., 2019).

**Fig. 1:**
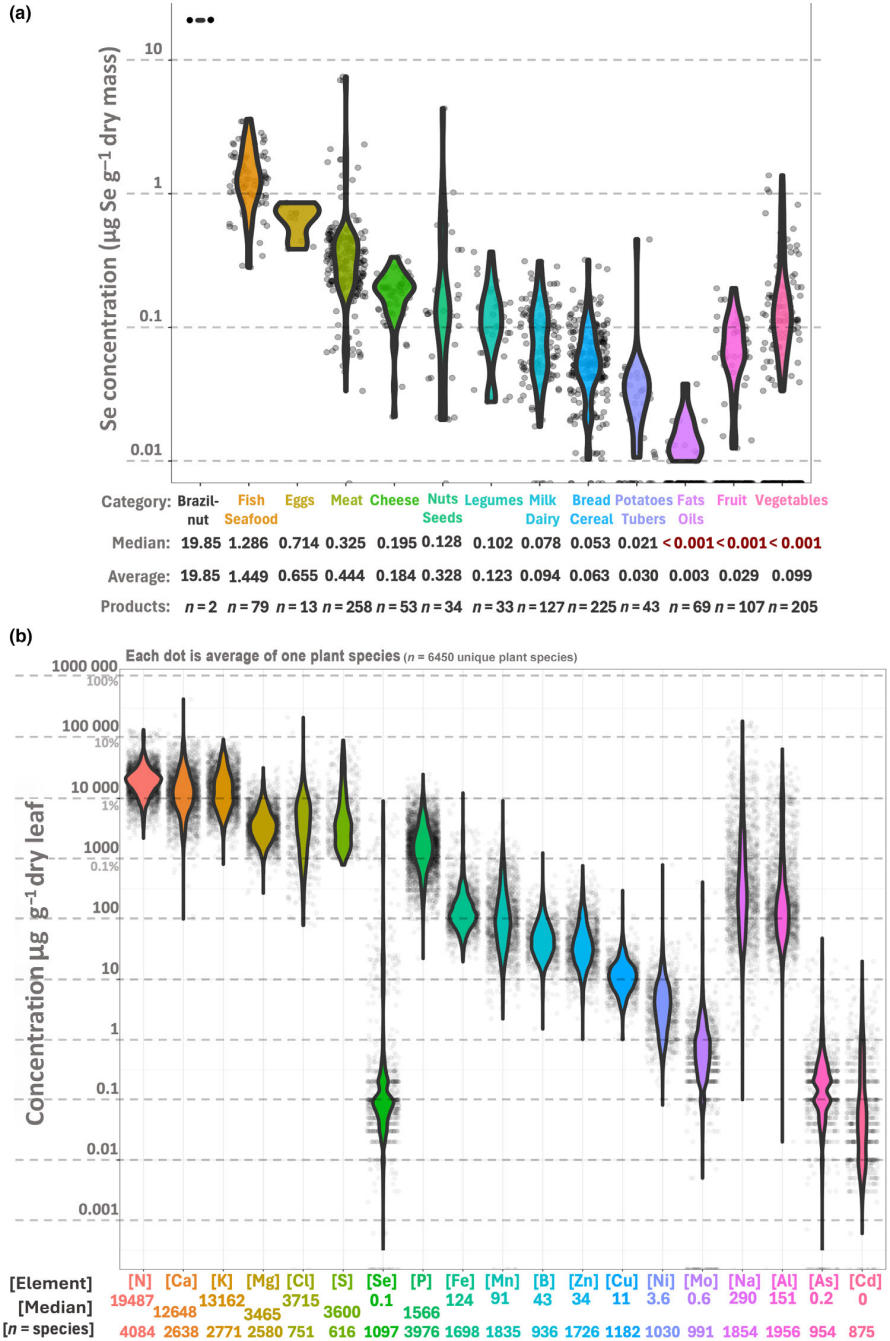
The amount of Se in different food categories (a) and the amount of Se and 18 other chemical elements in 6450 plant species are quantified (b). All the datapoints are plotted as dots overlayed under the violin plots. The Se content of Dutch food products is plotted in (a), based on the ‘Nederlands Voedingsstoffenbestand’ (NEVO) Online database (NEVO online v.2023/8.0, Rijksinstituut voor Volksgezondheid en Milieu (RIVM), Bilthoven). The food products are grouped together and ordered according to decreasing median Se value. Brazil nuts are placed in a separate category, since these products contain extreme levels of Se. In (b), the typical elementome of angiosperms is depicted, with each dot representing an average of a plant species. As input data, the elemental composition data of 6450 plant species from 39 publications is used (see Supporting Information Table S6 for the sources). The elements are ordered based on decreasing average values, except for Se which is placed between S and P for comparison, and since Se hyperaccumulators can reach levels in this concentration range. Included in the elemental fingerprint are all 15 plant nutrients, as well as sodium and aluminum due to their ubiquitous presence. Arsenic and cadmium are also included, to emphasize that in most plants the Se level is similar to that of these two highly toxic chemical elements. The source data are available in Table S11 for (a) and Tables S5 and S6 for (b). Al, aluminum; As, arsenic; B, boron; Ca, calcium; Cd, cadmium; Cl, chlorine; Cu, copper; Fe, iron; K, potassium; Mg, magnesium; Mn, manganese; Mo, molybdenum; N, nitrogen; Na, sodium; Ni, nickel; P, phosphorous; S, sulfur; Se, selenium; Zn, zinc.

Likely due to the limited Se availability in certain ecological niches and across geological time, the metabolic essentiality of Se has been lost during evolution of most fungi and insects as well as vascular plants, although small amounts can be beneficial to the latter (Novoselov *et al*., 2002; Mesquita *et al*., 2015; Mariotti *et al*., 2019). By contrast, mammals never lost the requirement for Se, which was officially recognized as a micronutrient after Schwarz & Foltz (1957), demonstrated its essential role in promoting healthy growth in rats. Before this discovery, Se was only considered a poisonous element, known to cause selenosis when consumed in excess, manifested as hair and nail loss, nervous disorders, alkali disease or blind staggers in grazing animals (Raisbeck, 2000). Half a century later, the scientific view on Se shifted substantially. Insufficient intake of Se is now linked to various pathological conditions in humans, including certain cancers, coronary heart disease/Keshan disease, white muscle disease, Parkinson's disease, cognitive decline and low fertility (Rayman, 2012). Because of its contrasting roles, Se is referred to as an ‘essential poison’. While Se toxicity (daily intake above *c*. 400 μg Se) is endemic to specific regions with extraordinary high soil Se levels (Knott & McCray, 1959; Yang *et al*., 1983), Se deficiency (daily intake below *c*. 55 μg Se) is estimated to affect up to 1 billion people world‐wide and this number is expected to increase as a result of climate change (Jones *et al*., 2017). This is reflected in the low concentration of Se in most non‐seafood products from the Netherlands (Fig. 1a) and the low Se content of most plants (Fig. 1b). Developing a better understanding of plant Se metabolism can help combat Se deficiencies through the development of appropriate Se fertilization techniques (biofortification), breeding of Se‐enriched crops (genetic biofortification) or mitigate (anthropogenic) Se pollution via phytoremediation. The main focus of this review is therefore on the fundamental processes of the Se metabolism in plants, with a minor focus on applied science.

### 2. The plant Se metabolism: compiling the current state of knowledge

**Fig. 2:**
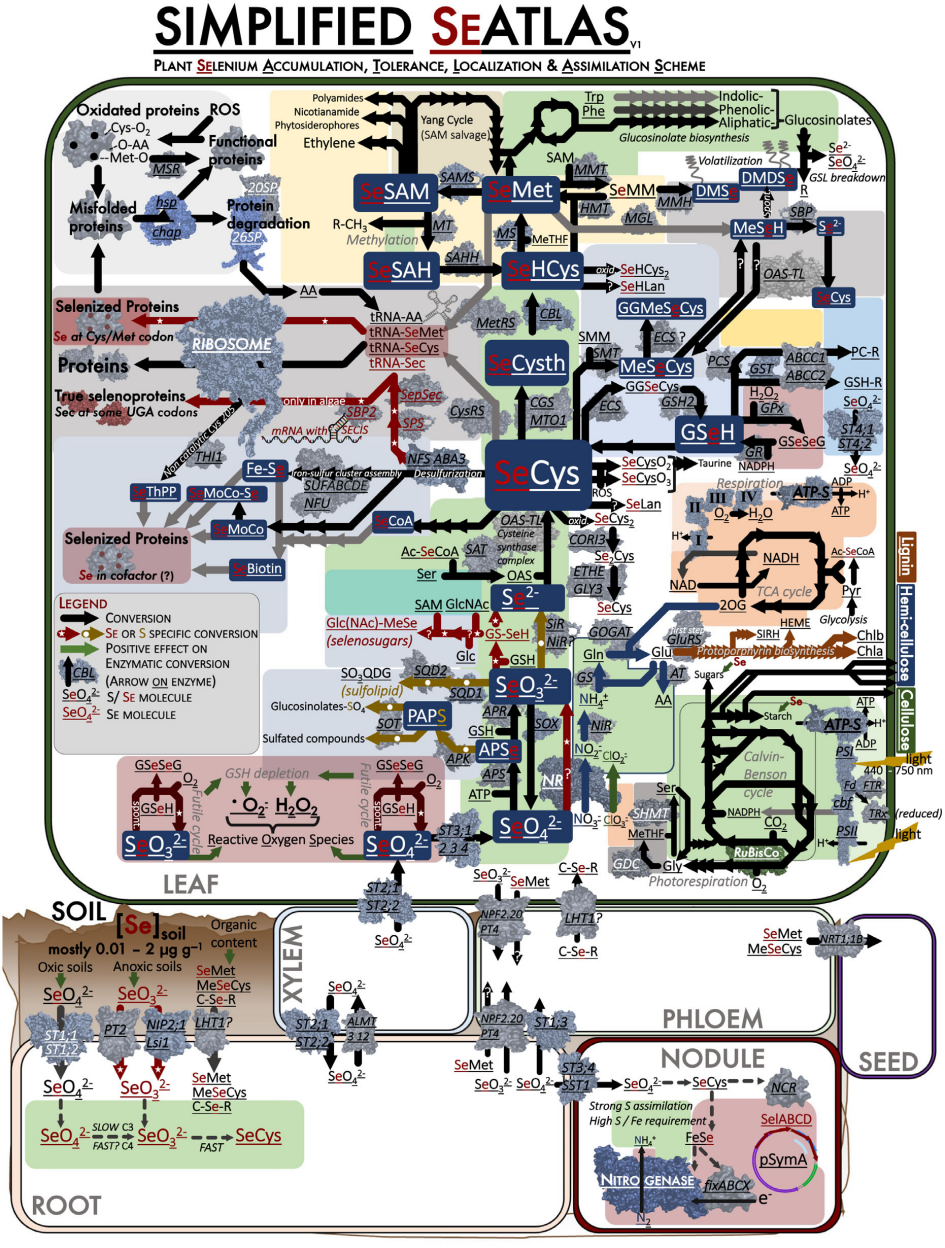
Simplified plant selenium accumulation, tolerance, localization & assimilation scheme (Plant SeATLAS) depicting the metabolic network of S and Se in plants. The full version is found as Supporting Information Fig. S1. This scheme provides a comprehensive representation of selenium metabolism based on studies of plant selenium (Se) metabolism, studies of the plant sulfur (S) metabolism, Se metabolism in other kingdoms of life (mammalian and microbial) and especially the work done on Se‐hyperaccumulator plants. The red color is indicative of the S‐Se analogy, with the red part showing the Se analog of the S compound, red arrows indicating Se‐specific (e.g. nonsulfur) conversions and molecular processes, and yellow arrows indicating S processes so far not known to be mirrored by Se. When the red parts are ignored, this can be used as a plant sulfur metabolic chart. Mechanisms of Se tolerance and accumulation are also shown, along with the most important carbon‐ and nitrogen fluxes in the plant seeing that these are vital in the broader metabolism. A simplified representation of nodule metabolism is also included since various Se‐accumulators are Fabaceae, and nodulation affects both Se and S metabolism. Protein structures indicated with lighter blue represent the actual 3D‐structure of the protein, while dull‐gray colored protein structures are simple placeholders (e.g. most transporters are represented using the Nodulin 26‐like Intrinsic Protein (NIP2;1, an aquaporin) structure). Alphabetic legend to abbreviations: ‘?’, process uncertain; ‘•O2 ‘, superoxide; ‘20SP’, 20S proteasome; ‘26SP’, 26S proteasome; ‘2OG’, 2‐oxoglutarate; ‘AA’, amino acids; ‘ABCC1 2 ‘, multidrug resistance‐associated protein 1; ‘Ac‐SeCoA’, Acetyl‐(seleno)coenzyme A; ‘ALMT 3 12’, aluminum‐activated malate transporter 3; ‐12; ‘APK’, APS kinase; ‘APR’, 5′adenylylphosphosulfate reductase; ‘APS’, 3’‐phosphoadenosine 5’‐phosphosulfate synthase; ‘APSe’, Adenosine 5’‐phosphosulfate/selenate; ‘AT’, aminotransferase; ‘ATP’, adenosinetriphosphate; ‘ATP‐S’, ATP‐synthase; ‘C‐Se‐R’, organic Se compound; ‘C3 / C4’, Plants with C3 or C4 photosynthesis; ‘cbf’, cytochrome b6/f complex; ‘CBL’, cystathionine beta‐lyase; ‘CGS‐MTO1’, cystathionine gamma synthase/methionine overproducing 1; ‘chap’, chaperones; ‘Chla / Chlb’, chlorophyll A / B;  ‘ClO_2_’, Chlorite; ‘ClO_3_’, chlorate; ‘CO_2_’, carbon dioxide; ‘CORI3’, L‐cystine beta‐lyase; ‘Cys’, Cysteine; ‘CysRS’, cysteinyl‐tRNA synthetase; ‘DMDSe’, dimethyldiselenide; ‘DMSe’, dimethylselenide; ‘ECS’, glutamate‐‐cysteine ligase; ‘ETHE‐GLY3’, sulfur dioxygenase; ‘Fd’, ferredoxin; ‘Fe‐Se’, iron–sulfur (iron‐selenium) cluster; ‘fixABCX’, electron transfer flavoproteins; ‘FTR’, ferredoxin/thioredoxin reductase; ‘GDC’, glycine decarboxylase; ‘GGMeSeCys’, gamma‐glutamylmethyl(seleno)cysteine; ‘GGSeCys’, gamma‐glutamyl(seleno)cysteine; ‘Glc’, glucose; ‘GlcNAc’, N‐Acetyl‐D‐glucosamine; ‘Gln’, glutamine; ‘Glu’, glutamate; ‘GluRS’, glutamyl‐tRNA synthetase; ‘Gly’, glycine; ‘GOGAT’, glutamate synthase; ‘GPX’, glutathione peroxidase; ‘GR’, glutathione reductase; ‘GS’, glutamine synthase; ‘GS‐SeH’, Glutathioselenol; ‘GSH/GSeH’, (seleno)glutahione; reduced; ‘GSeSeG’, (seleno)glutathione; oxidated; ‘GSH‐R’, glutathione‐conjugate; ‘GSH2’, glutathione synthase; ‘GST’, glutathione‐S‐transferase; ‘H^+^’, proton; ‘H_2_O’, water; ‘H_2_O_2_’, hydroperoxide; ‘HMT’, homocysteine S‐methyltransferase; ‘hsp’, heat‐shock proteins; ‘I’, complex I NADH dehydrogenase; ‘II’, complex II succinate dehydrogenase; ‘III’, complex III cytochrome bc1 complex; ‘IV’, complex IV cytochrome c oxidase; ‘LHT1’, lysine histidine transporter 1; ‘Lsi1’, Silicon influx transporter; ‘MeSeH’, Methanethiol (methaneselenol); ‘Met’, L‐methionine; ‘MeTHF’, 5‐Methyltetrahydropteroyltri‐L‐glutamate; ‘MetRS’, methionyl‐tRNA synthetase; ‘MGL’, methionine gamma‐lyase; ‘MMH’, methylmethionine hydrolase; ‘MMT’, methionine S‐methyltransferase; ‘mRNA’, messenger RNA; ‘MS’, methionine synthase; ‘MSR’, peptidemethionine sulfoxide reductase; ‘MT’, methyl transferase; ‘NAD/NADH’, nicotinamide adenine dinucleotide; oxidated/reduced; ‘NADP/NADPH’, nicotinamide adenine dinucleotide phosphate; oxidated/ reduced; ‘NCR’, nodule‐specific cysteine‐rich peptide; ‘NFS‐ABA3’, cysteine desulfurase / selenocysteine lyase; ‘NH_4_^+^’, Ammonium; ‘NIP2;1’, NOD26‐like intrinsic protein 2;1; ‘NiR’, nitrite reductase; ‘NO_2_^−^’, nitrite; ‘NO_3_^−^’, nitrate; ‘NPF2.20’, nitrate/chloride/glucosinolate transporter; ‘NR’, nitrate reductase; ‘NRT1;1B’, nitrate transporter 1.1; ‘O_2_’, oxygen; ‘OAS’, O‐acetyl serine; ‘OAS‐TL’, O‐acetyl serine thiol‐lyase; ‘Oxid.’, oxidation; ‘PAPS’, 3’‐Phospho‐5’‐adenylyl sulfate; ‘PC‐R’, phytochelatin‐conjugate; ‘PCS’, phytochelatin synthase; ‘Phe’, L‐phenylalanine; ‘PSI’, photosystem I; ‘PSII’, photosystem II; ‘pSymA’, symbiotic plasmid A; ‘PT2’, inorganic phosphate transporter; ‘PT4’, inorganic phosphate transporter 1‐4; ‘Pyr’, pyruvate; ‘R’, Generic chemical compound; ‘R‐CH_3_’, methylated compound; ‘ROS’, Reactive oxygen species; ‘SAHH’, adenosylhomocysteinase; ‘SAM’, S‐Adenosylmethionine; ‘SAMS’, S‐adenosylmethionine synthetase; ‘SAT’, serine O‐acetyltransferase; ‘SBP’, selenium‐binding protein; ‘SBP2’, selenocysteine insertion sequence‐binding protein 2; ‘Se’, Selenide; ‘Se_2_Cys’, Thiocysteine (selenolselenocysteine); ‘Sec’, selenophosphate‐derived selenocysteine; ‘SECIS’, selenocysteine insertion sequence; ‘SeCoA’, (seleno)coenzyme A; ‘SeCys’, (seleno)cysteine; ‘SeCys_2_’, (seleno)cystine; ‘SeCysO_2_’, cysteinesulfinate; selenocysteineseleninate; ‘SeCysO_3_’, cysteate; selenocysteinate; ‘SeHCys’, (seleno)homocysteine; ‘SeHCys_2_’, (seleno)homocystine; ‘SeHLan’, (seleno)homolanthionine; ‘SelABCD’, bacterial selenocysteine utilization operon; ‘SeLan’, (seleno)lanthionine; ‘SeMet’, (seleno)methionine; ‘SeMM’, S(e)‐methyl(seleno)methionine; ‘SeMoCo’, (seleno)molybdenum cofactor; ‘SeMoCo‐Se’, selenol/ thio‐(seleno)molybdenum cofactor; ‘SeO_3_^2−^’, sulfite/ selenite; ‘SeO_4_^2−^’, sulfate/ selenate; ‘SepSec’, O‐phospho‐L‐seryl‐tRNASec:L‐selenocysteinyl‐tRNA synthase; ‘Ser’, serine; ‘SeSAH’, S(e)‐adenosyl(seleno)homocysteine; ‘SeSAM’, S(e)‐adenosyl(seleno)methionine; ‘SeThPP’, (seleno)thiamin pyrophosphate; ‘SHMT’, serine hydroxymethyltransferase; ‘SiR’, sulfite reductase; ‘SIRH’, siroheme; ‘SMM’, S‐methylmethionine; ‘SMT’, selenocysteine methyltransferase; ‘SOT’, sulfotransferase; ‘SOX’, sulfite oxidase; ‘SPS’, selenide; water dikinase; ‘SQD1’, UDP‐sulfoquinovose synthase; ‘SQD2’, sulfoquinovosyltransferase; ‘SST1’, symbiotic sulfate transporter 1; ‘ST1;1 ST 1;2’, sulfate transporter 1;1 and 1;2; ‘ST1;3’, sulfate transporter 1;3; ‘ST2;1 ST2;2’, sulfate transporter 2;1 and 2;2; ‘ST3;1 2 3 4’, sulfate transporter 3;1; ‐2; ‐3 and 3;4; ‘ST3;4’, sulfate transporter 3;4; ‘ST4;1 ST4;2’, sulfate transporter 4;1 and 4;2; ‘SUFABCDE‐NFU’, Fe‐S cluster assembly proteins; ‘THI1’, thiazole biosynthetic enzyme; ‘tRNA‐AA’, transfer RNA‐aminoacyl conjugate; ‘Trp’, tryptophan; ‘TRx’, thioredoxin.

**Fig. 3:**
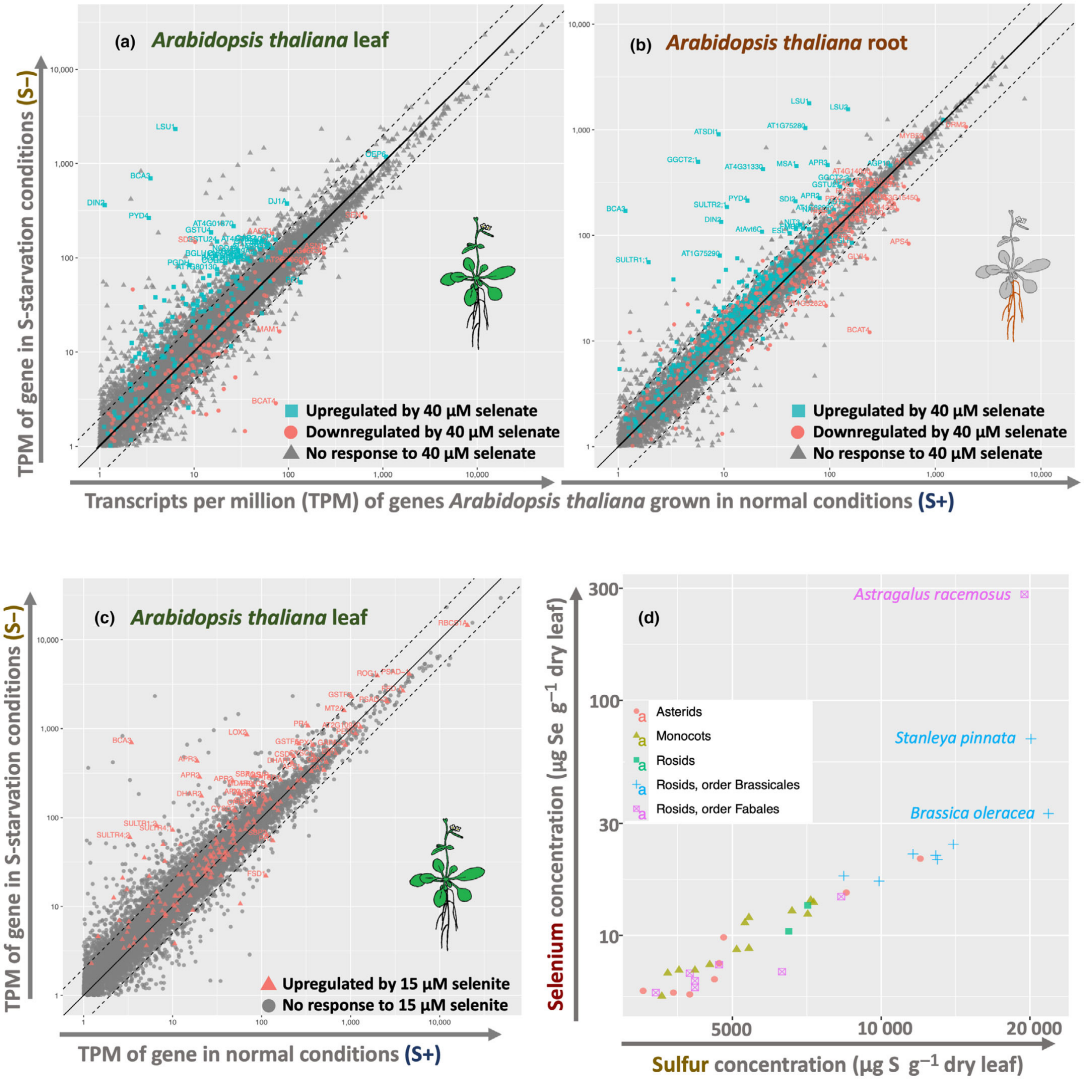
Selenium metabolism in plants shows strong relations with S metabolism, both in transcriptomic responses and elemental uptake patterns across phylogeny. (a–c) Transcriptomic responses of *Arabidopsis thaliana* to sulfur deficiency (S−) and selenium exposure are compared. RNA sequencing data of wild‐type *A. thaliana* Col‐0 from Dietzen *et al*. (2020) were used to plot the expression of *A. thaliana* genes on the *x*‐ and *y*‐axes, indicating their behavior in S− and S+ conditions. (a, b) Data from Van Hoewyk *et al*. (2008) were used to color the datapoints of the genes which were significantly differentially expressed on 40 μM selenate exposure (fold change > 2, *P*‐value < 0.01). The axes are log_10_‐transformed, a solid line is drawn that represents equal transcript per million (TPM) values in S− and S+ conditions, with dotted lines for the twofold difference threshold. The red‐blue separation along the center line indicates the correlation between the S− and selenate response. (c) In a similar plot to (a, b), data from Tamaoki *et al*. (2008) were used to indicate genes upregulated by 15 μM selenite exposure (fold change > 2, *P*‐value < 0.01). With these thresholds, there were no significantly downregulated genes, so only selenite‐upregulated genes are colored. The tendency for red dots to appear above the center line indicates an sulfur (S)‐deficiency‐like response to selenite, although this is less clear as with selenate. (d) The data plotted here are from the common‐garden experiment of White *et al*. (2007), where 39 plant species were grown in identical conditions, exposed to 0.6 μM selenate and 910 μM sulfate, after which the plant S and selenium (Se) content was measured. The datapoints are replotted and colored by phylogenetic order. The highest three values are labelled with the species, of which the two highest values are from known Se hyperaccumulators that break with the overall linear trend between S and Se.

The current consensus is that most plants inadvertently take up and metabolize Se mostly via S pathways (Fig. 2), seemingly lacking any specific Se‐oriented mechanisms for Se processing (Terry *et al*., 2000; White, 2016). This functional mirroring of S metabolism is mainly founded on seven key lines of evidence: (1) many S‐analogous Se compounds have been identified and isolated from plants (Supporting Information Table S1); (2) various S‐converting enzymes can catalyze the analogous Se reaction *in vitro* (Table S2); (3) plants with genetic variants of S transport‐ and metabolism genes, via artificial/natural gene knockout‐ and overexpression mutants, have altered Se uptake and tolerance (Table S3); (4) variation in the expression of S‐assimilation and S‐transport genes is associated with variation in Se tolerance and accumulation (Tamaoki *et al*., 2008; Cabannes *et al*., 2011; Schiavon *et al*., 2015; Wang *et al*., 2018, Table S4); (5) Se and S uptake are interlinked, with S starvation enhancing Se uptake and increased S supplementation reducing the uptake of Se (Parker *et al*., 1992; Shinmachi *et al*., 2010; Cabannes *et al*., 2011; Cardoso *et al*., 2022); (6) Se exposure triggers a S starvation‐like response, leading to increased S uptake by upregulation of S root transporters and enzymes involved in S‐assimilation rate‐limiting steps (Fig. 3a–c; Parker *et al*., 1992; Van Hoewyk *et al*., 2008; Cabannes *et al*., 2011; Grant *et al*., 2011; Kurmanbayeva *et al*., 2022); and (7) the [S]_tissue_ and [Se]_tissue_ concentrations of plants show similar phylogenetic and spatial distribution patterns. On that last note, White *et al*. (2007) grew 39 plant species in identical conditions and observed a clear linear positive relation between [Se]_leaf_ and [S]_leaf_ (Fig. 3d). While this supports the evidence that Se metabolism mirrors that of S, plants also exhibit Se‐related processes that are not analogous to S pathways, for example the nonenzymatic reduction in selenite by thiols, such as the cysteine‐containing tripeptide glutathione (GSH; Ng & Anderson, 1979), which is aided by glutathione reductase activity (Ganther, 1971). This contrasts with the sulfite‐reductase (SiR)‐based assimilation of sulfite in plants (Khan *et al*., 2010). In addition, certain S metabolic pathways appear to lack Se counterparts, such as sulfated compounds and sulfolipids (Nissen & Benson, 1964), likely due to the inability of adenosine phosphosulfate kinase (APK) proteins to generate adenosine 3′phospho 5′selenophosphate (PAPSe; Dilworth & Bandurski, 1977). These pathway differences underline that S and Se have similar chemistry, but that there are crucial differences that contribute to the strong toxicity of Se.

## II. Selenium in plants: toxic or beneficial?

### 1. Selenium toxicity: a concert of organic and inorganic Se stress

Se compounds exert direct cytotoxic effects by their high chemical reactivity, including the direct chemical reaction of Se with thiol groups, especially in its selenite and selenide forms (Tsen & Tappel, 1958; Olm *et al*., 2009), and direct binding or complexation with metals (e.g. mercury and zinc) either in soil or potentially *in planta* (Bai *et al*., 2019; Gui *et al*., 2022b). However, the most prominent effect of direct Se toxicity is through its oxidative properties, which has been documented both *in vitro* and *in planta*, mainly for inorganic forms of Se: selenate (SeO_4_^2−^), selenite (SeO_3_^2‐^) and selenide (Se^2−^) (Nogueira *et al*., 2004; Freeman *et al*., 2010; Grant *et al*., 2011; Kolbert *et al*., 2016). At high tissue concentrations, Se leads to depletion of the glutathione pool, which constitutes an important redox buffer in plants and plays major roles in metal(loid) stress (Grant *et al*., 2011; Hernández *et al*., 2015). Se‐induced oxidative stress in plants manifests in various forms, such as increased levels of reactive oxygen species (ROS; including hydrogen peroxide and superoxide), lipid peroxidation (often measured as increased malondialdehyde content and associated with membrane ruptures) and protein oxidation (mainly Cys, Met, Trp and Tyr residues; Freeman *et al*., 2010; Sabbagh & Van Hoewyk, 2012; Ulhassan *et al*., 2019). Additionally, Se‐induced nitrosative or nitro‐oxidative stress is also described, which includes nitration of protein tryptophan‐ and tyrosine sidechains (Kolbert *et al*., 2019). The generation of oxidative species can also inhibit redox‐sensitive enzymes, such as mitochondrial aconitase (Dimkovikj & Van Hoewyk, 2014), while organic Se compounds can also directly interact with thiol‐containing enzymes, including methionine‐sulfoxide reductase (*msrA* and *msrB*; Sagher *et al*., 2006) and metallothionein (*MT*; Jacob *et al*., 1999).

### 2. Sulfur‐like toxicity: mimicry and misregulation

Another, more distinctive feature of Se toxicity is its interference with S metabolism. Its mimicry to S enables Se to replace S in cofactors and proteins due to ‘colorblind’ enzymes that cannot distinguish between Se and S, leading to the buildup of dysfunctional or malformed proteins where selenocysteine (SeCys) is erroneously inserted at Cys‐codons (Sabbagh & Van Hoewyk, 2012; Van Hoewyk, 2013). In *Stanleya pinnata*, selenate treatment resulted in accumulation of oxidized and ubiquitinated proteins, with the latter containing a relatively high ratio of Se compared with the total protein pool (Sabbagh & Van Hoewyk, 2012), a finding also confirmed in algae (Vallentine *et al*., 2014) and *Brassica napus* (Dimkovikj & Van Hoewyk, 2014). Se also interferes with S metabolism by triggering an S‐deficiency‐like response in plants (Fig. 3a–c). Ironically, this includes the upregulation of sulfate transporters, which are known to also take up selenate and molybdate (El Kassis *et al*., 2007; Shinmachi *et al*., 2010). This suggests that the plant response to toxic levels of selenate would be to take up more selenate. Since S assimilation is tightly co‐regulated with iron metabolism (Courbet *et al*., 2019) and the assimilation of carbon and nitrogen (Jobe *et al*., 2019), this deficiency can have broader consequences. As a result of these toxicity mechanisms and misregulation, ‘higher level’ Se‐toxicity symptoms commonly include inhibition of mitochondrial respiration (Dimkovikj & Van Hoewyk, 2014), reduced photosynthetic efficiency (Freeman *et al*., 2010; Van Hoewyk, 2013), reduced N assimilation (Jain & Gadre, 1998; Sharma, 2017), decreased Chl levels (Jain & Gadre, 1998; Sharma, 2017) and stunted root growth (Zhang *et al*., 2006b; El Kassis *et al*., 2007; Szőllősi *et al*., 2023).

### 3. Selenium growth promotion: a classical example of hormesis?

**Fig. 4:**
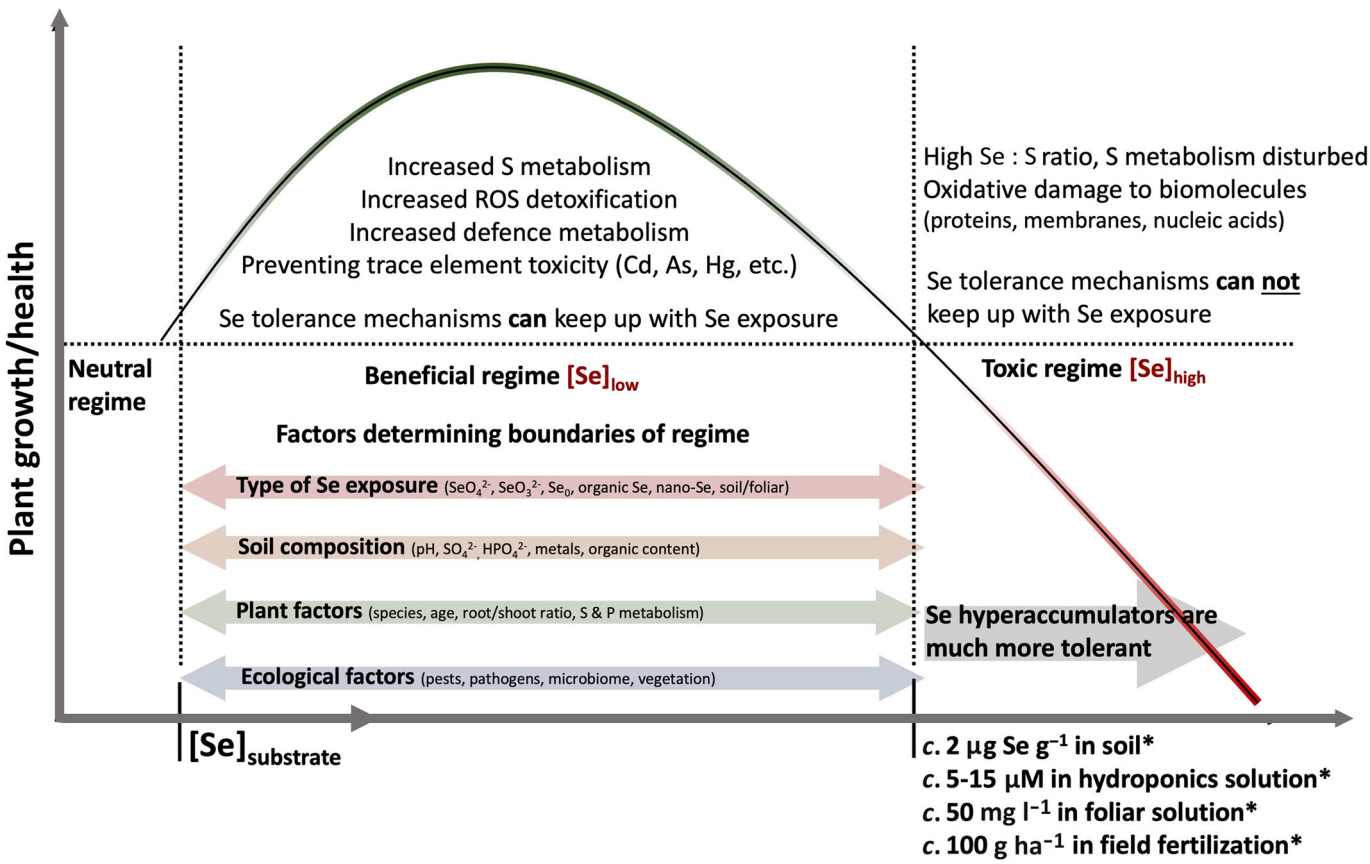
‘Hormesis’ interaction of selenium on plant growth and health is conceptualized. Across a plethora of studies, low levels of Se are shown to be beneficial to plant health and growth, while a too high dose negates or even reverses the effect, reducing plant health and yield. Indicated are factors that influence the Se exposure level that results in beneficial effects, as well as possible underlying factors that account for this double‐edged sword interaction. The values indicated with an asterisk (*) are rough estimates, future studies should define these values further and take into account the four types of factors that determine this range. As, arsenic; Cd, cadmium; Hg, mercury; S, sulfur; Se, selenium. ROS, reactive oxygen species.

In contrast to the toxic consequences of high plant Se exposure, low substrate Se concentrations have consistently been shown to have positive effects on the growth and resilience of various plant species, as reviewed by Lanza & Reis (2021). These beneficial effects can arise from one or more of the following mechanisms: (1) increased activity of antioxidant enzymes that scavenge oxidative chemical species (Ahmad *et al*., 2016; Shahid *et al*., 2019; Lanza & Reis, 2021); (2) direct complexation of Se with metal(loid)s, potentially restricting their transport into shoots (Wang *et al*., 2014; Bai *et al*., 2019; Gui *et al*., 2022b); or (3) increased levels of S compounds that could chelate metals/metalloids (phytochelatins and metallothionein), scavenge ROS (glutathione and thiols), function as cofactors in important pathways (lipoic acid, Fe‐S clusters, biotin, and coenzyme A) or aid in defense (glucosinolates; Khan *et al*., 2015; Gui *et al*., 2022a). On that last note, supplementing plants with Se can yield growth and resilience effects comparable to those achieved with 40‐ to 100‐fold higher levels of applied S, as seen for mercury (Wang *et al*., 2014; Zhong *et al*., 2018) and cadmium stress (Khan *et al*., 2015). In several studies, a distinct dose–response curve has been observed, where a trait is improved at lower Se doses, but this benefit diminishes or even reverses at high Se (Fig. 4; Liu *et al*., 2020; Gui *et al*., 2022a). This phenomenon is also known as ‘hormesis’, wherein exposure to a low level of a potential stressor leads to growth‐promoting effects, often associated with the increased activity of detoxification/ stress response genes (Agathokleous *et al*., 2020). These dynamics suggest that an optimal Se exposure level may exist for each plant species and growth condition.

## III. Selenium in agricultural and ecological settings

These key insights into plant Se physiology mainly derive from experimental studies involving Se supplementation to laboratory‐ or glasshouse‐grown plants or even *in vitro* assays (Parker *et al*., 1992; White *et al*., 2007). While useful for studying the fundamental properties of Se in plants, these might not reflect real‐life dynamics, such as in agricultural settings. Indeed, most natural soils contain very low Se concentrations, on average 0.32 μg Se g^−1^ soil (Jones *et al*., 2017), ranging between 0.01 and 2 μg Se g^−1^ soil in most areas (Pilon‐Smits *et al*., 2017). As a result, the tissue Se concentration of most plants thriving in their natural habitat is extremely low. When examining the leaf Se concentrations of 1097 plant species composited from various literature sources, the median [Se]_leaf_ was 0.1 μg Se g^−1^ (Fig. 1b; Supporting Information Tables S5, S6), which is very low compared with medians for S and phosphorus (P), which are 3600 μg S g^−1^ and 1566 μg P g^−1^. In addition, large elementome screening studies find the majority of wild plant samples below the detection limit for Se (Watanabe *et al*., 2007; Belloeil *et al*., 2021). Therefore, Se plays no significant role in the vast majority of plants and can safely be ignored by most plant scientists. Two important exceptions are Se‐hyperaccumulating plants (Section IV) and agricultural crops, the latter being the major entry point of the essential micronutrient Se in the human diet via crops and animal feed.

### 1. Biofortification: how to get Se from the field to the fork

**Fig. 5:**
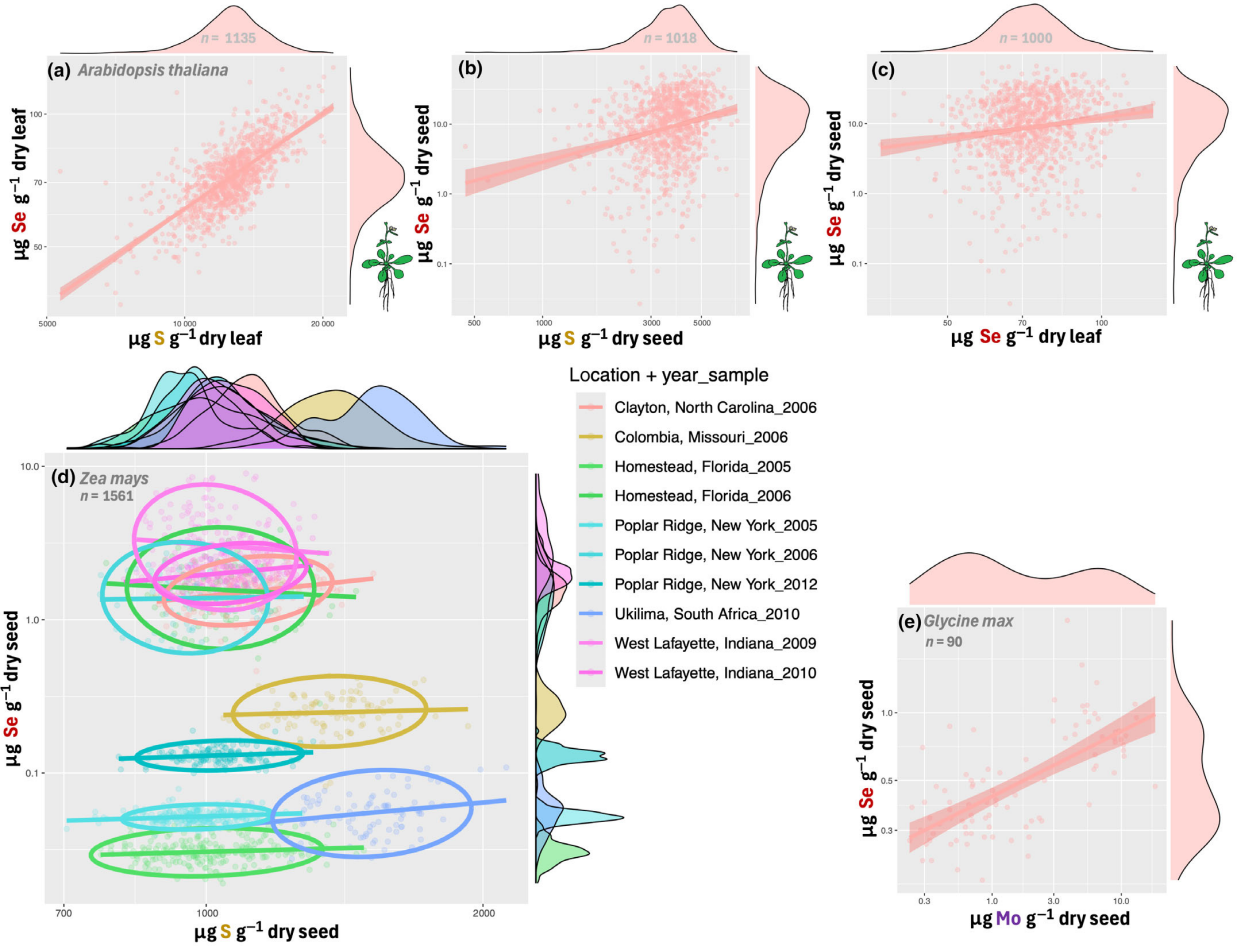
Concentration of Se in leaf and seed tissues of glasshouse‐grown *Arabidopsis thaliana* (a, b & c, data from Campos *et al*., 2021), field‐grown *Zea mays* (d, data from Asaro *et al*., 2016; Fikas *et al*., 2019) and field‐grown *Glycine max* (e, data from Hacisalihoglu & Settles, 2017). The *A. thaliana* plants in Campos *et al*. (2021) were provided with a defined growth medium containing all plant nutrient elements, along with sub‐toxic concentrations of Se, As, Cd, Co, Li, Ni, Sr and Rb, while the field‐grown maize and soybean were not specifically treated and grown under conventional agricultural procedures, mostly in the USA. Trendlines depicted are simple linear regression models and indicated ellipses in (d) represent a 90% confidence level for the multivariate distribution. As, arsenic; Cd, cadmium; Co, cobalt; Hg, mercury; Li, lithium; Ni, nickel; Rb, rubidium; S, sulfur; Se, selenium; Sr, strontium.

We have shown that most plant food products contain very low levels of Se (Fig. 1a). To increase the Se content of crops, it is vital to understand the genetic underpinning of the Se content of edible tissues. As expected from the previous segments, the leaf Se concentrations of 1135 *A. thaliana* accessions (Campos *et al*., 2021) strongly correlated with leaf S concentrations (Fig. 5a), yet more surprisingly this was not true for seed Se and S concentrations (Fig. 5b), and there was little correlation between seed‐ and leaf Se levels of accessions (Fig. 5c). This is important to note since many nutritious parts of crops are seeds, nuts, grains and fruits. Indeed, studies on the elementome of maize kernels (*Zea mays*; Asaro *et al*., 2016; Fikas *et al.*, 2019) also show little relation between kernel S and ‐Se levels (Fig. 5d), the latter of which correlate more strongly with metals, such as zinc (Zn), copper (Cu) and especially molybdenum (Mo). This was also found in a study of the seed elementome of 90 *Glycine max* accessions (Hacisalihoglu & Settles, 2017), where seed Se correlated not with S, but rather Mo (Fig. 5e). Furthermore, a soybean genome‐wide association study by Ziegler *et al*. (2018) found seed Se content affected by a single‐nucleotide polymorphism (SNP) close to an NRAMP3 metal transporter gene and another close to an ABC transporter gene affecting both Se and Fe uptake. In addition, mouse cells (*Mus musculus*) encode a Zn^2+^ ‐ SeO_3_^2−^ cotransporter (ZIP8; McDermott *et al*., 2016), which suggests similar metal‐Se (co)transporters could exist in plants too. However, recent genetic work has also revealed a few P‐ and S homeostasis genes that can positively affect Se seed loading, including a gene encoding a phosphate transporter that increases selenite uptake and seed loading in rice (Yang *et al*., 2025), an O‐acetylserine thiol‐lyase variant (OAS‐TL; one half of cysteine synthase) that increases rice seed S and Se levels (Xu *et al*., 2024) and a serine hydroxymethyltransferase that increased Se uptake and seed loading in rice (Chen *et al*., 2020) as well as maize (Chen *et al*., 2025). It is therefore clear that meaningful Se biofortification (e.g. in edible tissue) does not always have a simple correlation to overall increased S and Se uptake in leaves, but that specific genetic factors of metal, P and S homeostasis can be harnessed to improve this character.

### 2. Walking the tightrope of Se biofortification and toxicity

Another important insight from Asaro *et al*. (2016) and Fikas *et al*. (2019) is that field location and year of harvest had a very strong effect on final seed Se levels (Fig. 5d), likely a result of the sparse and mosaic presence of Se in soils. Therefore, appropriate Se fertilization is a necessary first step toward Se‐enriched crops in most agricultural soils, yet the high toxicity of Se warrants careful research into the safest methods with the highest uptake rates. For example, application of Na_2_SeO_4_ on field‐grown cowpea plants showed a linear increase in seed Se with increasing Se dose from 0 to 150 g Se ha^−1^, yet application rates > 50 g Se ha^−1^ resulted in increased hydrogen peroxide levels and visible toxicity spots on leaves (Lanza *et al*., 2021). Due to their chemical similarity, increasing the availability of sulfate can dampen the uptake and toxicity of selenate, perhaps by alleviating the S‐deficiency response (Fig. 3a–c; Table S7) as shown in *A. thaliana* (Cardoso *et al*., 2023) and in rice hydroponics‐ and field trials, although this also negatively affected the Se content of rice grains (Cardoso *et al*., 2022). Resolving the optimal Se exposure regimes of crops (Fig. 4), although beyond the scope of this review, is a vital counterpart to resolving the genetic basis of Se uptake and metabolization. The latter should ideally breed Se‐tolerant plants (preventing yield loss) that effectively take up Se from the soil (preventing toxic Se fertilizer run‐off) and mobilize this element into the edible tissue (mostly reproductive tissues, ensuring a good Se harvest index). Breeding of such Se adapted crops could benefit from research on so‐called ‘Se hyperaccumulators’, as these plants are naturally highly tolerant to Se (El Mehdawi *et al*., 2014; Szőllősi *et al*., 2023) and have a high Se content (White *et al*., 2007), especially in reproductive tissues (Freeman *et al*., 2006; Harvey *et al*., 2020, 2024b).

## IV. Hypertolerance and accumulation: the arsenal of Se hyperaccumulators

**Fig. 6:**
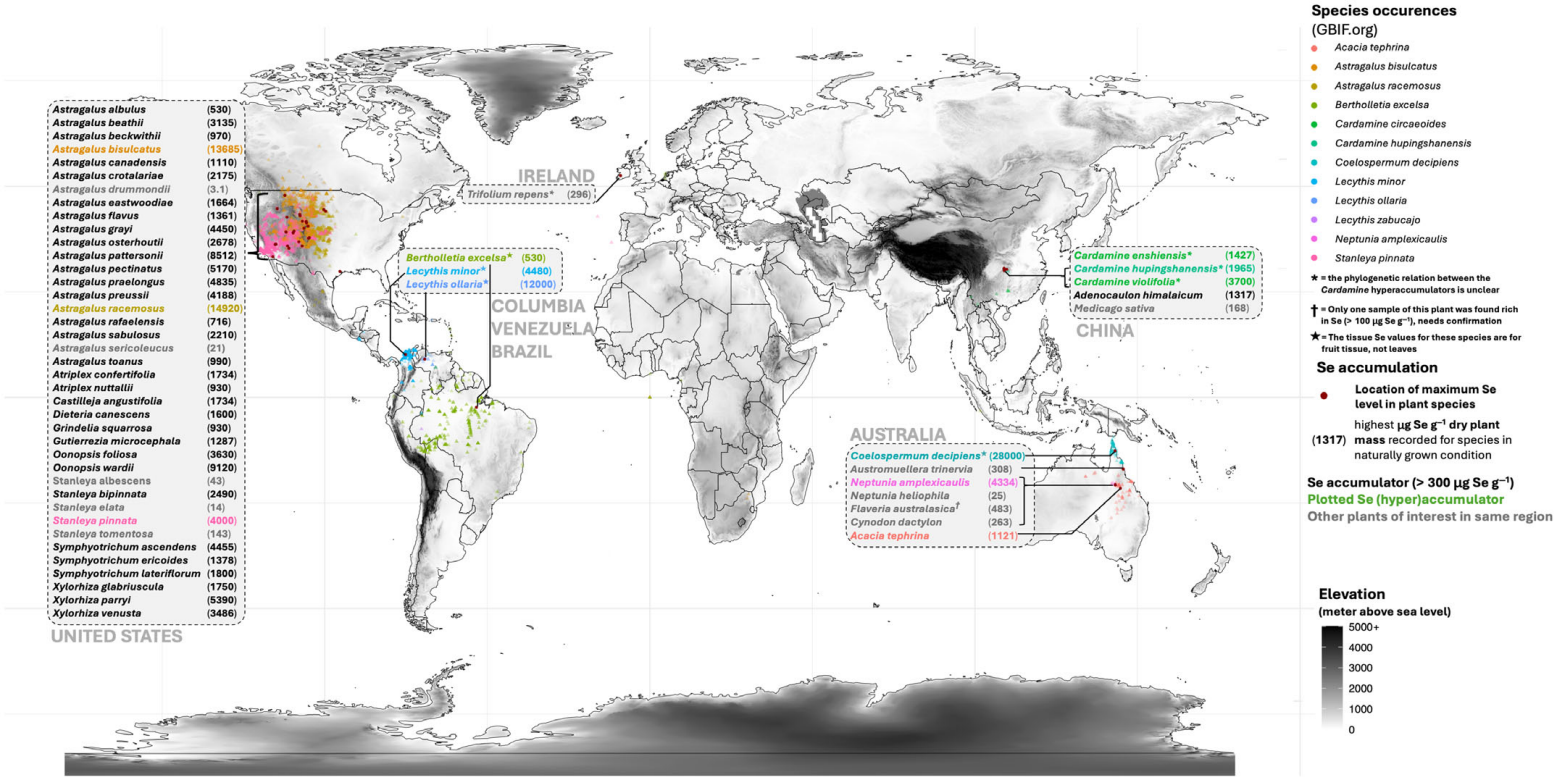
World map depicting the five most well‐studied selenium (Se)‐rich ecosystems on the planet, and for each of these areas, the plants of interest are indicated. Most indicated plants are so‐called selenium hyperaccumulators, but some nonaccumulators growing in these areas are also included upon one of two conditions: (1) a maximum tissue Se level exceeding 100 μg dry weight (DW) (the classical Se accumulator threshold) or (2) when the plant is a close relative of a Se hyperaccumulator in that region, to emphasize the large difference in Se uptake by close family members. The occurrence range of some of the more well‐studied Se hyperaccumulators is plotted using GBIF.org records. The source data and references are available in Supporting Information Table S8.

**Fig. 7:**
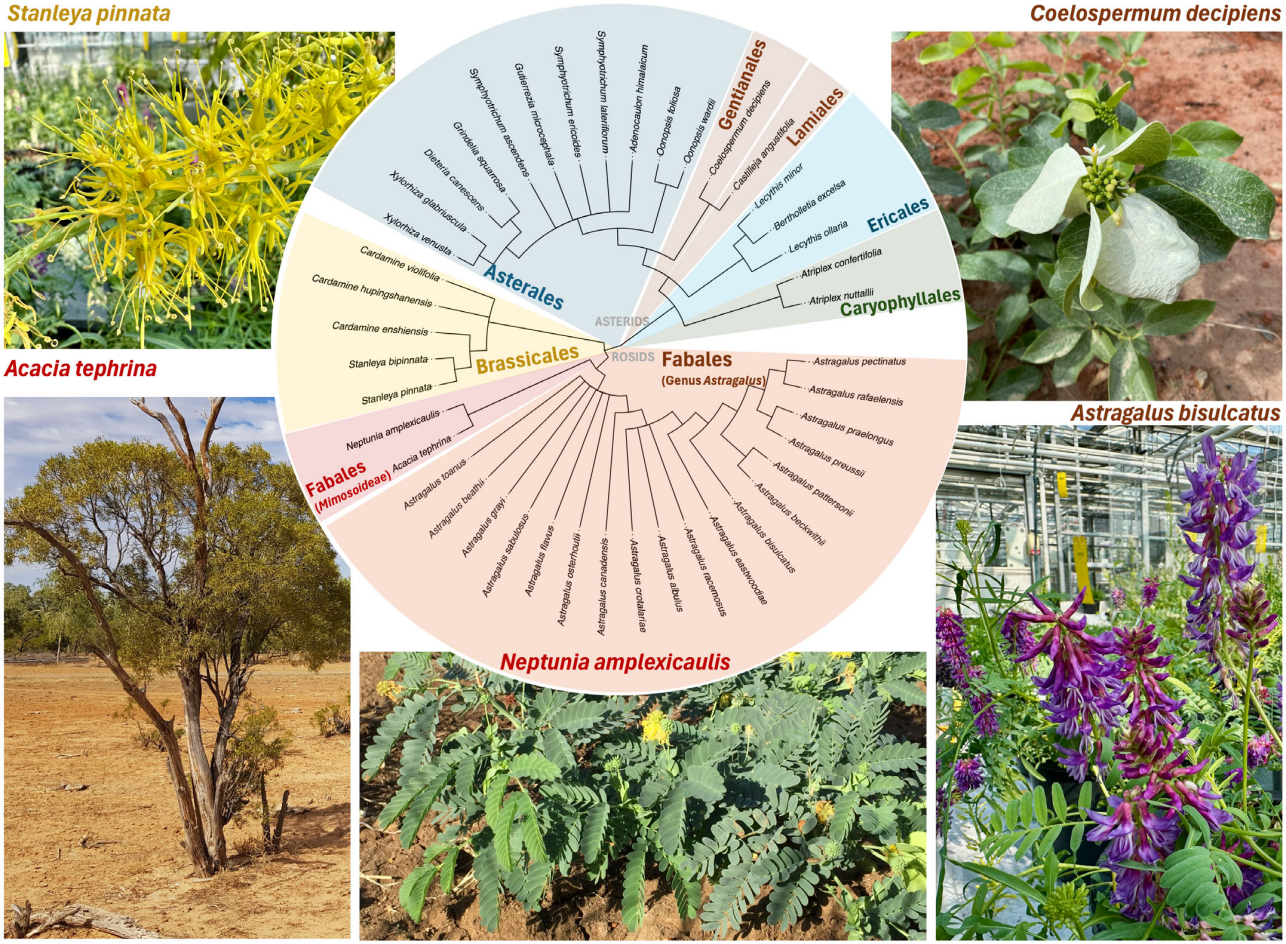
Cladogram of the known selenium (Se)‐hyperaccumulator species. The species known to hyperaccumulate Se are ordered according to their phylogenetic groups, with color codes indicating the orders. Pictures of representative hyperaccumulators are included to emphasize the diversity of Se hyperaccumulators. All images by the authors.

Despite the general scarcity of Se in soils, rare seleniferous patches of the globe can contain up to 69 μg Se g^−1^ soil (Harvey *et al*., 2024a) or even as high as 1265 μg Se g^−1^ soil in extreme cases (McLoughlin *et al*., 2023). Such localities have been identified in the USA (Robinson & Edgington, 1945), Australia (North Queensland; Harvey *et al*., 2024a,b; Knott & McCray, 1959), Ireland (Fleming, 1962; McLoughlin *et al*., 2023) and China (Enshi region; Yuan *et al*., 2013). These seleniferous soils are home to various plant species that tolerate and accumulate exceptionally high Se concentrations, referred to as Se hyperaccumulators (Figs 6, 7; Tables S8, S9). While most plants have [Se]_tissue_ : [S]_tissue_ ratios similar to those in their growth substrate, usually < 1/1000 (White *et al*., 2007), Se hyperaccumulators can reach [Se]_tissue_ concentrations far exceeding 1000 μg Se g^−1^ dry leaf (White, 2016), effectively in the same range as P and S levels (Fig. 1b, outliers of Se concentrations). Among the most well‐studied Se hyperaccumulator species are *Astragalus bisulcatus* and *A. racemosus* (up to 28 500 μg Se g^−1^; Alford *et al*., 2014; van der Ent *et al*., 2023), *Stanleya pinnata* (> 4000 μg Se g^−1^; Freeman *et al*., 2010; Wang *et al*., 2018; van der Ent *et al*., 2023), *Cardamine enshiensis* (1965 μg Se g^−1^; Yuan *et al*., 2013; Zhou *et al*., 2018; Rao *et al*., 2020; Huang *et al*., 2021b) and *Neptunia amplexicaulis* (13 600 μg Se g^−1^; Harvey *et al*., 2020, 2024a; Pinto Irish *et al*., 2021). Decades of studying these hyperaccumulators have found that, in addition to the ‘hormetic’ advantages discussed above, Se‐hyperaccumulator plants can benefit from acquiring much higher concentrations of Se. This has been described as ‘elemental protection’, providing protection against pathogenic fungi, caterpillars (Hanson *et al*., 2003), aphids (Hanson *et al*., 2004), neighboring plants (El Mehdawi *et al*., 2011) and even prairie dogs (Freeman *et al*., 2009). These ecological benefits are hypothesized to be an important evolutionary driving factor of Se hyperaccumulator evolution (Schiavon & Pilon‐Smits, 2017), leading to the emergence and development of a sophisticated arsenal of Se tolerance and accumulation mechanisms. These Se‐hyperaccumulating plant species thus represent excellent models for investigating plant Se uptake, toxicity and tolerance mechanisms, providing examples of genetic adaptations that allow high Se uptake without toxicity. Generally, seven distinct strategies can be identified that contribute to Se tolerance in plants (Fig. S1), which facilitate Se accumulation via upregulation of Se transporters, both root uptake and (vacuolar) storage mechanisms.

### 1. Preventing Se stress: I. modulation of S metabolism and II. enzymatic discrimination between Se and S

Se tolerance and accumulation in plants generally depends on S assimilation and transport activity, respectively (Freeman *et al*., 2010; Cabannes *et al*., 2011; Schiavon *et al*., 2015; Wang *et al*., 2018). High Se uptake is closely associated with the expression of sulfate transporters, which are known to nonspecifically transport selenate (Cabannes *et al*., 2011; Wang *et al*., 2018). The reduction in Se uptake typically occurring at high S availability is often not observed in Se hyperaccumulators (Parker *et al*., 1992; Schiavon *et al*., 2015), which can be explained by their high constitutive expression of sulfate transport and assimilation genes, regardless of external S supply (Cabannes *et al*., 2011; Wang *et al*., 2018). Additionally, enhanced Se tolerance is associated with an increased expression of S‐assimilation genes (Pilon‐Smits *et al*., 1999; Van Huysen *et al*., 2003; Grant *et al*., 2011). This contributes to tolerance by increasing the pool of reduced S metabolites (e.g. cysteine, methionine, GSH), which can buffer the cytotoxicity by improving the critical ratio of Se : S compounds. In addition, the increased activity of the S‐assimilation enzymes (which can moonlight as Se assimilation enzymes) stimulates the conversion of highly oxidative inorganic Se species (Tsen & Tappel, 1958; Grant *et al*., 2011), into organic Se species that can have beneficial redox properties (Sagher *et al*., 2006). Concurrently, the accumulation of inorganic Se species (selenite and selenate) is commonly observed in Se‐sensitive (nonaccumulator) plants, while the ability to predominantly accumulate certain organic Se forms is a characteristic trait of Se hyperaccumulators (Valdez Barillas *et al*., 2012; Lindblom *et al*., 2013b). However, many Se‐sensitive species are also known to accumulate organic Se in the form of selenocysteine (SeCys) and selenomethionine (SeMet; Cai *et al*., 1995; Smrkolj *et al*., 2005), which are known to compete with cysteine (Cys) and methionine (Met) as described above. Therefore, the activity of enzymes that distinguish Se from S is another vital prerequisite to split the Se and S metabolic fluxes, both preventing the unwanted replacement of S by Se in essential metabolic processes and allowing diversion of Se into sequestration/removal pathways while maintaining functional S metabolism. A Se‐specific selenocysteine methyltransferase (SMT) has been found in various Se‐accumulating genera (*Astragalus*, *Stanleya* and *Brassica*; Sors *et al*., 2009). This enzyme methylates SeCys to form Se‐methylselenocysteine (MeSeCys) with a catalytic efficiency *c*. 100‐fold higher than when Cys is used as substrate (Neuhierl & Böck, 1996), and overexpression of SMT enhances plant Se metabolism (LeDuc *et al*., 2004). Due to its larger size compared with SeCys, MeSeCys is not as readily incorporated into proteins, reducing the risk of toxic misincorporation (Burnell & Shrift, 1979). Furthermore, MeSeCys can be degraded to methaneselenol (MeSe), which spontaneously volatilizes to dimethyldiselenide (DMDSe; Gabel‐Jensen *et al*., 2010), a compound responsible for the characteristic smell of Se‐hyperaccumulator plants (Pilon‐Smits *et al*., 2017). MeSeCys can be further converted to gamma‐glutamyl‐Se‐methylselenocysteine (GGMeSeCys), facilitating Se sequestration (Pickering *et al*., 2000; Alford *et al*., 2014) similar to other gamma‐glutamyl‐ligated Se species found in plants (Ogra & Anan, 2012). MeSeCys thus represents a branching point for different Se detoxification processes, steering Se away from essential S processes downstream of cysteine and methionine. As such, MeSeCys biosynthesis serves as a hallmark of Se tolerance and hyperaccumulation (Neuhierl & Böck, 1996; Pickering *et al*., 2003; Sors *et al*., 2009; Freeman *et al*., 2010). The S specificity previously described for APK may also be potentially leveraged. By favoring the S reduction pathway through a Se‐excluding process, APK activity could further enhance the separation of Se and S metabolic fluxes. In *Cardamine enshiensis*, increased expression of 3′‐phosphoadenosine‐5′‐phosphosulfate (PAPS) reductase has been reported, which could potentially be related to this strategy as the Se analog of PAPS is not biosynthesized (Rao *et al*., 2020). The high concentration of selenocystathionine observed in certain Se‐hyperaccumulating plants (*N. amplexicaulis*, *S. pinnata* and *Lecythis ollaria*; Terry *et al*., 2000; Freeman *et al*., 2006) has inspired the hypothesis that their cystathionine beta‐lyase (CBL) enzymes exhibit S specificity, effectively excluding Se (Peterson & Robinson, 1972; Terry *et al*., 2000). However, biochemical studies on these proteins do not currently support this hypothesis (Peterson & Robinson, 1972; McCluskey *et al*., 1986; Dawson & Anderson, 1988, 1989). Nevertheless, the accumulation of selenocystathionine, alongside the low abundance of its S‐analog cystathionine (representing 5% of total cystathionine) in *N. amplexicaulis* (Peterson & Robinson, 1972) suggest a metabolic separation of Se and S at this step. This separation could explain the enhanced Se tolerance of this and other Se‐hyperaccumulating species. Additional Se‐metabolites that are metabolically isolated from the central S metabolism include selenohomolanthionine (Ogra & Anan, 2012), selenolanthionine (Both *et al*., 2018), selenocystine (Yuan *et al*., 2013) and polyselenides (Németh *et al*., 2013). The organic Se compounds most strongly associated with toxicity when accumulated are SeCys and, to a lesser extent, SeMet. This is due to the known substrate promiscuity of methionyl‐tRNA synthetase (MetRS) and cysteinyl‐tRNA synthetase (CysRS) enzymes in most species, which do not discriminate between S amino acids and their Se counterparts (Burnell & Shrift, 1977, 1979; Burnell, 1981a). A notable singular exception is the CysRS enzyme from *A. bisulcatus* which has been found to exclude SeCys from being incorporated into tRNA^cys^ (Burnell & Shrift, 1977; Hoffman *et al*., 2019). However, the Se exclusion of *A. bisulcatus* CysRS does appear to come with a trade‐off, since the binding constant (Km) for cysteine was *c*. threefold higher in comparison with other species (Burnell & Shrift, 1977). These mechanisms that discriminate between S and Se during amino acid metabolism and protein synthesis could explain why Se hyperaccumulators have a lower incorporation of Se into proteins (Németh *et al*., 2013).

### 2. Repairing Se stress: III. Upregulation of protein repair and IV. antioxidant activity

Complete exclusion of seleno‐amino acids from proteins is not observed, even in hyperaccumulator plants with the aforementioned adjustments. Therefore, protein repair and recycling processes are vital to protect plants from Se toxicity (Sabbagh & Van Hoewyk, 2012). Exposure to Se has been shown to increase the activity of heat‐shock proteins, 26S‐ and 20S‐proteasome subunits, ubiquitination‐related genes, chaperones and genes regulating protein degradation, with the more Se‐tolerant plants showing a stronger activity of these processes (Dimkovikj *et al*., 2015; Wang *et al*., 2018; Zhou *et al*., 2018), while 20S proteasome knockouts of *A. thaliana* are more sensitive to Se stress (Sabbagh & Van Hoewyk, 2012). Additionally, *E. coli* appears to allow incorporation of certain inadvertent Se amino acids in noncatalytic residues of proteins, potentially buffering Se toxicity by absorbing some acids (Zorn *et al*., 2013). A similar mechanism might underlie the observation that a high fraction of selenized proteins in rice are seed‐storage proteins, which could act as a ‘safe’ metabolic sink for Se (Cheajesadagul *et al*., 2014). Proteins that lose function or misfold might be marked for proteolysis, while neutral Cys/Met replacements might remain. This principle might underlie the Se‐hyperaccumulation phenotype of Coco de Mono trees (*Lecythis ollaria*), where the leaves contain a meager 16.5 μg Se g^−1^, while the seeds can contain up to 5151 μg Se g^−1^ (Ferri *et al*., 2004), mostly in extremely Se‐rich proteins (Hammel *et al*., 1996). Here, seed‐storage proteins might account for a high level of Se without toxicity to the tree itself.

Another tolerance strategy that directly mitigates toxic effects of Se is the increased expression of the antioxidant machinery. Increased expression of genes encoding ascorbate peroxidases, glutathione peroxidases, catalases, superoxide dismutases and glutathione reductases is associated with reduced ROS levels upon Se exposure and increased Se tolerance (Tamaoki *et al*., 2008; Freeman *et al*., 2010; Wang *et al*., 2018). Moreover, high concentrations of (reduced) glutathione are also associated with enhanced Se tolerance (Freeman *et al*., 2010; Grant *et al*., 2011; Cardoso *et al*., 2023). This association may reflect a dual role for glutathione and glutathione reductase, as they function both as central antioxidants and at the same time are implicated in the assimilation of selenate and selenite into organic Se compounds (Ganther, 1971; Ng & Anderson, 1979; Grant *et al*., 2011).

### 3. Better out than in: V. selenium volatilization, VI. isolation or VII. role of microbiome

Many Se‐(hyper)accumulators volatilize Se in substantial amounts, famously producing a strong, foul, ‘rotten egg’‐smell, as observed in the case of *Astragalus racemosus* plants (S Evans *et al*., 1968). The two main volatile Se compounds are dimethylselenide (DMSe), which is SeMet‐derived (Lewis *et al*., 1974; Tagmount *et al*., 2002) and DMDSe, which originates from MeSeCys (Hall & Smith, 1983; LeDuc *et al*., 2004). DMSe is produced by most Se‐exposed plant species in varying quantities, depending on the activity of enzymes in the *trans*‐sulfuration pathway, which converts cysteine into methionine (Terry *et al*., 1992, 2000). By contrast, DMDSe is the major volatile compound produced by Se hyperaccumulators, due to the diversion of Se into MeSeCys (Pilon‐Smits & LeDuc, 2009). Increased Se volatilization has indeed been linked to greater tolerance to selenite (Van Huysen *et al*., 2003) and reduced tissue Se concentrations (Lewis *et al*., 1974). In addition to volatilization, physical isolation of Se within the plant can also contribute to tolerance. Typically, Se concentrations within plants are highest in the youngest leaves (Pickering *et al*., 2003; Galeas *et al*., 2007), which also exhibit the highest ratios of organic Se (Pickering *et al*., 2003). This pattern has been associated with increased expression of S‐assimilation genes in young leaves compared with old leaves, consistent with source‐sink dynamics (Hawkesford & De Kok, 2007), although total S levels are lower in young leaves compared with old (Pickering *et al*., 2003; Galeas *et al*., 2007). However, micro‐X‐ray fluorescence mapping in Se‐hyperaccumulating species (*Astragalus bisulcatus*, *Astragalus racemosus*, *Stanleya pinnata*, *Cardamine violifolia*, *Acacia tephrina* and *Neptunia amplexicaulis*) has shown that storage of Se in these species is physically separated from the primary plant metabolic zones (Pickering *et al*., 2000; Harvey *et al*., 2020, 2024a,b; van der Ent *et al*., 2023). In these species, Se localized to the vacuoles of epidermal cells at the periphery of the leaves, the apoplastic space, phloem, pulvini, petioles or the leaf vacuoles (Freeman *et al*., 2006; Harvey *et al*., 2020; van der Ent *et al*., 2023). The pattern of apparent ‘spatial exclusion’ of Se from the photosynthetically active compartments of leaf cells likely contributes to avoiding Se toxicity. Comparing the distribution of Se in the Se‐hyperaccumulator species revealed remarkable differences, suggesting that these tolerance mechanisms may have evolved independently, as the phylogenetic distribution also suggests (Fig. 7). For example, aboveground Se in *Stanleya pinnata* was mainly located at the leaf margins, while in *Astragalus racemosus* and *Neptunia amplexicaulis*, Se was mainly in phloem sap and the pulvini (van der Ent *et al*., 2023). Furthermore, seasonal fluctuations in S and Se levels in Se hyperaccumulators show much less correlation as found in nonaccumulators (Galeas *et al*., 2007) and Se hyperaccumulators have also been found to contain comparatively higher Se concentrations in reproductive organs (Freeman *et al*., 2006; Harvey *et al*., 2020, 2024b). These spatio‐temporal patterns of Se accumulation in Se hyperaccumulators point to specialized transportation mechanisms that operate separately for S and Se. These mechanisms are apparently absent in nonaccumulators, and they so far remain poorly understood at the molecular level.

Finally, the plant microbiome is increasingly recognized to contribute to Se accumulation and tolerance. The rhizobiome can influence Se cycling via Se volatilization (De Souza *et al*., 1999), immobilization of Se via the formation of elemental Se or Se nanoparticles (nano‐Se; Valdez Barillas *et al*., 2012; Lindblom *et al*., 2013a,b), oxidation and mobilization of elemental Se (Zhu *et al*., 2021) and the (respiratory) reduction in selenate or selenite and assimilation into organic Se (Schröder *et al*., 1997; Huang *et al*., 2021a). Of special interest is the process of symbiotic nitrogen fixation in Fabaceae root nodules, which are known to require large quantities of metals and S, and to express specialized sulfate transporters (Courbet *et al*., 2019). In *Lotus japonicus*, nitrogen‐fixing nodules have been identified as major production sites of reduced S compounds, effectively shifting the plant S assimilation toward the nodules (Kalloniati *et al*., 2015). This shift in S metabolism suggests that nodulation could change plant Se physiology. Indeed, nodulation of hyperaccumulator *Astragalus bisulcatus* significantly increased shoot Se concentrations, which was not observed for nonaccumulating *Astragalus drummondii* (Alford *et al*., 2014), and the speciation of Se also shifted toward increased accumulation of elemental Se and gamma‐glutamyl‐methylselenocysteine (Valdez Barillas *et al*., 2012; Alford *et al*., 2014). Unfortunately, no hyperaccumulator‐associated Rhizobiales strains have been isolated to study this process in more detail (Alford *et al*., 2014). Therefore, mechanistic insights into the role of this highly complex process in Se metabolism remain limited.

## V. Digging deeper: open questions in plant Se physiology

Although plant Se metabolism has gained increasing attention over the last decade, there are still some fundamental questions that need more research. First among them is the absence of any identified plant protein that specifically functions as a Se transporter: proteins that either uniquely transport Se compounds or possess a strong (10‐ or 100‐fold) preference for Se compounds over P‐ or S‐analogs. Current evidence indicates that sulfate transporters (ST, encoded by the *SULTR*‐gene family in *Arabidopsis thaliana*) are involved in selenate transport (El Kassis *et al*., 2007), selenite is moved by phosphate (PHT) transporters, silicon transporters and aquaporins (Zhao *et al*., 2010; Zhang *et al*., 2014a; Song *et al*., 2017), while organic Se compounds are transported via amino acid permeases LHT1, NPF2.20 and NRT1;1B (Wang *et al*., 2018; Zhang *et al*., 2019; Hu *et al*., 2025). However, none of these transporters exhibit specificity to Se compounds. While these transporters can account for Se uptake in nonaccumulator plants, the markedly high Se : S ratio in Se‐hyperaccumulator species suggests the existence of a transport process specific for Se (White *et al*., 2007). This selectivity could either be due to: (1) specific Se uptake at the root‐soil interface; (2) specific within‐plant translocation favoring Se over S; or (3) differential (root) assimilation pathways for Se and S, leading to the use of different transporter classes (e.g. sulfate transporter vs amino acid transporters). Regarding the first two options, research has often focused on known transporters that carry amino acid sequence variation that shifts the specificity from S to Se. It is proposed that specialized sulfate transporter genes in *Astragalus* spp. may have evolved to preferentially take up selenate (Cabannes *et al*., 2011), although this hypothesis remains to be verified. As for the third mechanism, selective uptake may be driven by differences in Se and S redox chemistry. For instance, in human cancer cells, selenite uptake was shown to occur in a reducing extracellular environment, where selenite is first reduced to selenide by extracellular Cys before being imported (Olm *et al*., 2009). This reduction pathway appears specific for Se, as analogous reduction in sulfite mediated by thiols does not occur. Similar interplay between Se reduction and uptake could provide a mechanism of selective Se transport in plants too. Of interest to this hypothesis, the Se‐hyperaccumulator *S. pinnata* has higher root activity of S‐assimilation genes compared with *S. elata* and *S. albescens* (Freeman *et al*., 2010; Wang *et al*., 2018). This suggests that root Se assimilation can provide tolerance by converting the highly mobile and toxic selenate to less toxic organic Se forms and perhaps account for a shifted Se : S ratio when the rate or endpoint metabolite of Se‐ and S‐assimilation processes are not equal.

### 1. Selenium assimilation: dependent on S assimilation?

For two decades, selenate reduction has been considered the rate‐limiting step for plant Se assimilation, requiring the investment of ATP via adenosinephosphosulfatase (APS‐synthase; Pilon‐Smits *et al*., 1999) to form adenosinephosphoselenate (APSe; Burnell, 1981b). In concert with this, it is often observed that selenate is relatively mobile and easily transported from root to shoot, while the chemically more unstable selenite is retained and metabolized in the roots. Indeed, selenite and selenate tolerance correlated little among 19 *A. thaliana* accessions and separate genetic loci were associated with these two phenotypes (Zhang *et al*., 2006a, 2007), perhaps reflecting this different localization and/or relating to the transporter associated with these forms (previous segment). While there is strong evidence that APS‐synthase can positively affect Se assimilation and tolerance (Tables S2, S3), either by directly catalyzing selenate reduction or via increased thiol biosynthesis and increased S metabolic flux, there is little evidence that SeO_4_^2−^ reduction strictly requires this enzyme. The conversion of selenate to selenite has a mild +0.05 V redox potential, while the conversion of sulfate to sulfite has a staggering −0.93 V redox potential, requiring the ATP‐consuming step to bring the latter down to −0.06 V (Wessjohann *et al*., 2007; Abdulina *et al*., 2020; de Bang *et al*., 2021). This asymmetry in redox potential opens up the possibility for selenate reduction independent of sulfate reduction, as was indeed found in dedicated bacterial selenate reductase enzymes (Schröder *et al*., 1997) and bacterial nitrate reductases (Sabaty *et al*., 2001; Bébien *et al*., 2002), neither of which reduce sulfate. The plant assimilatory nitrate reductase (NR) has not been tested for selenate reductase activity, while NR is known to moonlight as chlorate reductase (Wilkinson & Crawford, 1993) and nitrite to nitrous oxide reductase (Chamizo‐Ampudia *et al*., 2017). Although it remains to be confirmed in plants, this would allow for a sulfate assimilation pathway‐independent selenate reduction (NR, other enzymes?) and selenite reduction (GSH, thiols). In this hypothesis, S and Se metabolism would converge from cysteine synthase onward, as it is indeed understood in *Escherichia coli* (Turner *et al*., 1998).

### 2. Unorthodox selenocompounds: diversity and unknown significance

In addition to the well‐characterized Se species found in plants, such as amino acid SeCys and SeMet, some more uncommon species of Se have been identified, although so far they remain poorly studied (Table S1). For example, selenosugars have been identified in cereals, garlic, *Astragalus racemosus* and *A. bisulcatus* (Cai *et al*., 1995; Aureli *et al*., 2012; Szőllősi *et al*., 2023). Next to this, Se is known to occur in the inorganic form of selenosulfate in plants, an analog of thiosulfate (Vonderheide *et al*., 2006). Thiosulfate is involved in various metabolic processes. In particular, it serves as the unique substrate for S‐Sulfocysteine Synthase (CS26), which has been associated with tolerance to long‐day light‐derived oxidative stress (Bermúdez *et al*., 2010). Selenocyanate is another selenocompound produced by green algae (Wang *et al*., 2024) and can be abundant in certain soil types. Notably, *Brassica juncea* has been shown to take up, detoxify and metabolize selenocyanate (De Souza *et al*., 2002), although the underlying metabolic pathway remains uncharacterized. Similarly, it is not clear whether uptake and metabolization of atmospheric Se species is relevant in plants. By analogy, when sulfate uptake in the roots is blocked or reduced, the uptake of carbonyl‐sulfide (COS) and its assimilation increases via carbonic anhydrase (CA) activity (Maruyama‐Nakashita *et al*., 2003; Liu *et al*., 2021), and plants can also use atmospheric H_2_S as the sole S source (Buchner *et al*., 2004). The biosynthesis of Se analogs of secondary S compounds like allicins, polysulfanes and glucosinolates has long been debated (Bertelsen *et al*., 1988), but metabolomic studies have since confirmed the presence of these selenocompounds and their breakdown products in various plants (Matich *et al*., 2012; Németh *et al*., 2013). Finally, trimethylselenium, which is an abundant selenocompound in urine, has been found to be taken up but not metabolized by plants (Olson *et al*., 1976; Rayman *et al*., 2008). The plant microbiome might also play important roles in this respect, as the production or consumption of some Se species in the plant holobiont might be predominantly carried out by plant‐commensal bacteria (De Souza *et al*., 1999). A major challenge in elucidating the identity of selenocompounds is their inherent chemical instability, which hinders analytical efforts despite the advancement of the analysis techniques.

### 3. Selenium signaling: how do plants sense‐ and respond to Se?

As previously discussed, Se exposure elicits various effects on plant physiology (Table S4), most strikingly, triggering responses characteristic of S deficiency. Yet, the precise mechanism by which plants sense and respond to Se remains unclear. GSH, which is implicated in many aspects of Se metabolism, has long been regarded as a major signaling molecule for S‐status. However, *A. thaliana* mutants with impaired GSH synthesis (*rax1*, *cad2*) do not upregulate important S‐assimilation genes (e.g. *APR2*; Lee *et al*., 2012) and GSH breakdown enzymes are upregulated in S deficiency (Dietzen *et al*., 2020). Therefore, the S‐deficiency response might result in a low level of GSH, rather than the reverse. Various genes involved in S assimilation are redox‐regulated (Jez, 2019), and various ROS‐generating stresses (such as exposure to Cu, Cd, peroxide or selenate) are shown to activate the S‐deficiency pathway (Rouached *et al*., 2008; Hugouvieux *et al*., 2009), therefore, another link between S regulation and GSH might be its role as an antioxidant. The strong interaction between GSH and Se could explain the similarities between selenate exposure and S deficiency (Fig. 3a,b). Various *A. thaliana* ET and JA signaling mutants exhibit higher sensitivity to selenite, a phenotype linked to a reduced activation of S‐assimilation and antioxidant defense genes (Tamaoki *et al*., 2008). Studies on *AtSULTR1;2* suggest this transporter may function as a transceptor acting both in sulfate transport and in sensing S‐status (Zhang *et al*., 2014b; Takahashi, 2019). This dual role could help explain why selenate has a stronger positive effect on S uptake compared with selenite, which sometimes even reduces S levels (Ríos *et al*., 2008; Tian *et al*., 2017), and why only the *A. thaliana sultr1;2* mutant, among all *sultr* mutants, show increased Se tolerance (El Kassis *et al*., 2007). According to this theory, selenate could bind to the sulfate‐receptor site of SULTR1;2, making it blind to the actual sulfate concentration, leading to a false S starvation signal. Yet, this mechanism remains hypothetical for now, and needs to be validated. Downstream components of the S‐deficiency signaling pathway, such as the transcription factors SULFUR LIMITATION 1 (SLIM1) and various ethylene response factors, are also likely to be involved in Se‐responses (Tamaoki *et al*., 2008; Dietzen *et al*., 2020). By analogy, SLIM1 has been shown to be essential for tolerance to the metalloid arsenic in *A. thaliana* (Jobe *et al*., 2021), and ETHYLENE RESPONSE FACTOR 96 overexpression has been reported to increase selenite tolerance (Jiang *et al*., 2020b). An epigenetic contribution to the S‐ response has been identified in the *MORE SULPHUR ACCUMULATION* (MSA1) gene in *A. thaliana*, which modulates the expression of S‐related genes via alterations in DNA methylation (Huang *et al*., 2016). Notably, a mutation in the rice MSA1 homolog (*OsCADT1*) has recently been shown to increase Se uptake (Chen *et al*., 2020), suggesting an overlap in genetic regulation of S and Se metabolism. However, the regulation of Se metabolism in Se‐hyperaccumulating species remains largely unresolved and is likely to differ drastically from that in other, non‐hyperaccumulating, plants (Schiavon & Pilon‐Smits, 2017). The possibility of a specific Se sensor in Se hyperaccumulators should be considered, especially given the observation of Se‐specific root foraging behavior in *N. amplexicaulis* (Pinto Irish *et al*., 2021) and *S. pinnata* (Goodson *et al*., 2003). Additional, specialized Se regulatory processes are also likely to exist. For example, *Cardamine enshiensis* exhibits chromatin rearrangements in genomic regions associated with Se metabolism‐related genes upon Se treatment, suggesting epigenetic regulation of Se hyperaccumulation (Huang *et al*., 2021b). So far, the regulatory mechanisms of Se metabolism in hyperaccumulators remain unclear, particularly in how they differ from those in non‐hyperaccumulators. It has been suggested that Se hyperaccumulators have a constitutive S‐deficient status, resulting in continuous high expression of sulfate transporters and S‐assimilation genes (Cabannes *et al*., 2011; Wang *et al*., 2018).

### 4. Selenium utilization in plants: fact or myth?

Ever since Se was first found in plants, its potential function in plant physiology has been debated (Trelease & Trelease, 1938). The current consensus is that, while microalgae utilize Se in extensive selenoproteomes (Novoselov *et al*., 2002; Jiang *et al*., 2020a), vascular plants do not encode true selenoproteins (Santesmasses *et al*., 2017) and therefore do not require Se. Evolutionary remnants of Sec utilization can still be found in plant genomes, such as transfer RNAs for SeCys (tRNA^Sec^; Santesmasses *et al*., 2017) and even a selenocysteine insertion sequence (SECIS) in the mitochondrial DNA of American cranberry (*Vaccinium macrocarpon*; Fajardo *et al*., 2014). However, there is no evidence that vascular plants produce selenoproteins, or indeed require Se in their life‐cycle, which resonates with the extremely low Se content of most plants (Fig. 1b). Recent findings of Sec utilization in some ancient fungal lineages (Mariotti *et al*., 2019) and the shrunken 2‐member selenoproteome of invertebrate *Rhodnius prolixus* (Mesquita *et al*., 2015) further highlight the gradual, mosaic loss of Se utilization along the tree of life. Se scarcity in most terrestrial ecosystems is a likely evolutionary driving factor of Sec utilization loss, as indeed algae inhabiting the Se‐rich saltwater ecosystems generally encode more selenoproteins (Jiang *et al*., 2020a). However, this scarcity would perhaps not be an evolutionary driving factor in the Se‐rich ecosystems of Se hyperaccumulators (Fig. 6), and these plants are known to show a strong Se‐induced growth benefit. The first genome of a Se hyperaccumulator (*Cardamine enshiensis*) has recently been assembled (Huang *et al*., 2021b), and several RNA sequencing experiments on these plants are conducted in the last 7 years (Table S4). In addition, recently developed proteomic techniques have identified so‐called ‘facultative selenoproteins’ in mammals (Guo *et al*., 2018; Jedrychowski *et al*., 2020), which broadens the potential physiological functions of Se. These recent developments warrant a revisit of Se utilization to either discard this possibility or to find a true physiological role for Se in vascular (hyperaccumulator) plants.

## VI. Conclusions and outlook

Some promising opportunities for future research can be found in the following directions:

1. Mapping Se across phylogenetic‐ and ecological space: by leveraging high‐throughput ionomic sampling (Belloeil *et al*., 2021), phylogenetic predispositions for Se uptake can be probed. When combined with geological‐ and climatic data (Jones *et al*., 2017), this can be expanded to include geo‐ecological factors, gaining insight into what environmental factors affect Se uptake. This might also identify novel Se hyperaccumulators and Se‐rich ecosystems.
2. Genome‐scale studies of Se hyperaccumulators: while a large number of (multi)‐omics studies have been used recently to study Se dynamics in crop species (Table S4), the amount of ‐omics data on Se hyperaccumulators is scarce. Only one genome has been assembled (*Cardamine enshiensis*) and seven full transcriptome studies (six on *Cardamine* spp. and 1 on *S. pinnata*, see Table S4) have been performed to our knowledge. Characterizing additional Se hyperaccumulators via genomics and transcriptomics studies (especially in comparison with nonaccumulating species or ecotypes) will expand the understanding of the molecular aspects of Se tolerance‐ and uptake mechanisms (Fig. 7).
3. Defining the plant selenoproteome and metabolome: applying the recently developed Selenoproteomics methods to the proteomes of Se hyperaccumulators might identify (SECIS‐independent) selenoproteins. Similarly, DNA‐ or RNA sequencing of these high Se plants might identify characteristic signs of relict selenoproteomes. In addition, applying untargeted quantitative metabolomics approaches (e.g. HPLC‐MS–MS; Ogra & Anan, 2012) to Se dosing experiments will allow for a more complete picture of the chemical diversity of Se in plants and aid in the development of *in silico* metabolic models to providing more insight into the phytochemistry of Se.
4. Spatial attributes of Se tolerance and accumulation: Synchrotron‐based X‐ray fluorescence techniques (Pushie *et al*., 2014) can be used to make elemental maps at very high resolution, allowing for detailed studies on the Se distribution in Se hyperaccumulator‐ and crop organs and tissues.
5. Resolving Se exposure regimes in practical settings: the Se hormesis interaction suggests that every plant has a beneficial range of Se exposure. A meta‐analysis of available studies of Se‐exposed plants and additional dosing trials should allow us to resolve Se dosage levels beneficial to plant growth, considering the Se form, growth conditions and plant species. In addition, such trials could map the genotypic variation in Se tolerance and uptake in crop species (Table S10) to inform breeding programs and provide practical information for agriculturalists to produce Se‐enriched crops, as most plant food products are currently low in Se (Table S11). Ideally, field trials could also resolve the influence of environmental variables (precipitation, drought, UV, herbivory etc.) on the uptake and tolerance to Se in a real‐world setting. Only through such an integrated approach of fundamental science on real‐life field trials can we fully understand and responsibly harness this essential poison.

## Competing interests

None declared.

## Author contributions

JW wrote the first version of this manuscript. MGMA, MS and AvdE edited the manuscript.

## Disclaimer

The New Phytologist Foundation remains neutral with regard to jurisdictional claims in maps and in any institutional affiliations.

## Supporting information


**Fig. S1** Complete SeATLAS scheme in the full interactive .pdf form, where underlined items are hyperlinks to KEGG/UniProt/similar databases.

**Table S1** Table of various selenocompounds identified in plant tissues.

**Table S2** List of enzymes from plants (and/or fungi) that are shown to convert seleno‐analogs of sulfur compounds.

**Table S3** Table of genetic mutations that show a significant effect on plant Se metabolism, tolerance and/or accumulation.

**Table S4** Table summarizing 83 transcriptomics, metabolomics, proteomics and genomics studies on plant Se metabolism.

**Table S5** Source data for determining phylogenies of plant species in Figs 1(b) and 3(d), retrieve from https://treeoflife.kew.org/.

**Table S6** Source data for Figs 1(b) and 3(d), containing 47 000 elemental compositions of plants from 39 literature sources.

**Table S7** Source data for Fig. 3(a–c) acquired from three transcriptomics studies of *Arabidopsis thaliana* (Tamaoki *et al*., 2008; Van Hoewyk *et al*., 2008; Dietzen *et al*., 2020).

**Table S8** Source data for Figs 6 and 7, containing the location, Se content and species name of Se‐accumulating plants and close relatives.

**Table S9** Source data for Figs 6 and 7, containing the occurrence data of Se‐accumulating‐plants as retrieved from https://www.gbif.org.

**Table S10** Source data for Fig. 5, retrieved for the most part from www.ionomicshub.work courtesy of Dr Ivan Baxter and Prof. David Salt.

**Table S11** Source data for creating Fig. 2(a) acquired from ‘NEVO online version 2023/8.0, RIVM, Bilthoven’ (https://www.rivm.nl/documenten/nevo‐online‐versie).
Please note: Wiley is not responsible for the content or functionality of any Supporting Information supplied by the authors. Any queries (other than missing material) should be directed to the *New Phytologist* Central Office.
