# Supplementary material for: An atlas of plant selenium metabolism
Source: New Phytol. 2026 Mar 16;250(4):2041–60. doi: 10.1111/nph.71087 (PMC13103439; doi:10.1111/nph.71087)
Supplement: Supplementary file 1 — Fig. S1 Complete SeATLAS scheme in the full interactive .pdf form, where underlined items are hyperlinks to KEGG/UniProt/similar databases. [file NPH-250-2041-s009.pdf]

SEATLAS V1

© Jeroen van der Woude

PLANT SELENIUM ACCUMULATION, TOLERANCE, LOCALIZATION & ASSIMILATION SCHEME (FEBRUARY 2026)

If you ignore the red parts, this can be a plant sulfur metabolism chart. With a toxic Secret.

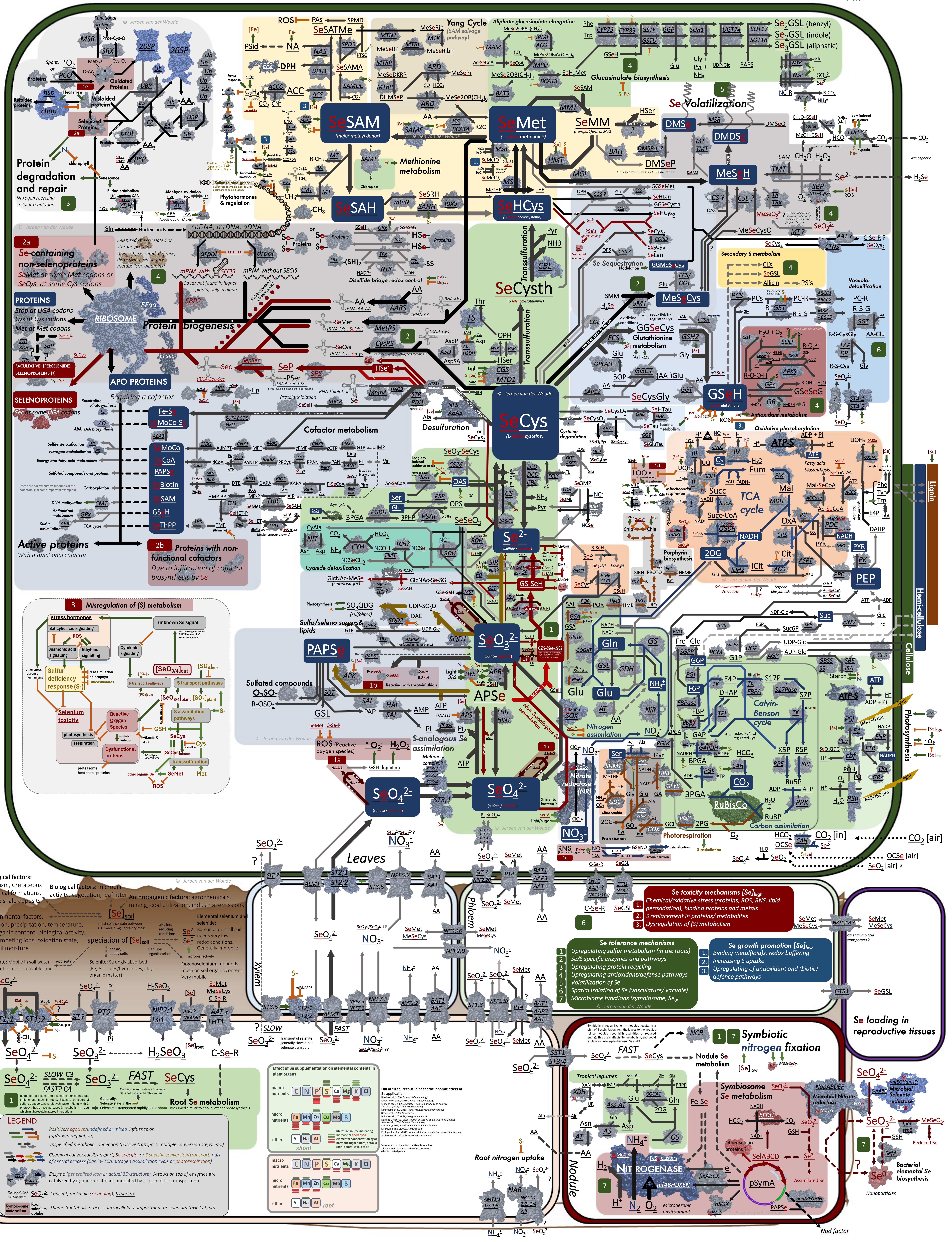

New Phytologist Supporting Information

Article title: "An Atlas of plant selenium metabolism"

Authors: Jeroen van der Woude, Mark G. M. Aarts, Michela Schiavon & Antony van der Ent

Article acceptance date: 14 February 2026

Supplementary Figure S1. The plant Selenium Accumulation, Tolerance, Localization & Assimilation Scheme (Plant SeATLAS). This scheme provides a comprehensive representation of selenium metabolism based on studies of plant Se metabolism, studies of the plant S metabolism, Se metabolism in other kingdoms of life (mammalian, microbial) and especially the work done on Se hyperaccumulator plants. The red color is indicative of the S-Se analogy, with the red part showing the Se analogue of the S compound, red arrows indicating Se-specific (e.g. non-sulfur) conversions and molecular processes, and yellow arrows indicating S processes so far not known to be mirrored by Se. When the red parts are ignored, this can be used as a plant sulfur metabolic chart. Mechanisms of Se tolerance and accumulation are also shown, along with the most important carbon- and nitrogen fluxes in the plant seeing that these are vital in the broader metabolism. A simplified representation of nodule metabolism is also included since various Se-accumulators are *Fabaceae*, and nodulation affects both Se and S metabolism. Protein structures indicated with lighter blue represent the actual 3D-structure of the protein, while dull-grey colored protein structures are simple placeholders (e.g. most transporters are represented using the Nodulin 26-like Intrinsic Protein (NIP2;1, an aquaporin) structure). For the explanation of all the abbreviations, see the supplementary pdf file of this figure. Recommended use: open the file in a web browser and using "ctrl+left click" on the hyperlinks in the figure to learn more about the compounds and gene products.
