# Supplementary material for: An atlas of plant selenium metabolism
Source: New Phytol. 2026 Mar 16;250(4):2041–60. doi: 10.1111/nph.71087 (PMC13103439; doi:10.1111/nph.71087)
Supplement: Supplementary file 2 — Table S1 Table of various selenocompounds identified in plant tissues. [file NPH-250-2041-s012.docx]

**New Phytologist Supporting Information**
**Article title:** “An Atlas of plant selenium metabolism”
**Authors:** Jeroen van der Woude, Mark G. M. Aarts, Michela Schiavon & Antony van der Ent
**Article acceptance date:** 14 February 2026

**Table S1.** List of selenocompounds identified in plant tissues. The plant species and sources for these are not exhaustive, since many of these compounds are found across many plant species. **Abbreviations: AFS:** atomic fluorescence spectrometry**, AED:** atomic emission detector**, CIMS:** chemical ionization mass spectrometry **, ESI:** electron spray ionization, **GC:** gas chromatography**, HG:** hydride generation, **HPLC:** high performance liquid chromatography**, ICP:** inductively coupled plasma **, LC:** liquid chromatography **, MS:** mass spectrometry**, qTOF:** quadrupole time-of-flight **, TOF:** time-of-flight, **UV:** ultraviolet

| **name** | **Selenocompound** | **Plant species** | **Technique** | **Sources** |
| --- | --- | --- | --- | --- |
| SeO_4_^2-^ | Selenate | *Cucurbita pepo, Pisum sativum* | HPLC-UV-HG-AFS | (Smrkolj *et al.*, 2006b,a) |
| SeO_3_^2-^ | Selenite | *Cucurbita pepo, Pisum sativum* | HPLC-UV-HG-AFS | (Smrkolj *et al.*, 2006b,a) |
| SeCys | Selenocysteine | *Allium sativum, Brassica juncea* | HPLC-UV-HG-AFS, GC-AED | (Cai *et al.*, 1995; Smrkolj *et al.*, 2005, 2006a) |
| SeMet | Selenomethionine | *Allium sativum, Brassica juncea* | HPLC-UV-HG-AFS, GC-AED | (Cai *et al.*, 1995; Smrkolj *et al.*, 2005, 2006a) |
| SeCys_2_ | Selenocystine | *Cucurbita pepo, Allium sativum, Brassica juncea* | HPLC-UV-HG-AFS, GC-AED | (Smrkolj *et al.*, 2005, 2006a; Vonderheide *et al.*, 2006) |
| MeSeCys | Se-methyl-selenocysteine | *Pisum sativum, Allium sativum, Brassica juncea* | HPLC–UV–HG-AFS, GC-AED | (Cai *et al.*, 1995; Smrkolj *et al.*, 2006a; Ruszczyńska *et al.*, 2017) |
| DMSe | Dimethylselenide | *Brassica juncea, Allium sativum* | GC-AED | (Cai *et al.*, 1995) |
| DMDSe | Dimethyldiselenide | *Brassica juncea, Allium sativum, Brassica nigra* | GC-AED | (Cai *et al.*, 1995) Ouerdane 2013 |
| SeSO_3_ | Selenosulfate | *Brassica juncea* | HPLC-ICP-MS and  ESI-qTOF-MS | (Vonderheide *et al.*, 2006) |
| SeGSL | Selenoglucosinolate (and derivatives thereof) | *Stanleya pinnata, Brassica oleraceae, Brassica napus* | GC/CIMS | (Bertelsen *et al.*, 1988; Matich *et al.*, 2012; Ouerdane *et al.*, 2013) |
| SeSAM | Se-adenosylselenomethionine | *Triticum aestivum* | HPLC-ESI-MS-MS | (Ogra & Anan, 2012) |
| SeMetO | Selenomethionine-Se-oxide | *Allium sativum* | ﻿HPLC-ICP-MS | (Ruszczyńska *et al.*, 2017) |
| GGMeSeCys | 𝞬-glutamyl methylselenocysteine | *Allium sativum* | HPLC-ICP-MS | (Ruszczyńska *et al.*, 2017) |
| Tyr-SeMet | Tyrosine-selenomethionine dipeptide | *Bertholla excelsa* | HPLC-ICP-MS and ES-MS | (Vonderheide *et al.*, 2002) |
| SeMM | Se-methyl-selenomethionine | *Trifolium pratense*, *Astragalus spp., Brassica juncea* | ESI-MS | (Shrift, 1969; Grant *et al.*, 2004) |
| GSeH | Selenoglutathione (and derivatives of) | *Allium sativum*, *Helianthus annuus* | HPLC-ICP-MS | (Ruszczyńska *et al.*, 2017) |
| MeGSeH | Methyl-selenoglutathione | *Allium sativum* | HPLC-ICP-MS | (Ruszczyńska *et al.*, 2017) |
| GlcNAc-MeSe | Methylseleno monosaccharide | *Brassica rapa*, *Triticum aestivum, Zea mays, Oryza sativa, Astragalus racemosus* | HPLC-ESI-MS | (Aureli *et al.*, 2012; Ouerdane *et al.*, 2013) |
| Glc_2_MeSe | Methylseleno disaccharide | *Allium sativum* | HPLC-ICP-MS | (Ruszczyńska *et al.*, 2017) |
| SeCysth | Selenocystathionine | *Neptunia amplexicaulis* | LC | (Peterson & Butler, 1967) |
| GGSeCysth | γ-glutamylselenocystathionine | *Lecythis minor* | HPLC-ESI-qTOF | (Ogra & Anan, 2012) |
| SeHLan | Selenohomolanthionine | *﻿Raphanus sativus, Allium sativum* | ESI-MS-MS | (Ogra & Anan, 2012; Ruszczyńska *et al.*, 2017) |
| SeHCys | Selenohomocysteine (and derivatives) | *Astragalus crotalariae, Lecythis minor* | ESI-qTOF-MS | (Brown & Shrift, 1982; Németh *et al.*, 2013) |
| SeHCys_2_ | Selenohomocystine | *Astragalus crotalariae, Lecythis minor* | ESI-qTOF-MS | (Shrift, 1969; Németh *et al.*, 2013) |
| SeLan | Selenolanthionine | *Cardamine violifolia* | LC-ESI-qTOF-MS | (Both *et al.*, 2018) |
| MeSeCN | Methylselenomethanonitrile | *Brassica oleracea* | GC-MS | (Matich *et al.*, 2012) |
| DMSeP | Dimethylselenoproprionate | *Brassica juncea* | TOF-MS | (Grant *et al.*, 2004) |
| SeO_2_Cys | Selenocysteine-seleninic acid | *Trifolium pratense, Lolium perenne* | Paper chromatography & paper, ion exchange & paper electrophoresis | (Peterson & Butler, 1962) |
| Seleno-wax | Seleno-wax | *Stanleya pinnata* | Chemical extraction, infrared spectra, radioactive labelling | (McColloch *et al.*, 1963) |
| 2,3DHP-SeCys | ﻿2,3-Dihydroxypropionyl-selenocysteine-cysteine  (and derivatives) | *Raphanus sativus, Helianthus annuus* | HPLC-ESI-MS-MS | (Ruszczyńska *et al.*, 2017) |
| 2,3DHP-SeLan | ﻿2,3-Dihydroxypropionyl-selenolanthionine | *Raphanus sativus* | HPLC-ESI-MS-MS | (Ruszczyńska *et al.*, 2017) |
| NCSe | Selenocyanate | *Brassica nigra* | HPLC-ESI-MS | (Ouerdane *et al.*, 2013) |

**References**

**Aureli F, Ouerdane L, Bierla K, Szpunar J, Prakash NT, Cubadda F**. **2012**. Identification of selenosugars and other low-molecular weight selenium metabolites in high-selenium cereal crops. *Metallomics* **4**: 968–978.

**Bertelsen F, Gissel-Nielsen G, Ki˦r A, Skrydstrup T**. **1988**. Selenoglucosinolates in nature: Fact or myth? *Phytochemistry* **27**: 3743–3749.

**Both EB, Shao S, Xiang J, Jókai Z, Yin H, Liu Y, Magyar A, Dernovics M**. **2018**. Selenolanthionine is the major water-soluble selenium compound in the selenium tolerant plant *Cardamine violifolia*. *Biochimica et Biophysica Acta - General Subjects* **1862**: 2354–2362.

**Brown TA, Shrift A**. **1982**. Selenium: Toxicity and Tolerance in Higher Plants. *Biological Reviews* **57**: 59–84.

**Cai XJ, Block E, Uden PC, Zhang X, Quimby BD, Sullivan JJ**. **1995**. Allium Chemistry: Identification of Selenoamino Acids in Ordinary and Selenium-Enriched Garlic, Onion, and Broccoli Using Gas Chromatography with Atomic Emission Detection. *Journal of Agricultural and Food Chemistry* **43**: 1754–1757.

**Grant TD, Montes-Bayón M, Leduc D, Fricke MW, Terry N, Caruso JA**. **2004**. Identification and characterization of Se-methyl selenomethionine in *Brassica juncea* roots. *Journal of Chromatography A* **1026**: 159–166.

**Matich AJ, McKenzie MJ, Lill RE, Brummell DA, McGhie TK, Chen RKY, Rowan DD**. **2012**. Selenoglucosinolates and their metabolites produced in Brassica spp. fertilised with sodium selenate. *Phytochemistry* **75**: 140–152.

**Ogra Y, Anan Y**. **2012**. *Selenometabolomics Explored by Speciation*.

**Ouerdane L, Aureli F, Flis P, Bierla K, Preud’Homme H, Cubadda F, Szpunar J**. **2013**. Comprehensive speciation of low-molecular weight selenium metabolites in mustard seeds using HPLC-electrospray linear trap/orbitrap tandem mass spectrometry. *Metallomics* **5**: 1294–1304.

**Peterson PJ, Butler GW**. **1967**. Significance of selenocystathionine in an Australian selenium-accumulating plant, *Neptunia amplexicaulis* [22]. *Nature* **213**: 599–600.

**Ruszczyńska A, Konopka A, Kurek E, Torres Elguera JC, Bulska E**. **2017**. Investigation of biotransformation of selenium in plants using spectrometric methods. *Spectrochimica Acta - Part B Atomic Spectroscopy* **130**: 7–16.

**Shrift A**. **1969**. Aspects of Selenium Metabolism in Higher Plants. *Annual Review of Plant Physiology* **20**: 475–494.

**Smrkolj P, Germ M, Kreft I, Stibilj V**. **2006a**. Respiratory potential and Se compounds in pea (*Pisum sativum L*.) plants grown from Se-enriched seeds. *Journal of Experimental Botany* **57**: 3595–3600.

**Smrkolj P, Stibilj V, Kreft I, Germ M**. **2006b**. Selenium species in buckwheat cultivated with foliar addition of Se(VI) and various levels of UV-B radiation. *Food Chemistry* **96**: 675–681.

**Smrkolj P, Stibilj V, Kreft I, Kapolna E**. **2005**. Selenium species determination in selenium-enriched pumpkin (*Cucurbita pepo L.*) seeds by HPLC-UV-HG-AFS. *Analytical Sciences* **21**: 1501–1504.

**Vonderheide AP, Mounicou S, Meija J, Henry HF, Caruso JA, Shann JR**. **2006**. Investigation of selenium-containing root exudates of *Brassica juncea* using HPLC-ICP-MS and ESI-qTOF-MS. *Analyst* **131**: 33–40.

**Vonderheide AP, Wrobel K, Kannamkumarath SS, B’Hymer C, Montes-Bayón M, De León CP, Caruso JA**. **2002**. Characterization of selenium species in Brazil nuts by HPLC-ICP-MS and ES-MS. *Journal of Agricultural and Food Chemistry* **50**: 5722–5728.
