# Supplementary material for: An atlas of plant selenium metabolism
Source: New Phytol. 2026 Mar 16;250(4):2041–60. doi: 10.1111/nph.71087 (PMC13103439; doi:10.1111/nph.71087)
Supplement: Supplementary file 3 — Table S2 List of enzymes from plants (and/or fungi) that are shown to convert seleno‐analogs of sulfur compounds. [file NPH-250-2041-s005.docx]

**New Phytologist Supporting Information**

**Article title:** “An Atlas of plant selenium metabolism”

**Authors:** Jeroen van der Woude, Mark G. M. Aarts, Michela Schiavon & Antony van der Ent

**Article acceptance date:** 14 February 2026

**Table S2.** List of enzymes from plants (and/or fungi) that are shown to convert seleno-analogues of sulfur compounds.

| **Gene product** | **Species** | **Notes** | **Sources** |
| --- | --- | --- | --- |
| Cysteinyl-tRNA synthetase | *Neptunia amplexicaulis* |  | (Burnell, 1981a; Birringer *et al.*, 2002) |
| Methionyl-tRNA synthetase | *Neptunia amplexicaulis, Phasoleus aureus* |  | (Burnell, 1981a,b; Birringer *et al.*, 2002) |
| Cystathionine beta-lyase | *Astragalus sinicus*, *Neptunia amplexicaulis* |  | (Dawson & Anderson, 1988; Birringer *et al.*, 2002) |
| ATP sulfurylase | *Neptunia amplexicaulis* |  | (Burnell, 1981a; Birringer *et al.*, 2002) |
| Cystathionine gamma-synthase | *Astragalus racemosus, Astragalus sinicus, Spinacia oleracea* |  | (Dawson & Anderson, 1989; Birringer *et al.*, 2002) |
| Cysteine synthase | *Astragalus sinicus* |  | (Birringer *et al.*, 2002) |
| Cysteine lyase | *Arabidopsis thaliana* |  | (Pilon-Smits *et al.*, 2002) |
| Selenocysteine methyltransferase | *Astragalus bisulcatus* |  | (Neuhierl & Böck, 1996) |
| Sulfite oxidase | *Arabidopsis thaliana* | Only 5% of sulfite activity for selenite | (Hemann *et al.*, 2005) |
| S-adenosylmethionine synthase | *Saccharomyces cerevisae* |  | (Mudd & Cantoni, 1957) |
| Methionine-gamma lyase | *Arabidopsis thaliana* |  | (Goyer *et al.*, 2007) |

**References**

**Birringer M, Pilawa S, Flohé L. 2002.** Trends in selenium biochemistry. *Natural Product Reports* 19: 693–718.

**Burnell JN**. **1981a**. Selenium Metabolism in *Neptunia amplexicaulis*. *Plant Physiology* **67**: 316–324.

**Burnell JN**. **1981b**. Methionyl-tRNA Synthetase from *Phaseolus aureus*: Purification and Properties. *Plant Physiology* **67**: 325–329.

**Dawson JC, Anderson JW**. **1988**. Incorporation of cysteine and selenocysteine into cystathionine and selenocystathionine by crude extracts of spinach. *Phytochemistry* **27**: 3453–3460.

**Dawson JC, Anderson JW**. **1989**. Comparative enzymology of cystathionine and selenocystathionine synthesis of selenium-accumulator and non-accumulator plants. *Phytochemistry* **28**: 51–55.

**Goyer A, Collakova E, Shachar-Hill Y, Hanson AD**. **2007**. Functional characterization of a methionine γ-lyase in *Arabidopsis* and its implication in an alternative to the reverse trans-sulfuration pathway. *Plant and Cell Physiology* **48**: 232–242.

**Hemann C, Hood BL, Fulton M, Hänsch R, Schwarz G, Mendel RR, Kirk ML, Hille R**. **2005**. Spectroscopic and kinetic studies of *Arabidopsis thaliana* sulfite oxidase: Nature of the redox-active orbital and electronic structure contributions to catalysis. *Journal of the American Chemical Society* **127**: 16567–16577.

**Mudd SH, Cantoni GL**. **1957**. Selenomethionine in Enzymatic Transmethylations. *Nature* **180**: 1052–1052.

**Neuhierl B, Böck A**. **1996**. On the mechanism of selenium tolerance in selenium-accumulating plants. Purification and characterization of a specific selenocysteine methyltransferase from cultured cells of *Astragalus bisculatus*. *European Journal of Biochemistry* **239**: 235–238.

**Pilon-Smits EAH, Garifullina GF, Abdel-Ghany S, Kato SI, Mihara H, Hale KL, Burkhead JL, Esaki N, Kurihara T, Pilon M**. **2002**. Characterization of a NifS-like chloroplast protein from *Arabidopsis* . Implications for its role in sulfur and selenium metabolism. *Plant Physiology* **130**: 1309–1318.
