# Supplementary material for: An atlas of plant selenium metabolism
Source: New Phytol. 2026 Mar 16;250(4):2041–60. doi: 10.1111/nph.71087 (PMC13103439; doi:10.1111/nph.71087)
Supplement: Supplementary file 4 — Table S3 Table of genetic mutations that show a significant effect on plant Se metabolism, tolerance and/or accumulation. [file NPH-250-2041-s008.docx]

**New Phytologist Supporting Information**

**Article title:** “An Atlas of plant selenium metabolism”

**Authors:** Jeroen van der Woude, Mark G. M. Aarts, Michela Schiavon & Antony van der Ent

**Article acceptance date:** 14 February 2026

**Table S3.** List of genetic mutations that show a significant effect on plant Se metabolism, tolerance and/or accumulation. **Abbreviations: P:** phosphorous, **S:** sulfur, **Se:** selenium

| **Gene product** | **Type of genetic variant** | **Plant species** | **Effect on plant** | **Source** |
| --- | --- | --- | --- | --- |
| Sulphate transporter (ST) | Overexpression (ST1;2)  *st1;2* knockout  *st1;1* or *st1;2* knockout | *Brassica juncea*  *Arabidopsis thaliana*  *Arabidopsis thaliana* | Increased [Se]_leaf_  Increased [SeO_4_^2-^] tolerance  Increased [SeO_4_^2-^] tolerance | (El Kassis *et al.*, 2007; Barberon *et al.*, 2008; Ohno *et al.*, 2012) |
| ATP sulfurylase | Overexpression | *Arabidopsis thaliana*  *Brassica juncea*  *Nicotiana benthamiana* | Decreased [SeO_4_^2-^] tolerance, decreased total [Se]_leaf_, increased organic [Se]_leaf_  Increased [SeO_4_^2-^] tolerance  Increased total and organic [Se]_leaf_ | (Pilon-Smits *et al.*, 1999; Sors *et al.*, 2005; McKenzie *et al.*, 2009) |
| Adenosine 5’-phosphosulphate reductase | Overexpression  Natural variant  Knockout | *Arabidopsis thaliana*  *Arabidopsis thaliana*  *Arabidopsis thaliana* | Increased [SeO_4_^2-^] tolerance, decreased total [Se]_leaf_, increased organic [Se]_leaf_  Increased [Se]_leaf_  Increased [Se]leaf and [S]leaf, increased selenate/selenite ratio | (Sors *et al.*, 2005; Grant *et al.*, 2011; Chao *et al.*, 2014) |
| Glutathione synthetase | Overexpression  Knockout | *Brassica juncea*  *Arabidopsis thaliana* | Increased [SeO_4_^2-^] tolerance, increased total [Se]leaf  Did not change [SeO_3_^2-^] tolerance | (Bañuelos *et al.*, 2005; Aborode *et al.*, 2016) |
| 𝞬-glutamyl-cysteine synthetase | Overexpression  Null-mutant (*cad2-1*)  Null-mutant (*pad2-1*) | *Brassica juncea*  *Arabidopsis thaliana*  *Arabidopsis thaliana* | Increased total [Se]leaf  Decreased [SeO_4_^2-^] substrate tolerance  Decreased [SeO_4_^2-^] substrate tolerance, partially alleviated by external glutathione supply | (Bañuelos *et al.*, 2005; Hugouvieux *et al.*, 2009; Cardoso *et al.*, 2023) |
| Serine-acetyltransferase | Overexpression | *Arabidopsis thaliana* | Decreased total [Se]leaf, increased organic [Se]leaf | (Sors *et al.*, 2005) |
| Cystathionine-𝞬-synthase | Overexpression | *Brassica juncea* | Increased [SeO_3_^2-^] tolerance, decreased [SeO_3_^2-^] uptake, increased Se volatilization | (Van Huysen *et al.*, 2003, 2004) |
| Selenocysteine methyltransferase | Overexpression | *Arabidopsis thaliana*  *Brassica juncea*  *Nicotiana tabacum* | Increased [SeO_3_^2-^]and [SeO_4_^2-^] tolerance, increased [Se]leaf, increased [Se] volatilization, increased fraction of methylselenocysteine and gamma-glutamylmethylselenocysteine | (Ellis *et al.*, 2004; LeDuc *et al.*, 2004, 2006; Bañuelos *et al.*, 2007; Kubachka *et al.*, 2007; Matich *et al.*, 2009; McKenzie *et al.*, 2009) |
| Cysteine lyase | Overexpression | *Arabidopsis thaliana* | Increased [SeO_4_^2-^] tolerance, increased [Se]leaf, decreased [Se] in protein | (Van Hoewyk *et al.*, 2005) |
| Selenocysteine lyase | Overexpression | *Arabidopsis thaliana*  *Brassica juncea* | Decreased [SeO_3_^2-^] and [SeO_4_^2-^] tolerance for chloroplast-expression gene, increased tolerance for cytosol-expressed gene  Total [Se]leaf increased | (Pilon *et al.*, 2003; Garifullina *et al.*, 2003; Bañuelos *et al.*, 2007) |
| Selenium-binding protein | Overexpression | *Arabidopsis thaliana* | Increased [SeO_3_^2-^] tolerance | (Agalou *et al.*, 2005) |
| O-acetyl serine thiol-lyase (cysteine synthase) | Knockout  Natural variant, gain-of-function mutant | *Arabidopsis thaliana*  *Oryza sativa* | Decreased [SeO_4_^2-^] tolerance  Natural variamt: Increased uptake and assimilation of Se and S, increased As tolerance  Gain-of-function mutant: increased sulfate transporter and serine acetyl transferase activity, increased sulfate and thiol concentration, increased rice seed S and Se levels | (Sun *et al.*, 2021; Kurmanbayeva *et al.*, 2022; Xu *et al.*, 2024) |
| 26S proteasome (regulatory particle) | Knockout | *Arabidopsis thaliana* | Decreased [SeO_4_^2-^] tolerance | (Sabbagh & Van Hoewyk, 2012) |
| Methionine methyl transferase | Knockout | *Arabidopsis thaliana* | Almost complete loss of Se volatilization | (Tagmount *et al.*, 2002) |
| Phosphate transporter | Overexpression  Knockout | *Nicotiana tabacum*  *Oryza sativa* | Increased [Se]leaf and P uptake | (Zhang *et al.*, 2014; Song *et al.*, 2017) |
| Peroxidase | Overexpression | *Arabidopsis thaliana* | Increased [SeO_3_^2-^] tolerance | (Jiang *et al.*, 2015) |
| COQ5 methyltransferase | Overexpression | *Arabidopsis thaliana* | Reduced selenate uptake, increased volatilization from selenite \| note: has some similarity to inorganic selenium methyltransferases from bacteria^335^ | (Zhou *et al.*, 2009) |
| Silicon influx transporter (LSI1) | Knockout | *Oryza sativa* | Decreased uptake of selenite but not selenate | (Zhao *et al.*, 2010) |
| Transcription factor WRKY47 | Overexpression  Knockout | *Arabidopsis thaliana* | Increased uptake of selenite  Decreased tolerance to selenite | (Wu *et al.*, 2020) |
| S-adenosylmethionine synthase | Knockout | *Saccharomyces cerevisae* | Increased tolerance to selenomethionine application, S-adenosylmethionine-auxotrophy | (Malkowski *et al.*, 2007) |
| Ascorbate peroxidase (APX) | Natural variant (loss of function) | *Arabidopsis thaliana* | Increased tolerance to SeO_3_^2-^, increased root growth, increased activity of glutathione-based antioxidant system | (Jiang *et al.*, 2016) |
| Ethylene response factor 96 (ERF96) | Overexpression | *Arabidopsis thaliana* | Increased SeO_3_^2-^ tolerance; increased anti-oxidant activity | (Jiang *et al.*, 2020) |
| Putative serine hydroxymethyltransferase (SHMT7/MSA1) | Natural loss of function (rice)  Knockout mutant (maize) | *Oryza sativa*  *Zea mays* | Rice: Increased SeO_4_^2-^ and SO_4_^2-^ uptake, reduced cadmium root-shoot translocation, increased Se seed loading  Maize: increased leaf Se and S, increased SO_4_ and glutathione, increased kernel Se and amino acids, increased expression of sulfate transport- and assimilation genes. | (Chen *et al.*, 2020, 2025) |
| Homocysteine methyltransferase | Overexpression | *Arabidopsis thaliana* | Increased tolerance to SeO_4_^2-^ , increased [S], reduced expression of some S-assimilation genes | (Chen *et al.*, 2024) |
| Peptide transporter NRT1.1B | Overexpression | *Oryza sativa* | Increased translocation of SeMet from roots to shoot | (Zhang *et al.*, 2019) |
| Terpenoid synthase (TPS22) | Loss of function | *Arabidopsis thaliana* | Increased tolerance to SeO_3_^2-^  Decreased transcription of phosphate transporters (PHT1;1, PHT1;8 and PHT1;9), higher expression of selenocysteine methyl transferase (SMT)  Lower cytokinin levels | (Jiang *et al.*, 2018) |
| E3 ubiquitin ligases (HRDA1 HRDB1) | Knock-out | *Arabidopsis thaliana* | Decerased tolerance to SeO_4_^2-^ treatment, | (Van Hoewyk, 2018) |
| Isopentyltransferase (*ipt1357)* | Quadruple knockout | *Arabidopsis thaliana* | Enhanced SeO_3_^2-^ tolerance  Decreased transcription of phosphate transporters (PHT1;1, PHT1;8 and PHT1;9), higher expression of selenocysteine methyltransferase. Higher activity of catalase, ascorbate peroxidase and glutathione peroxidase, higher glutathione level  Reduced cytokinin levels, reduced Se content | (Jiang *et al.*, 2019) |
| Cytokinin receptor (*ahk2 ahk3*) | Single knockouts | *Arabidopsis thaliana* | Reduced SeO_3_^2-^ tolerance  Increased transcription of phosphate transporters (PHT1;1, PHT1;8 and PHT1;9),  Increased cytokinin levels, increased Se content | (Jiang *et al.*, 2019) |
| Ethylene resposne factor (RAP2-6) | Overexpression | *Arabidopsis thaliana* | Mutants were more tolerant to SeO_3_^2-^ treatment. Lower Se uptake, lower phosphate transporter PHT1;4 expression, | (Tang *et al.*, 2023) |
| ﻿Nitrate transporter 1/peptide transporter family (BrNPF2.20) homolog of AT1G69870 | Overexpression | *Arabidopsis thaliana* | Overexpression of *Brassica rapa* BrNPF2.20 increased SeO_3_^2-^ uptake | (Hu *et al.*, 2025) |
| Ethylene insensitive 2 (*ein2*) & Ethylene insensitive 3 (*ein3)* | Knockout mutant | *Arabidopsis thaliana* | Decreased tolerance to SeO_3_  Decreased tolerance to SeO_4_ | (Tamaoki *et al.*, 2008)  (Van Hoewyk *et al.*, 2008) |
| 1-aminocyclopropane-1-carboxylate synthase 6 (*acs6*) | Knockout mutant | *Arabidopsis thaliana* | Decreased tolerance to SeO_3_  Decreased tolerance to SeO_4_ | (Tamaoki *et al.*, 2008)  (Van Hoewyk *et al.*, 2008) |
| Ethylene-overproduction protein 1(*eto1*) | Knockout mutant | *Arabidopsis thaliana* | Increased tolerance to SeO_3_ | (Tamaoki *et al.*, 2008) |
| Jasmonoyl--L-amino acid synthetase (*jar1;* jasmonic acid biosynthesis) | Knockout mutant | *Arabidopsis thaliana* | Decreased tolerance to SeO_3_  Decreased tolerance to SeO_4_ | (Tamaoki *et al.*, 2008)  (Van Hoewyk *et al.*, 2008) |
| Mannose-1-phosphate guanylyltransferase 1 (*vtc1;* ascoribic acid biosynthesis) | Knockout mutant | *Arabidopsis thaliana* | Decreased tolerance to SeO_3_ | (Tamaoki *et al.*, 2008) |
| Ethylene-responsive transcription factor 1B (ERF1) | Overexpression | *Arabidopsis thaliana* | Increased tolerance to SeO_4_ | (Van Hoewyk *et al.*, 2008) |
| Phosphate transporter 4 (*OsPT4)* | Knockout- and overexpression | *Oryza sativa* | Knockout reduced root-shoot translocation of SeO_3_ and seed Se loading. Also reduced biomass more than half with or without Se treatment. Overexpression in increased biomass and Se uptake rate. In yeast and frog egg cell culture takes up selenomethionine and SeO_3_ | (Yang *et al.*, 2025) |
| *Enhanced suberin1 (AtESB1)* | Knockout mutant | *Arabidopsis thaliana* | Mutants of this gene have altered root suberin barriers and increased levels of shoot S and Se | (Baxter *et al.*, 2009) |

**References**

**Aborode FA, Raab A, Voigt M, Costa LM, Krupp EM, Feldmann J. 2016.** The importance of glutathione and phytochelatins on the selenite and arsenate detoxification in *Arabidopsis thaliana*. *Journal of Environmental Sciences (China)* 49: 150–161.

**Agalou A, Roussis A, Spaink HP**. **2005**. The *Arabidopsis* selenium-binding protein confers tolerance to toxic levels of selenium. *Functional Plant Biology* **32**: 881–890.

**Bañuelos G, Leduc DL, Pilon-Smits EAH, Terry N**. **2007**. Transgenic Indian mustard overexpressing selenocysteine lyase or selenocysteine methyltransferase exhibit enhanced potential for selenium phytoremediation under field conditions. *Environmental Science and Technology* **41**: 599–605.

**Bañuelos G, Terry N, Leduc DL, Pilon-Smits EAH, Mackey B**. **2005**. Field trial of transgenic Indian mustard plants shows enhanced phytoremediation of selenium-contaminated sediment. *Environmental Science and Technology* **39**: 1771–1777.

**Barberon M, Berthomieu P, Clairotte M, Shibagaki N, Davidian JC, Gosti F**. **2008**. Unequal functional redundancy between the two *Arabidopsis thaliana* high-affinity sulphate transporters SULTR1;1 and SULTR1;2. *New Phytologist* **180**: 608–619.

**Chao DY, Baraniecka P, Danku J, Koprivova A, Lahner B, Luo H, Yakubova E, Dilkes B, Kopriva S, Salt DE**. **2014**. Variation in sulfur and selenium accumulation is controlled by naturally occurring isoforms of the key sulfur assimilation enzyme ADENOSINE 5’-PHOSPHOSULFATE REDUCTASE2 across the arabidopsis species range. *Plant Physiology* **166**: 1593–1608.

**Chen J, Huang XY, Salt DE, Zhao FJ**. **2020**. Mutation in OsCADT1 enhances cadmium tolerance and enriches selenium in rice grain. *New Phytologist* **226**: 838–850.

**Chen Q, Zhu C, Guo L, Bu X, Yang W, Cheng S, Cong X, Xu F**. **2024**. Genome-wide identification of HMT gene family explores BpHMT2 enhancing selenium accumulation and tolerance in Broussonetia papyrifera. *Tree Physiology* **44**.

**Ellis DR, Sors TG, Brunk DG, Albrecht C, Orser C, Lahner B, Wood K V., Harris HH, Pickering IJ, Salt DE**. **2004**. Production of Se-methylselenocysteine in transgenic plants expressing selenocysteine methyltransferase. *BMC Plant Biology* **4**: 1–11.

**Garifullina GF, Owen JD, Lindblom SD, Tufan H, Pilon M, Pilon-Smits EAH**. **2003**. Expression of a mouse selenocysteine lyase in Brassica juncea chloroplasts affects selenium tolerance and accumulation. *Physiologia Plantarum* **118**: 538–544.

**Grant K, Carey NM, Mendoza M, Schulze J, Pilon M, Pilon-Smits EAH, Van Hoewyk D**. **2011**. Adenosine 5′-phosphosulfate reductase (APR2) mutation in *Arabidopsis* implicates glutathione deficiency in selenate toxicity. *Biochemical Journal* **438**: 325–335.

**Van Hoewyk D, Garifullina GF, Ackley AR, Abdel-Ghany SE, Marcus MA, Fakra S, Ishiyama K, Inoue E, Pilon M, Takahashi H, *et al.*** **2005**. Overexpression of AtCpNifS enhances selenium tolerance and accumulation in *Arabidopsis* . *Plant Physiology* **139**: 1518–1528.

**Hugouvieux V, Dutilleul C, Jourdain A, Reynaud F, Lopez V, Bourguignon J**. **2009**. *Arabidopsis* putative selenium-binding protein1 expression is tightly linked to cellular sulfur demand and can reduce sensitivity to stresses requiring glutathione for tolerance. *Plant Physiology* **151**: 768–781.

**Van Huysen T, Abdel-Ghany S, Hale KL, LeDuc D, Terry N, Pilon-Smits EAH**. **2003**. Overexpression of cystathionine-γ-synthase enhances selenium volatilization in *Brassica juncea*. *Planta* **218**: 71–78.

**Van Huysen T, Terry N, Pilon-Smits EAH**. **2004**. Exploring the selenium phytoremediation potential of transgenic Indian mustard overexpressing ATP sulfurylase or cystathionine-γ-synthase. *International Journal of Phytoremediation* **6**: 111–118.

**Jiang L, Chen Z, Gao Q, Ci L, Cao S, Han Y, Wang W**. **2016**. Loss-of-function mutations in the APX1 gene result in enhanced selenium tolerance in *Arabidopsis thaliana*. *Plant Cell and Environment* **39**: 2133–2144.

**Jiang L, Gao QC, Chen ZP, Zhang JJ, Bai XY, He XL, Xu QX**. **2015**. Selenium tolerance of an *Arabidopsis* drought-resistant mutant csm1-1. *Russian Journal of Plant Physiology* **62**: 625–631.

**Jiang L, Yang J, Liu C, Chen Z, Yao Z, Cao S**. **2020**. Overexpression of ethylene response factor ERF96 gene enhances selenium tolerance in *Arabidopsis* . *Plant Physiology and Biochemistry* **149**: 294–300.

**El Kassis E, Cathala N, Rouached H, Fourcroy P, Berthomieu P, Terry N, Davidian JC**. **2007**. Characterization of a selenate-resistant *Arabidopsis* mutant. Root growth as a potential target for selenate toxicity. *Plant Physiology* **143**: 1231–1241.

**Kubachka KM, Meija J, Leduc DL, Terry N, Caruso JA**. **2007**. Selenium volatiles as proxy to the metabolic pathways of selenium in genetically modified Brassica juncea. *Environmental Science and Technology* **41**: 1863–1869.

**Kurmanbayeva A, Bekturova A, Soltabayeva A, Oshanova D, Nurbekova Z, Srivastava S, Tiwari P, Dubey AK, Sagi M**. **2022**. Active O-acetylserine-(thiol) lyase A and B confer improved selenium resistance and degrade l-Cys and l-SeCys in *Arabidopsis* . *Journal of Experimental Botany* **73**: 2525–2539.

**LeDuc DL, AbdelSamie M, Móntes-Bayon M, Wu CP, Reisinger SJ, Terry N**. **2006**. Overexpressing both ATP sulfurylase and selenocysteine methyltransferase enhances selenium phytoremediation traits in Indian mustard. *Environmental Pollution* **144**: 70–76.

**LeDuc DL, Tarun AS, Montes-Bayon M, Meija J, Malit MF, Wu CP, AbdelSamie M, Chiang CY, Tagmount A, DeSouza M, *et al.*** **2004**. Overexpression of selenocysteine methyltransferase in *Arabidopsis* and Indian mustard increases selenium tolerance and accumulation. *Plant Physiology* **135**: 377–383.

**Malkowski MG, Quartley E, Friedman AE, Babulski J, Kon Y, Wolfley J, Said M, Luft JR, Phizicky EM, DeTitta GT, *et al.*** **2007**. Blocking S-adenosylmethionine synthesis in yeast allows selenomethionine incorporation and multiwavelength anomalous dispersion phasing. *Proceedings of the National Academy of Sciences of the United States of America* **104**: 6678–6683.

**Matich AJ, McKenzie MJ, Brummell DA, Rowan DD**. **2009**. Organoselenides from Nicotiana tabacum genetically modified to accumulate selenium. *Phytochemistry* **70**: 1098–1106.

**McKenzie MJ, Hunter DA, Pathirana R, Watson LM, Joyce NI, Matich AJ, Rowan DD, Brummell DA**. **2009**. Accumulation of an organic anticancer selenium compound in a transgenic Solanaceous species shows wider applicability of the selenocysteine methyltransferase transgene from selenium hyperaccumulators. *Transgenic Research* **18**: 407–424.

**Ohno M, Uraji M, Shimoishi Y, Mori IC, Nakamura Y, Murata Y**. **2012**. Mechanisms of the selenium tolerance of the *Arabidopsis thaliana* knockout mutant of sulfate transporter SULTR1;2. *Bioscience, Biotechnology and Biochemistry* **76**: 993–998.

**Pilon M, Owen JD, Garifullina GF, Kurihara T, Mihara H, Esaki N, Pilon-Smits EAH**. **2003**. Enhanced selenium tolerance and accumulation in transgenic *Arabidopsis* expressing a mouse selenocysteine lyase. *Plant Physiology* **131**: 1250–1257.

**Pilon-Smits EAHH, Hwang S, Lytle CM, Zhu Y, Tai JC, Bravo RC, Chen Y, Leustek T, Terry N**. **1999**. Overexpression of ATP sulfurylase in Indian mustard leads to increased selenate uptake, reduction, and tolerance. *Plant Physiology* **119**: 123–132.

**Sabbagh M, Van Hoewyk D**. **2012**. Malformed selenoproteins are removed by the ubiquitin-proteasome pathway in *Stanleya pinnata*. *Plant and Cell Physiology* **53**: 555–564.

**Song Z, Shao H, Huang H, Shen Y, Wang L, Wu F, Han D, Song J, Jia H**. **2017**. Overexpression of the phosphate transporter gene OsPT8 improves the Pi and selenium contents in *Nicotiana tabacum*. *Environmental and Experimental Botany* **137**: 158–165.

**Sors TG, Ellis DR, Gun NN, Lahner B, Lee S, Leustek T, Pickering IJ, Salt DE**. **2005**. Analysis of sulfur and selenium assimilation in *Astragalus* plants with varying capacities to accumulate selenium. *Plant Journal* **42**: 785–797.

**Sun SK, Xu X, Tang Z, Tang Z, Huang XY, Wirtz M, Hell R, Zhao FJ**. **2021**. A molecular switch in sulfur metabolism to reduce arsenic and enrich selenium in rice grain. *Nature Communications* **12**.

**Tagmount A, Berken A, Terry N**. **2002**. An essential role of S-adenosyl-L-methionine:L-methionine S-methyltransferase in selenium volatilization by plants. Methylation of selenomethionine to selenium-methyl-L-selenium-methionine, the precursor of volatile selenium. *Plant Physiology* **130**: 847–856.

**Wu X, Tao M, Meng Y, Zhu X, Qian L, Shah A, Wang W, Cao S**. **2020**. The role of WRKY47 gene in regulating selenium tolerance in *Arabidopsis thaliana*. *Plant Biotechnology Reports* **14**: 121–129.

**Zhang L, Hu B, Deng K, Gao X, Sun G, Zhang Z, Li P, Wang W, Li H, Zhang Z, *et al.*** **2019**. NRT1.1B improves selenium concentrations in rice grains by facilitating selenomethinone translocation. *Plant Biotechnology Journal* **17**: 1058–1068.

**Zhang L, Hu B, Li W, Che R, Deng K, Li H, Yu F, Ling H, Li Y, Chu C**. **2014**. OsPT2, a phosphate transporter, is involved in the active uptake of selenite in rice. *New Phytologist* **201**: 1183–1191.

**Zhao XQ, Mitani N, Yamaji N, Shen RF, Ma JF**. **2010**. Involvement of silicon influx transporter OsNIP2;1 in selenite uptake in rice. *Plant Physiology* **153**: 1871–1877.

**Zhou X, Yuan Y, Yang Y, Rutzke M, Thannhauser TW, Kochian L V., Li L**. **2009**. Involvement of a broccoli COQ5 methyltransferase in the production of volatile selenium compounds. *Plant Physiology* **151**: 528–540.
