# Supplementary material for: An atlas of plant selenium metabolism
Source: New Phytol. 2026 Mar 16;250(4):2041–60. doi: 10.1111/nph.71087 (PMC13103439; doi:10.1111/nph.71087)
Supplement: Supplementary file 5 — Table S4 Table summarizing 83 transcriptomics, metabolomics, proteomics and genomics studies on plant Se metabolism. [file NPH-250-2041-s004.docx]

**New Phytologist Supporting Information**

**Article title:** “An Atlas of plant selenium metabolism”

**Authors:** Jeroen van der Woude, Mark G. M. Aarts, Michela Schiavon & Antony van der Ent

**Article acceptance date:** 14 February 2026

**Table S4.** List of (multi) -omics, genome-wide and integrated studies of the plant Se metabolism, including untargeted metabolomics, untargeted proteomics, transcriptomics, genomics and genotype screening (QTL analysis or GWAS) studies on plants exposed to Se and/or Se accumulators. For each reference, the type of method, treatment conditions, plant species and main findings are summarized. From these studies, 67 out of 83 are from within the last 10 years, providing an extended overview of more recent genome-scale research efforts into plant Se metabolism.

| **Type of study** | **Plant species** | **Treatment conditions** | **Results of note** | **Source** |
| --- | --- | --- | --- | --- |
| Transcriptome (microarray) | *Arabidopsis thaliana* (Col-0 and overexpressed *CpNifS*) | 10d on vertical ½ MS agar medium with or without 40 𝞵M Na_2_SeO4 | Overexpression of CpNifS resulted in increased S and Se levels, increased tolerance to SeO_4_ and reduced incorporation of Se into proteins. The response of *A. thaliana* to Se mimicked a S deficiency response. | (Van Hoewyk *et al.*, 2005) |
| QTL analysis | *Arabidopsis thaliana* (Ler-0 x Col-4) | 10d on vertical ½ MS agar medium with or without 30, 40 or 50 𝞵M Na_2_SeO4 | Three quantitative trait loci (QTL) were identified for selenate tolerance on chromosomes 1, 3 and 5, together explaining 24% of the tolerance phenotype. Potential genes of interest in these QTLs were SMT, APS1, SBP3, SAT1 and CGS. | (Zhang *et al.*, 2006b) |
| Bulked segregant analysis | *Arabidopsis thaliana* (Ws, Col, Ler) | 10d on vertical ½ MS agar medium with or without 50 𝞵M Na_2_SeO4 or 20 𝞵M Na_2_SeO_3_ | Ler was significantly more sensitive to selenate, while Ws was more sensitive to selenite. Furthermore, three molecular markers were associated with selenate tolerance, while only one was associated with selenite tolerance. Selenite and selenate tolerance appear to be (partially) independent and correlate with SeCystine and SeCysteine levels. | (Zhang *et al.*, 2006a) |
| Genotype study | *Arabidopsis thaliana* (19 accessions) | 10d on vertical ½ MS agar medium with or without 50 𝞵M Na_2_SeO4 or 20 𝞵M Na_2_SeO_3_ | Selenate was more toxic than selenite, and uptake of Se was higher in selenate-treated plants. A strong correlation between Se and S content existed in roots (selenite and selenate treated plants) and shoots (selenate treated plants). There was no correlation between Se tolerance and accumulation. | (Zhang *et al.*, 2007) |
| Transcriptome | *Saccharomyces cerevisiae*  (baker’s yeast) | Rich medium (YPD) at 30 ºC, with or without addition of 1 mM Na_2_SeO_3_, 0.3 mM H_2_O_2_ or 20𝞵g/ml benomyl | Selenite activated the yeast iron deprivation, oxidative stress and protein degradation responses. Proteasome regulators and oxidative stress response regulators were involved in the selenite detoxification response. Cell growth gene networks were repressed by selenite. | (Salin *et al.*, 2008) |
| **Transcriptome**  **(microarray)** | ***Arabidopsis thaliana* (Col-0 and Ws-2)** | **7d on vertical ½ MS agar medium with or without 15 𝞵M Na_2_SeO_3_** | **Selenite-tolerant Col-0 had higher levels of ethylene and JA, while Ws-2 had lower levels of these phytohormones and lower responses of ethylene/JA induced genes. Addition of these phytohormones increased Ws-2 selenite tolerance, and tolerance of Col-0 ethylene and JA signalling or biosynthesis mutants was decreased. Col-0 showed higher levels of S transport and assimilation genes, and higher tissue S and Se levels.** | (Tamaoki *et al.*, 2008) |
| Transcriptome (microarray) | *﻿Solanum tuberosum* (potato) | 5 weeks at 22ºC natural light, foliar sprayed with 0.75 mM Na_2_SeO_3_ solution. | A general repression of photosynthesis-related genes occurs, maximizing at 8h post treatment. After 24h, the levels restore. Genes related to amino acid metabolism and stress defence were activated by SeO_3_, especially glutathione S-transferases. Authors suggest Se interferes with Fd-TRx system, known to be involved in photosynthesis regulation. | (Poggi *et al.*, 2008) |
| **Transcriptome**  **(microarray)** | ***Arabidopsis thaliana* (Ws-1)** | **10d on vertical ½ MS agar medium with or without 40 𝞵M Na_2_SeO_4_** | **Roots and shoots showed distinct transcriptional responses to SeO_4_, and many S assimilation & transport genes were upregulated. Transcripts of ethylene and JA signalling were upregulated by Se, and mutants of these signalling networks also showed lower tolerance. SeO_4_ activated some genes associated with salt and osmotic stress. Cell development was repressed by Se, such as cell wall and auxin-regulated genes.** | (Van Hoewyk *et al.*, 2008) |
| Transcriptome (macroarray) | *Stanleya pinnata* & *Stanleya albescens* | 10 weeks growth with 0 or 20 𝞵M Na_2_SeO_4_ | Se accumulation by Se-tolerant *S. pinnata* was 3.6-fold higher than in Se-sensitive *S. albescens*. Ascorbic acid, GSH, S and thiol levels were higher in *S. pinnata*, SMT protein levels and SeMeCys fraction of total Se were higher too. *S. pinnata* showed higher expression of genes in S assimilation, antioxidant, defence and JA, SA and ethylene signalling. Levels of MeJA and SA were constitutively higher in *S. pinnata*. | (Freeman *et al.*, 2010) |
| Transcriptome (RNA sequencing) | *Lolium perenne*  (perennial ryegrass) | 25L hydroponics, with or without 100 𝞵M Na_2_SeO_4_ | Several ATP binding cassette (ABC) transporters were identified as significantly upregulated by the Se treatment, of which some were putatively associated with transport of phytic acid, the thylakoid membrane and a multidrug resistance-associated protein. In addition, a glutathione peroxidase, a glutathione reductase and selenium-binding protein were upregulated. A sulfate transporter (homolog of SULTR1;2) was downregulated by Se treatment. | (Byrne *et al.*, 2010) |
| Transcriptome and metabolome | *Arabidopsis thaliana* | Seedlings were grown in 0.5 L hydroponics with ¼ Hoagland’s nutrient solution. 14d old seedlings were exposed to 0 or 10 𝞵M Na_2_SeO_4_ for 14 days, after which shoots were harvested. | Se treatment reduced the dry shoot weight significantly by -48%, indicating that even 10 𝞵M selenate was toxic to *A. thaliana*. Various genes involved in glucosinolate (GSL) biosynthesis were downregulated by Se treatment, including genes in aromatic-, indolic- and aliphatic glucosinolate biosynthesis. This was confirmed by the measured GSL levels in the shoot, which were significantly lower. The levels of chlorophyll and carotenoids also trended towards reduction, but this was not statistically significant, except for the carotenoid lutein. This study supports the general trend that Se exposure causes a S deficiency-like response, and that Se toxicity results in reduced pigments and photosynthesis. | (Sams *et al.*, 2011) |
| Transcriptome  (Fluorescent differential display) | *Astragalus racemosus* | 2 weeks with or without 20 𝞵M K_2_SeO_4_ or 20 𝞵M K_2_SeO_3_ | The K_2_SeO_4_ treatment resulted in differential expression of 125 genes, of which nine genes were change 2-fold; 14 genes showed a 2-fold difference in the K_2_SeO_3_ treatment. Among the genes both affected by selenite and selenate, there was an upregulated rubisco subunit, a downregulated Zn-binding quinine reductase (alkenyl/one oxidoreductase) and some proteins without a hit (even to this day). | (Hung *et al.*, 2012) |
| Metabolomics | *Bertholletia excelsa*  (brazil nut) & *Lecythis minor* (monkeypot nuts) | Commercially available samples | The Se concentration of the secondary accumulator (*B. excelsa*) was 127 𝞵g Se/g, and that of the hyperaccumulator (*L. minor*) was 4480 𝞵g Se/g. Several new Se compounds were identified, including some hydrophobic (terpene) derivatives of SeHCys and polyselenides with >2 Se atoms per molecule. The latter are hypothesized to form due to an excess of SeHCys and H_2_Se, perhaps forming spontaneously. | (Németh *et al.*, 2013) |
| Genotype screening | *Arabidopsis thaliana*  (349 accessions) | 44-day growth in common garden with 0.1x Hoagland’s | By screening 349 accessions for S and Se uptake, two variants of the same gene (APR2) were identified with reduced catalytic efficiency and lowered conversion of sulfate to organic sulfur molecules. Therefore, these accessions contained significantly more sulfate and selenate in the leaves. | (Chao *et al.*, 2014) |
| Metabolomics | *Brassica napus* | 4L Hydroponics in Hoagland’s medium for 3 weeks, then 1 week with/ without 50 𝞵M Na_2_SeO_3_. | Selenite treatment triggered formation of mitochondrial superoxide and activated the alternative oxidase pathway. Pyruvate and oxoglutarate concentrations were much increased by SeO_3_, both of which are substrates for enzyme complexes (oxoglutarate dehydrogenase and pyruvate decarboxylase) with three S-containing cofactors (lipoic acid, thiamine and coenzyme A). Se treatment also increased GSH content and activity of GGCT, possibly involved in GSH-mediated Se detoxification. Finally, Se increased amino acid content, showing large-scale metabolic adjustments to Se stress in the roots of *B. napus*. | (Dimkovikj & Van Hoewyk, 2014) |
| **Transcriptome** | ***Astragalus chrysochlorus*** | **0 and 26 𝞵M SeO_4_ treatment of callus cultures** | **The Se treatment had strong effects on ABC transporters, plant pathogen interactions, secondary metabolism and (mitochondrial) carbohydrate metabolism. Furthermore, Se activated 26S proteasome regulatory particles, heat shock proteins and 1-aminocyclopropane-1-carboxylate oxidase (involved in ethylene biosynthesis).** | (Cakr *et al.*, 2015) |
| Transcriptome | *Oryza sativa* (rice) | Seed with or without priming: 60 𝞵M Se* or 100 mg/L salicylic acid (SA) and 7-day submersion in water  ^*(chemical form not recorded)^ | Se and SA priming increased germination, shoot- and root length and fresh weight of rice seedlings. Compared to non-primed, the Se primed submerged plants had higher expression of genes involved in (among others) protein modification/ phosphorylation, response to drugs/ chemical stimuli, carbohydrate and phosphate metabolic processes and transmembrane transport. Downregulated processes were (among others) lipid transport, response to stress, signal transmission, carboxylic acid/ ketone metabolic processes. | (Hussain *et al.*, 2016) |
| Ionomics, metabolomics, RT-QPCR | *Arabidopsis thaliana* | 35-day growth on soil, after 2 weeks treated with 10 ml water or 250 𝞵M Na_2_SeO_4_ solution | Se-treated *A. thaliana* plants were roughly half the size and weight of control yet had similar specific leaf area (weight/area), water content, root: shoot ratio and leaf number. Se also lowered levels of sugar and most amino acids, while increasing concentrations of nitrate, GABA, proline and homoserine. Finally, SeO_4_ treated *A. thaliana* had lower P, Ca and Zn but higher S and Se levels in both root and shoot. | (Ribeiro *et al.*, 2016) |
| QTL mapping | *Triticum aestivum* (131 recombinant inbred lines RILs) | Hydroponic culture, modified Hoagland for wheat, with or without 0.1 𝞵M Na_2_SeO_3_ | The uptake efficiency of P, K, Ca, Mg, Fe, Mn, Cu and Zn was measured with or without Se. 260 QTLs were identified in total, of which 190 were found in cooperative uptake & utilization (CUU) loci, which affected at least two elements. | (Gong *et al.*, 2016) |
| Metabolomics | *Helianthus annuus* (sunflower sprouts), *Raphanus sativus* (radish sprouts), *Allium sativum* (garlic) | Germinated with or without 40 (sunflower) or 20 mg/L (radish) Na_2_SeO_3_, or grown in soil irrigated with 350 mg/L Na_2_SeO_3_ (garlic) | Total Se concentrations were 73.6 (sunflower), 156 (radish) and 182 mg Se/kg (garlic). SeMeCys and SeMet (and their derivatives) were found in all plants, and ﻿2,3-Dihydroxypropionyl-selenocysteine-cysteine was identified in both sunflower and radish. Selenoglutathione and derivatives were also identified in sunflower and garlic, with the latter also containing selenosugars and selenohomolanthionine. The wide phylogenetic distribution of SeMeCys is peculiar, and indeed this compound is not unique to Se hyperaccumulators. | (Ruszczyńska *et al.*, 2017) |
| **Transcriptome** | ***Stanleya pinnata* & *Stanleya elata*** | **Half-strength MS medium with or without 20 𝞵M Na_2_SeO_4_ for 30 days.** | **Various genes involved in S transport and assimilation were constitutively overexpressed in *S. pinnata* compared to *S. elata*, including SULTR3;1 (shoot), SULTR3;4, SULTR1;2 (root), GSH1/ ECS and most notably an extremely high expression of APS2 in the roots. Genes related to defence mechanisms and jasmonic acid, ethylene and salicylic acid were also elevated in *S. pinnata,* among which APX1, TPX, GPX, JAR1 and EIN3. The authors suggest Se hyperaccumulation of *S. pinnata* relies on constitutive upregulation of JA, SA and ethylene-regulated (antioxidant) defence mechanisms and sulphur assimilation and transport genes.** | (Wang *et al.*, 2018) |
| GWAS, Ionomics | *Glycine max*  (1653 lines) | Field grown | Large ionomic screening of multiple elements in *G. max* on different fields.  SNPs associated with Se levels indicated candidate genes from the **NRAMP** (aluminium sensitive 3, iron and manganese transporter), **ABC transporte**r and **sulphate transporter** families. The sulfate transporter is not that surprising, yet the link between iron transporters and ABC transporters with Se levels is more novel. | (Ziegler *et al.*, 2018) |
| **Transcriptome** | ***Cardamine hupingshanensis*** | **5-month-old seedlings, washed, placed in 0.1 L Hoagland solution for 24 hours with or without 100 or 80000 𝞵g Na_2_SeO_3_** | **The SeO_3_ treatment of *C. hupingshanensis* resulted in upregulation of root sulfite oxidase, 2 ABC transporters (one of which ABCC involved in transport of glutathione conjugates), a GST, Grx3 and Trx1, multiple peroxidases various sulfur transport/assimilation genes (APS, APR, CGS, CBL, MET, SOT1) and genes involved in the ubiquitin-proteasome pathway (E2 and E3 ubiquitin ligases). Among the downregulated genes were rubisco subunits, photosystems, peroxidases, and SULTR1;2.** | (Zhou *et al.*, 2018) |
| Transcriptome | *Camellia sinensis* (tea) | 10 L hydroponics with 20 seedlings, modified Hoagland (pH 5.0) with or without 50 𝞵M of Na_2_SeO_3_ for one month | Se concentrations in Na_2_SeO_3_ exposed tea were ~1.3 mg Se/kg *fresh* leaves (4.67 𝞵g Se/g dry leaf) and ~75 mg Se/kg *fresh* roots. In both leaves and roots, Se affected oxidative phosphorylation, **thiamine metabolism**, **sulfur metabolism** (taurine, sulfur relay, cysteine methionine) and fatty acid metabolism, while Se affected leaf **terpenoid- carotenoid and phenylpropanoid metabolism** and Se affected root **glutathione metabolism**, ribosomes, phagosomes, and **oxidative stress responses (peroxisomes, ascorbate**). Various phosphate transporters were overexpressed due to Se treatment, and some genes involved in ethylene- and JA signalling were responsive to Se too. | (Cao *et al.*, 2019) |
| Transcriptome | *Brassica oleracea* var. *alboglabra* (Chinese kale) line ﻿BOK92 | Seedlings in sand, with nutrient solutions with or without Na_2_SeO_3_ (20, 40, 60, 80, 100, 120 𝞵M), or various concentrations of NaCl, MeJA or brassinolide for 7 days in tissue culture room | Selenite treatment resulted in similar total glucosinolate levels as control, but with increased content of sulphurophane (glucosinolate breakdown product) with increasing Se treatment. The Se and MeJA treatments showed the least distance in clustering analyses between all five treatments. Se and MeJA notably increased the expression of methionine synthase. This study gives an interesting insight in the overlap of selenite and MeJA responses. | (Wu *et al.*, 2019b) |
| Transcriptome | *Aegilops tauschii*  (wheat ancestor; two genotypes) | 54 days old seedling in hydroponics, treated with or without 10 𝞵M Na_2_SeO_3_ for 3 days | Two genotypes with contrasting Se accumulation (highest and lowest) were compared in terms of transcriptional response to Se. The Se response of the **high Se genotype** showed enrichment of **ascorbate, tyrosine, beta-alanine metabolic pathways**, among others, while the difference between the high- and low Se genotypes was characterized by **differences in glutathione metabolism, plant-pathogen interactions, fructose and mannose metabolism, phenylpropanoid biosynthesis and aminoacyl-tRNA biosynthesis**. Finally, important processes enriched both between genotypes and in high Se genotype Se response were **flavonoid biosynthesis and cutin- suberin and wax biosynthesis.** | (Wu *et al.*, 2019a) |
| Transcriptome and metabolome | *Ginkgo biloba* | Two-year-old cuttings were subjected to 10 mg/ml Na_2_SeO_3_ sprayed on leaves or irrigated on roots, then immediately harvested. | Foliar-applied selenite affected photosynthetic antenna proteins, flavanol biosynthesis, fatty acid elongation, cutin- suberin- and wax biosynthesis and anthocyanin biosynthesis. Root-applied selenite affected photosynthetic antenna proteins, cutin- suberin- and wax biosynthesis, flavanol biosynthesis, fatty acid biosynthesis and -elongation and biotin metabolism. | (Li *et al.*, 2019) |
| Proteomics | *Oryza sativa* (rice, two cultivars, one bred for Se enrichment) | Provided by Rice Testing Centre of the Ministry of Agriculture, treatment not recorded. | In the Se-enriched cultivar, 401 out of 3161 quantified proteins were differentially expressed (208 down, 193 upregulated). In Se-enriched rice, proteins involved in **stress response, antioxidant**, carbohydrate and amino acid metabolism were differentially regulated. **Cysteine synthase and methionine synthase** are hypothesized as key proteins that cause amino acid differences, while APX2, CAT, HSP70 and HSP90 (among others) were associated with stress responses, which were confirmed by RT-qPCR. | (Zeng *et al.*, 2019) |
| Proteomics | *Capsicum annuum* | Pepper plants grown in greenhouse, irrigated with ½ Hoagland’s, treated at four leaf stage with ½ Hoagland’s with or without 100 ppm Na_2_SeO_4_ for 24 hours, shoots harvested. | The leaves of Se-treated pepper plants contained a higher abundance of proteins related to the extracellular region, cell wall & cell wall macromolecules (glucan, polysaccharide, glycosamide), iron ion binding, prephenate dehydrogenase activity and vesicular transport and lower abundance of proteins related to water soluble vitamins (thiamine specifically) and porphyrin biosynthesis (chlorophyll specifically). In addition, various ethylene biosynthesis genes were significantly upregulated by Se, as were heat shock proteins and chaperones. | (Zhang *et al.*, 2019a) |
| Transcriptome & metabolome | *Brassica rapa.* Var. *chinensis* (Pak choi) | 2.5L Hydroponic culture, 1/5x Hoagland’s. After 21 days treated with or without 10 𝞵mol Na_2_SeO_3_ or Na_2_SeO_4_ for 1, 3 or 7 days. | Longer Se treatments of both forms led to higher tissue Se levels, with **selenate treated plants having much higher shoot Se levels (~**43 vs. ~3 mg Se/kg fresh shoot after 7d) and significantly higher root Se levels compared to selenite treated plants (~33 vs. ~21 mg Se/kg fresh root after 7d). Selenite was only detected in roots in low levels (0.22 – 26%), but selenate was detected in much higher fractions in both root (2.6-76% of total Se) and shoot (24-67%), even in selenite treated plants. Overall, SeMet and SeO_4_ were the major forms of Se, with smaller fractions of SeMeCys and SeCys_2_. SULTR1;1 and SULTR1;2 expression was increased by selenate, while PHT1;2 and PHT1;4 expression in roots was increased by selenite treatment. | (Yu *et al.*, 2019) |
| Transcriptome & metabolome | *Apium graveolens* (celery) | Hydroponics in Hoagland’s solution, 6-week-old plants submitted to 0 or 100 ppm Na_2_SeO_4_ for 48 hours. | Significantly affected pathways in **Se-treated celery leaves** were (among others) **proteasome**, **shikimate pathway processes** (phenylpropanoid-, phenylalanine, tyrosine biosynthesis), pentose phosphate pathway/ carbon fixation/ gluconeogenesis, starch & sucrose metabolism, glycerolipid metabolism, TCA cycle, glutathione metabolism and alpha linolenic acid metabolism. Various sulfur-related genes were also affected, including upregulation of methionine synthase, APS3, APR3, SAT1, SIR, MGL CGS, and a MET17-like homocysteine synthase (synthesizing methionine from acetyl-homoserine and methanethiol). In addition, several phosphate- and sulfate transporter genes (SULTR 1.3; -3.5 and -4.1) were activated by Se. | (Zhang *et al.*, 2019b) |
| Metabolomics | *Cucumis sativus*  (cucumber) | Hydroponically grown 7-day old seedlings were treated for seven days with basal nutrient solution or without 3𝞵M Na_2_SeO_3_ and/or 50 𝞵M CdCl_2_  (four treatments) | Se protected the Cd-induced damage to ultrastructures in roots and leaves of cucumber, including restoration of chloroplast morphology and chromatin distribution in the nuclei. Several metabolites that were depleted in Cd treatment were restored in the Se+Cd treatment, among which glycine (root & leaf), alanine (leaf) and phytol (leaf), homoserine (root) and lactic acid (root). Other metabolites were enriched in Cd treatment but repressed in Cd+Se treatment, such as fructose (root), succinate (root), shikimate (root), oxo proline (root) and sulfate (root). This showcases large metabolomic effects resulting from a very mild Se supplementation. | (Sun *et al.*, 2020) |
| Transcriptome | *Pueraria lobata*  (kudzu vine) | Cuttings from 1 year old plant, grown in soil in greenhouse. Seedlings with 5 leaves were treated with 200 ml of 0, 1, 5, 15, 25, 35 or 55 mg/L Na_2_SeO_3_ solution. Of these, the roots of the 25 mg/L treatment were used for transcriptome analysis. | Selenite treatments in excess of 25 mg/L significantly increased the level of malondialdehyde (as a proxy measurement of lipid peroxidation). Treatment of *P. lobata* roots with selenite affected genes involved in transcriptional regulation, response to biotic stresses, response to acid chemicals/ lipids, response to hormones (including ABA), response to water deprivation and flavonoid biosynthetic process. | (Guo *et al.*, 2020) |
| Transcriptome | *Cardamine violifolia* | 10L hydroponic tanks with first 10d ¼ Hoagland’s, then ½ Hoagland’s with or without 0.25, 4 or 16 mg/L Na_2_SeO_4_ for 30 days. | The highest Se treatment (16 mg/L) resulted in ~10000 mg Se/kg dry plant, with the majority of this (~8500 mg Se/kg) in organic Se form, but the biomass of these plants was ~3-fold lower than control, the chlorophyll content halved and vitamin C levels more than doubled. The 0.25 mg/L treatment increased growth by ~40%, suggesting a hormetic response. Compared to control, the Se treatments significantly affected genes in plant hormone signalling, linoleic acid metabolism, amino- and nucleotide sugar metabolism, and plant pathogen metabolism (among others). | (Rao *et al.*, 2020) |
| Metabolome | *Cardamine violifolia* | Leaves were taken from the Wuhan Botanical Garden | The total Se content in the sample was 261 mg Se/kg dry leaf. In-depth investigation of the selenometabolome indicated presence of 13 compounds that contain the selenohomocysteine moiety, among which selenocystathionine and various others. In addition, various aliphatic Selenocompounds were identified as well as selenosugars. The authors suggest that the variety of selenosugars could serve as selenium depositories to alleviate Se toxicity in the hyperaccumulator. | (Ouerdane *et al.*, 2020) |
| Proteomics | *Camellia sinensis* | 18 batches of tea were bought from markets, six of which were Se-enriched. | Compared to regular tea, the Se-enriched tea samples contain higher levels of dehydrins, methionine synthase, MDHR, GR2, SAMS, SULTR2;1, ICE1, SOD, APX and ADH (among others), and lower levels of MT, AOX, SAMS, RBOHA, SPDS, APX, SULTR4;2, SOD and ACCO (among others). GO and KEGG terms significantly enriched in the affected proteins were photosynthesis (photosystem I&II, chloroplast membrane), oxidative phosphorylation (ATP synthase), amino acid metabolism (glutamate decarboxylase, AA transmembrane transport, cysteine biosynthesis), JA responses, glutathione metabolism and metal ion binding (magnesium, iron, Fe_4_S_4_ clusters) | (Jia *et al.*, 2020) |
| Genomics | 137 species of algae | Genomic- and expressed sequence tag (EST) files were downloaded from Entrez Genome Project | The selenoproteomes (proteins with encoded selenocysteine) of a large variety of microalgae is mapped out. This resulted in more 1000 identified selenoproteins in 42 selenoprotein families, of which three new selenoprotein families. The abundance of true selenoproteins follows roughly among phylogenetic lines, albeit in a mosaic pattern. **Another important determining factor is the habitat of the algae, with seawater algae more often sporting large selenoproteomes compared to freshwater and dry land algae.** | (Jiang *et al.*, 2020) |
| Transcriptomics | *Oryza sativa* | Field experiment in Mozambique, treated with or without 900 g/ha ZnSO_4_ and/or 500g/ha Na_2_SeO_3_, flag leaves sampled 15d after treatment for transcriptome | Zn-treated rice plants showed increased expression of genes involved in nitrogen/ amino acid metabolism, RNA degradation, photosystem I and the thiamine-requiring oxoglutarate dehydrogenase complex (2-oxoglutarate 🡪 succinyl-CoA), involved in the TCA cycle. Se treatment of rice significantly affected genes related to thiamine and biotin metabolism. Combined treatments affected cytoplasmic vesicles, ubiquitin ligase complexes, lipid localization& transport, cofactor& coenzyme binding. | (Roda *et al.*, 2020) |
| Transcriptome | *Puccinellia distans* (weeping alkali grass) | 7w old seedlings placed 1w in 5L hydroponics in 1x Hoagland’s, then treated with or without 20, 40, 80 or 120 𝞵M Na_2_SeO_4_ for 1w | *P. distans* is a boron hyperaccumulator yet was able to accumulate 250 mg Se/kg dry shoot at the highest treatment, without too much toxicity (mild chlorosis). Se treatment increased expression of 7 heat shock protein/ chaperone genes, a peroxidase gene, genes involved in sulfate assimilation (APS and SIR), NADPH-dependent thioredoxin reductases (NTR) and a glutathione conjugate ABC transporter (ABCC) (among others). Se treatment also increased enzymatic activity of glutathione reductase. Se treatment decreased expression of various ribosomal proteins, mitochondrial NADH dehydrogenases, phosphate transporter 2, cysteine synthase and SULTR4;1. Finally, genes involved in jasmonic acid synthesis are induced by Se treatment. | (Kök *et al.*, 2020) |
| Transcriptome | *Capsicum annuum* | 17d old seedling were treated with or without 0.2 or 1 mg/L nano-Se and/or 1 mg/L Cd^2+^ for 7 days, sampling root, stem and leaf | Cd-treatment significantly reduced biomass of root, stem and leaf compared to untreated pepper plants, and nano-Se partially restored the biomass values. Nano-Se activated LOX3 (involved in JA biosynthesis), and increased JA levels. Treatment with 0.2 mg/L Se significantly affected photosynthesis, chlorophyll production, carbon fixation, sucrose, galactose& starch metabolism, ascorbate metabolism, nitrogen metabolism, glycosphingolipids metabolism, plant hormone signalling and secondary metabolism (isoflavonoids, anthocyanin) | (Li *et al.*, 2021) |
| Transcriptome and metabolome | *Brassica oleracea* var. *italica*  (broccoli) | Grown in 3.5 kg soil, with vegetable nutrients, 6.58 mg Se/kg dry soil. After 30 days, plants were treated with 300 ml of 0, 0.1, 0.2, 0.4, 0.8 or 1.6 mM Na_2_SeO_4_ for four times during 60 days. | The 0- and 0.4-mM treatments were used for transcriptome and metabolome analyses, since these plants grew well, containing around 250 mg Se/kg dry plant. At the highest treatment, the broccoli contained around 1600 mg Se/kg dry plant and contained an increased S level of ~2050 mg S/kg compared to control (~1550 mg S/kg). The 0.4 mM treatment appears as a boundary treatment, with higher Se levels decreasing the biomass and S levels while lower treatments did not achieve this. Furthermore, the 0.4 mM treatment resulted in a dramatic increase in glucosinolate content and lower flavonoid content compared to all other treatments. 0.4 mM selenate significantly affected genes involved in (among others) nitrogen and almost all amino acid metabolic processes (tyrosine, histidine, lysine, glycine, serine, threonine, arginine, alanine, aspartate, glutamate, proline, phenylalanine, cysteine, methionine, tryptophan), peroxisomes, glycerolipid- & fatty acid metabolism, cutin- suberin- and wax biosynthesis and phenylpropanoid-, stilbenoid- diarylheptanoid- and gingerol biosynthesis and taurine- and hypotaurine metabolism. Metabolites affected by 0.4 mM selenate were (among others) flavonoids, glucosinolates, phenolic acids, uracil and (R)-pantetheine (precursor of coenzyme A) | (Rao *et al.*, 2021a) |
| Transcriptome and metabolome | *Aloe vera* | Soil grown plants sprayed with 0, 200- and 400 mg Na_2_SeO_4_ /L at a 15-day interval for 3 months. | Both Se treatments increased Se content from ~60 mg Se/kg dry plant to ~400 mg Se/kg. Se treatments activated antioxidant metabolism as seen from gene transcripts and metabolites and activated S assimilation pathways and sulfate transporters. Phytohormone signalling and transcription factors were activated too, as were a whole suite of secondary metabolites including phenols, flavonoids, terpene- and indole derivatives. The heatmaps clearly show that Se alternatively increased- and decreased the levels of various chemical species within a class, e.g. reducing the levels of one flavonoid compound while increasing another. This highly specific metabolic remodelling warrants further investigation. | (Zou *et al.*, 2021) |
| **Genome, epigenome, transcriptome, metabolome** | ***Cardamine enshiensis*** | **For transcriptome and metabolome, two-month-old plants were treated with or without 400 𝞵M Na_2_SeO_4_ for 24 hours. The second RNA extraction was done on 2-week treatments.** | **This extensive study resulted in the chromosome-level (2n =32) assembly of the genome of Se hyperaccumulator *C. enshiensis*, the first of the kind. Additionally, metabolome and transcriptome analyses reveal an important role for flavonoids, glutathione and lignin biosynthesis in the Se response of *C. enshiensis*. Hi-C chromatin analysis suggests that Se treatment affects the compartmentalization of the genome, potentially driving the transcriptional response to Se by topologically associated domains and chromatin modifications. The transcriptional response of the S assimilatory pathway of *C. enshiensis* to Se can be characterized by an increased activity of SULTR, CGS, SiR, OAS-TL, GSH, MS and MMT expression in the roots, decreased activity of APK and CGS and increased APR activity in the leaves (e.g. overall increased S metabolism in the roots, increased cysteine synthesis and lower sulfated compound biosynthesis, such as glucosinolates, in the leaves). In addition, Se treatment shifts around the expression pattern of glutathione-S transferases and GSH peroxidases, with some reducing- and other increasing in activity. Overall, *C. enshiensis* appears to have advanced methods of regulating the transcriptome in response to Se exposure, including sophisticated rebalancing of the S metabolic pathways.** | (Huang *et al.*, 2021) |
| Transcriptome, proteome, metabolome | *Cardamine violifolia* | 10 L hydroponics tank with 11 plants/tank, ¼ Hoagland’s solution for 10d, then ½ Hoagland’s with 0, 0.25, 4.0 or 16.0 mg Na_2_SeO_4_ /L for 30 days. | The 0.25 mg selenate /L treatment increased total fresh weight compared to control (from ~7.4 g to ~10.2g) at 120.3 mg Se/kg dry tissue, while the 16 mg selenate/L treatment decreased total fresh weight (down to ~2.7g) at 9955 mg Se/kg dry tissue.  In response to Se treatment, various S assimilatory genes were activated, including SULTR1;1, -1;2 and -2;1, APR1, APR3 as well as SBP1 and SDI2. The proteomic analysis indicated a significant effect of Se treatment on proteins involved in (among others) endocytosis, peroxisomes, endoplasmic reticulum and ribosomes, phagosome & proteasome, amino acid metabolism (Ala, Asp, Glu, Cys, Met, Gly, Ser, Thr, Phe, Tyr and Trp), major carbon cycles (TCA cycle, gluconeogenesis, pentose phosphate pathway, carbon fixation), oxidative phosphorylation, photosynthesis, glutathione metabolism and sulfur metabolism. Finally, authors identify two non-SULTR genes potentially involved in Se transport, including ABCF5 and ABCG40, two ABC transporters with increased expression upon Se treatment. However, their mechanistic involvement in Se transport remains to be confirmed. | (Rao *et al.*, 2021b) |
| Transcriptome | *Brassica napus* | 15-day old seedlings in full Hoagland-Arnon nutrient hydroponics were treated with or without 50𝞵M CdCl_2_. The Cd-treated plants were also treated with or without 5, 10 or 20 mg Na_2_SeO_3_ /L or 5, 10 or 20 mg Nano-Se/L for 20 days (8 treatments total; 1 control, 1 Cd and 6 Cd+Se treatments). Se was applied via foliar spray three times. | Cd significantly decreased root and shoot biomass of *B. napus* compared to control, and increased levels of hydrogen peroxide, superoxide and ﻿malondialdehyde (a measure of lipid peroxidation), and all Se+Cd treatments reduced hydrogen peroxide, superoxide and MDA levels. All except one Se+Cd treatments improved plant growth compared to Cd treatment, except for the 20 mg/L selenite treatment (which contained higher tissue Cd levels), while 20 mg/L selenium nanoparticles had an even more positive biomass effect than 5 or 10 mg/L, almost completely restoring biomass to no Cd treatment levels. Compared to Cd treatment, the expression of respiratory burst oxidase homolog (RBOH) genes of Cd+Se treated plants was lower, including RBOHC, RBOHD and RBOHF genes. The transcriptomic response to Cd was characterized by changes in intracellular transport (membrane bounded organelle, vacuole), transcriptional regulation, transmembrane transporter activity (amino acid, cation, anion, organic acid), response to oxidative stress and sulfate assimilation. Compared to Cd treatment, the Na_2_SeO_3_ treatment affected transmembrane transport (amino acid, cation, anion, carboxylic acid peptide), sulfate assimilation, vacuoles and nuclear DNA (nucleosome, DNA packaging, chromatin, DNA binding, protein-DNA complex). Compared to Cd, the Nano-Se treatment affected mainly genes involved in the central DNA-RNA-Protein biogenesis pathway (cell cycle, ribosomes, double stranded DNA binding, maturation of rRNA, chromatin, mRNA splicing) as well as amide biosynthesis. | (Qi *et al.*, 2021) |
| Transcriptome and proteome | *Triticum aestivum* (wheat cultivar Jimai22) | 2-week-old seedlings were exposed to 10 𝞵M Na_2_SeO_4_ for 0, 3 or 12 hours | This comprehensive study identified 11656 proteins and 133911 genes. Notable Se-responsive gene families were APS, OAS-TL, NFS, SULTR, GST, GPX, GRX, SOD, CAT, HSP, UDP-glucose flavonoid glycosyltransferase (UFGTs), sucrose-6-phosphate-hydrolases, archaeal phosphoglucose isomerases (APGIs) and malate synthases. Genes that showed differential expression between 0 and 3 or 12 hr Se treatment were related to (among others) photosynthesis (photosystem I, photosystem II, oxygen evolving complex, thylakoid, chlorophyll), nutrient reservoir activity (glucosidase activity), sulfate transmembrane transporter activity, vitamin B6 (pyridoxine) binding, cell walls (cellulose synthase, extracellular, polysaccharide), secondary metabolism (isoprenoid-, terpenoid and phenylpropanoid metabolism), amino acid metabolism (Tyr, Arg, Pro, Phe), chitin catabolism and antioxidant defence (glutathione, peroxisome). Proteins that show differential abundance between 0 and 3 or 12 hour timepoints were related to (among others) **response to stress** (oxidative stress, peroxide oxidoreductase activity, glutathione, plant hormone signalling), **defence response** (response to fungus, chitin catabolism, plant-pathogen interaction), structural components (cutin-, suberin- and wax biosynthesis), **secondary metabolism** (flavonoid- terpenoid- stilbenoid-, gingerol-, alkaloid- and phenylpropanoid metabolism), **photosynthesis** (chlorophyll, ), amino acid metabolism (Ala, Asp, Glu, Phe, Arg, Pro) and **lipid metabolism** (linoleic acid, glycerolipid, alpha linolenic acid). The response of wheat appears to be a change in primary carbon and energy metabolism, digging into reserve fuel and producing more/ repairing cell walls. Since Se treatment increased MDA content and permeability of the cell membrane, this might be an adaptive response to strengthen the tissue structure in the face of Se stress. | (Feng & Ma, 2021) |
| **Genotype screening & Ionomics** | ***Arabidopsis thaliana* (1135 accessions)** | **Plants grown in Jiffy peat pellets soaked in a nutrient solution with additional sub-toxic As, Cd, Co, Li, Ni, Se, Sr and Rb for four weeks. (For seed ionome, 8w stratification and 2-month seed maturation added.** | **Ionomic variation across a multitude of *Arabidopsis thaliana* accessions is recorded. Leaf Se and S content shows strong correlation in the tested growth conditions, with sulfur-rich accessions also showing high Se content. Interestingly, seed Se content showed a much weaker correlation with S levels in seed and leaf, suggesting a sulfur-independent pathway of seed selenium loading. In addition, leaf Se and S negatively correlated (R^2^ between -0.34 and -0.42) with leaf Mg, Ca and Sr levels, with leaf Se negatively correlating with Fe too (R^2^ = -0.38), suggesting a mutually exclusive accumulation of high iron, magnesium and calcium accumulation with high selenium content.** | (Campos *et al.*, 2021) |
| Transcriptome | *Zea mays* | Seeds planted in clean sand& vermiculite, sprayed 3x per day with ½ Hoagland’s with or without 0.1, 1, 10, 20 and 30 mM Na_2_SeO_3_ for 7 days. | Se treatment of 1 mM increased dry weight and plant height, with Se content around 40 mg Se/kg dry shoot and ~8 mg Se/kg dry root. Se treatments from 10 mM onwards significantly reduced plant height and dry weight, and Se content increased gradually with increasing Se treatment. Interestingly, at treatments at or below 10 mM, the [Se]_shoot_ was higher than [Se]_root_ but this reversed at 20- and 30 mM treatments, with 30 mM resulting in ~80 mg Se/kg dry shoot and ~140 mg Se/kg dry root. The 1- and 10-mM treatments were assessed using transcriptomics, with the latter treatment affecting a larger number of genes (upregulated 845 and downregulating 1686 genes), and the former treatment affecting 345 genes, showing increased activity of genes involved in DNA replication, mismatch repair and homologous recombination compared to control. Both the 1- and 10-mM treatment activated various GSH-related genes, including GST, GSH2, GR. The higher Se treatment further repressed genes in auxin signalling and lignin biosynthesis, including SCF, SKP, SAUR, GH3, PAL, 4CL, CCR, CAD, LAC, and POD, some of which were activated in the lower Se treatment. | (Dou *et al.*, 2021) |
| Transcriptome & Metabolome | *Brassica oleracea* | 4x watering with 0 – 1.6 mM SeO_4_ | 0.1 mM SeO_4_ promoted growth, >0.8 mM reduced growth. Se, S, SeCys_2_ and SeMet increased significantly with Se application. Glucosinolate, phenolic acid and flavonoid metabolism affected by Se treatment. Se reduced MMT expression and increased expression of SULTR3;1 HMT, APS and APK. | (Yang *et al.*, 2022) |
| Transcriptome and proteome | *Camellia sinensis* | One year old tea cuttings placed in ¼ strength nutrient solution with or without 5 𝞵M Na_2_SeO_3_ or 5 𝞵M Na_2_SeO_4_ for 48 hours. Roots were sampled. | Leaf Se content was significantly increased by selenate (~0.26 mg Se/kg dry leaf) but not selenite treatment (~0.06 & ~0.08 mg Se/kg dry leaf for selenite and control, respectively), while root Se was most strongly increased by selenite (~19.5 mg Se/kg dry root) but still increased by selenate (~12 mg Se/kg dry root). Transcriptomic responses of tea to selenite- and selenate were quite dissimilar, with only 99 differentially expressed genes (DEGs) overlapping the two Se treatments and 806- 939 and unique DEGs for selenite and selenate respectively, and similar numbers for the differentially expressed proteins (DEPs). The selenite treatment affected genes and proteins involved in (among others) glutathione metabolism, proteasome, protein processing (endoplasmic reticulum, ribosome), terpenoid- and phenylpropanoid metabolism, central carbon metabolism (TCA cycle, gluconeogenesis), nitrogen and amino acid metabolism (Cys, Met, Ala, Asp, Glu, Lys, Tyr, Val, Leu, Iso), thiamine metabolism, taurine metabolism and lipid metabolism (linoleic acid, sphingolipid, alpha linolenic, unsaturated fatty acids). Selenate treatment affected genes involved in (among others) **RNA metabolism** (RNA transport, spliceosome, RNA polymerase, RNA degradation, purine metabolism), **vitamin B metabolism** (thiamine B1, pyridoxine B6, folate B9), **nitrogen and amino acid metabolism** (Val, Leu, Iso, Arg, Pro, Ala, Asp, Glu, Tyr, Lys), **ABC transporters**, **flavonoid- and ubiquinone biosynthesis**, **gluconeogenesis, DNA maintenance** (mismatch repair, non-homologous end-joining) and **lipid metabolism** (arachidonic acid metabolism, glycerolipid metabolism, fatty acid degradation) | (Ren *et al.*, 2022) |
| Transcriptome | *Medicago sativa* cultivar Kangsai (high Se accumulation variety) | Planted in experimental station field in China, with fertilization of 750 kg Na_2_SeO_3_ / ha and then spraying with 100 mg Na_2_SeO_3_ /kg (sub-toxic), leaf samples taken after 0, 12 and 48 hours after spraying. | `  After 12h, the Se level was highest in leaves (~11.8 mg Se/kg DW) and lowest in stems (~0.5 mg/kg DW), which balanced out at 24- and 48h timepoints (~9.5 mg Se/kg dry leaf; ~4.3 mg Se/kg dry stem). Genes with initial increased expression and later attenuation (e.g. early Se activated or late Se repressed) were related to (among others) central carbon metabolism (TCA cycle, gluconeogenesis, pyruvate, starch& sucrose, pentose phosphate pathway), glutathione metabolism, indole alkaloid biosynthesis and lipid metabolism. Genes that were downregulated initially but restored or enhanced later (e.g. early Se repressed & late Se activated) were related to (among others) terpenoid- and polyketide biosynthesis, peroxisomes, photosynthesis (chlorophyll & antenna proteins), metabolism of cofactors (), amino acid metabolism, carotenoid metabolism. Processes both showing an early Se activated and early Se repressed response were transport and catabolism, membrane transport, signal transduction, cellular maintenance (folding, sorting, degradation, replication and repair, transcription& translation) and cysteine and methionine metabolism. Metabolomic analysis also indicated an initial (12h) decrease in chlorophyll and carotenoids and increase in H2O2, GSH and MDA, all of which were attenuated after 48h. | (Hu *et al.*, 2022) |
| Transcriptome and metabolome | *Cardamine violifolia* | 2-year-old plants grown at commercial farm in Enshi, China. Inner- and outer leaves were sampled. | Metabolome analysis indicated a significant difference in transcript profile between inner- and outer leaves, with the former containing mostly higher levels of lipids, amino acids, alkaloids and nucleotides, while containing mostly lower levels of phenolic acids, flavonoids and other secondary metabolites. This intuitively suggests inner leaves, being younger, will have a larger ratio of metabolism dedicated to central metabolism required for cell growth, while older outer leaves will dedicate more energy towards defence metabolism. Comparing the transcriptome of the leaf types also indicates major differences in (among others) alkaloid biosynthesis, phenylpropanoid biosynthesis, nitrogen and amino acid metabolism (Cys, Met, Ala, Asp, Glu), central carbon metabolism (glyoxylate- dicarboxylate metabolism, fructose- and mannose metabolism, starch and sucrose metabolism and glycolysis& gluconeogenesis), plant pathogen interaction, plant hormone signalling, peroxisomes and lipid metabolism (fatty acid degradation, glycerolipid metabolism). | (Rao *et al.*, 2022) |
| Transcriptome | *Medicago sativa* cultivar Kangsai (high Se accumulating variety) | Planted at experimental field station, with or without spraying 100, 200, 300 and 500 mg Na_2_SeO_3_ / kg three times per day for seven days before harvesting top leaves. | 100 mg/kg selenite treatment (resulting in ~18 mg Se/kg dry leaf) positively affected alfalfa growth, increasing hay yield and phosphorous content and decreasing H2O2 and MDA and increasing pigment contents and photosynthetic properties compared to control. 500 mg/kg selenite was toxic to alfalfa (resulting in ~39 mg Se/kg dry leaf), inhibiting photosynthesis by 20%, decreasing phosphorous content and promoting lignin biosynthesis. This study thereby represents a typical hormesis curve. Transcriptional comparison of 100 mg/kg selenite versus control treated plants revealed a significant Se-response of genes involved in (among others) photosynthesis and central carbon metabolism (chlorophyll metabolism, pentose phosphate pathway, carbon fixation, fructose- and mannose metabolism), secondary metabolism (polyketide sugar metabolism, anthocyanin biosynthesis), central nitrogen metabolism (amino- and nucleotide sugar metabolism, Val-, Leu-, Iso-, Phe-, Arg-, Pro-, Tyr- and Trp metabolism), lipid metabolism (glycerolipid-, linoleic acid-, butanoate- and arachidonic acid metabolism), plant pathogen interaction and protein biogenesis (ribosome, protein processing in endoplasmic reticulum). Similar methods as (Hu *et al.*, 2022). | (Wang *et al.*, 2022) |
| Metabolome and transcriptome | *Triticum aestivum* | A field experiment where plants 10d after flowering were treated with or without a 0.02 g/L Na_2_SeO_3_ solution, to a total of 40 g Se/ha. 10 days after spraying, immature grains were harvested for analysis. | Wheat flower of Se treatment contained significantly more Se (~0.280 mg Se/kg flour) compared to control (~ 0.120 mg Se/kg flour), mostly in form of SeMet and SeCys_2_, and Se-treated grains had darker colours, more anthocyanins and more flavonoids. Transcriptome analysis indicated that Se treatment significantly affected the expression of transcription factors as well as various enzymes involved in flavonoid biosynthesis. | (Zhang *et al.*, 2022) |
| Transcriptome | ﻿*Vigna radiata* (Mung beans) | Mung beans were immersed in 0, 20 and 80 mg/kg Na_2_SeO_3_ solutions, then sprouted for 5 days and root- and leaf samples for transcriptome were taken. | Low concentrations of Se (<50 mg/kg) enhanced the sprouting of mung bean, increasing the fresh- and dry weight, but concentrations higher than 50 mg/kg (resulting in >27 mg Se/kg dry mung bean sprout) reduced sprouting rate and shortened the hypocotyl significantly. The transcriptomic analysis indicated that Se-treated roots showed a reduced expression of NFS, PHT1.1, PHT1.4 and PHT1.X2, (phosphate transporters and Fe-S cluster biosynthesis) while leaves of high Se treated beans showed increased expression of SMT, NIP2, SULTR3;3, APS1, NFS and PHT1 (selenite and selenate transport and Se(MeCys) assimilation), while both Se-treated leaves and roots showed increased expression of HMT, MS and a GST (methionine metabolism and glutathione metabolism). | (Cheng *et al.*, 2023) |
| Transcriptome and metabolome | *Medicago sativa* (alfalfa) | Seedlings were grown in vermiculite and perlite irrigated with ½ Hoagland’s, then sprayed with 100 mg/L Na_2_SeO_3_ solution (sub-toxic) or distilled water, after which whole leaves were harvested | Se treatment increased plant dry weight, total Se content (0.684 mg Se/kg dry leaf), pollen generation and concentrations of chlorophyll, soluble protein, proline and glutathione, while decreasing MDA content. Transcriptome analysis indicates significant responses of genes involved in (among others) MAPK signalling, secondary metabolism (flavonoid-, phenylpropanoid-, stilbenoid, diarylheptanoid- and gingerol biosynthesis,), cutin- suberin- and wax biosynthesis, lipid metabolism (alpha linolenic- and glycerolipid metabolism), cysteine and methionine metabolism and glycolysis/ gluconeogenesis. Metabolome analysis confirmed effects on flavonoid and phenylpropanoid metabolism, and further showed changes in nitrogen- and amino acid metabolism (Arg, Ala, Asp, Glu, His, Phe), glutathione metabolism and central carbon metabolism (butanoate, propanoate, glyoxylate and dicarboxylate metabolism) | (Wang *et al.*, 2023d) |
| Transcriptome | *Oryza sativa* | Surface sterile seed placed in sterilized mixture of soil-quartz: sand (1:3). Treatment with or without Se and with either sterilized or alive arbuscular mycorrhizal fungi material (species: *Funneliformis mosseae).* Roots used for transcriptome analysis. | Compared to control, all AMF treatments increased plant biomass production. Compared to control treatment, Se treatment increased levels of Se and Ca but decreased levels of Co, Cu and Al in root and shoot. In the shoot, Se increased levels of Mg and decreased levels of Ni, but in the root, Se decreased levels of Mo. Compared to control treatment, Se treatment significantly affected genes related to (among other) protein biogenesis and degradation (proteasome, ribosome, RNA transport, aminoacyl-tRNA biosynthesis), signalling pathways (MAPK signalling, phosphatidylinositol signalling), plant-pathogen interaction, glutathione metabolism, lipid metabolism (alpha-linolenic-, linoleic- and glycerolipid metabolism), flavonoid metabolism, TCA cycle, peroxisome and endocytosis. | (Qin *et al.*, 2023) |
| Transcriptome and metabolome | *Cardamine violifolia* | Two-year old seedlings were harvested from a commercial farm in Enshi, China, and divided into central leaves, outer leaves, petioles and roots. Central leaves were younger leaves. | Glucosinolate content was highest in roots, then central leaves and finally lowest in outer leaves and petioles. Transcriptomic comparison between older and younger leaves indicates significant differences in the expression of genes involved in (among others) biosynthesis of secondary metabolites (alkaloids, phenylpropanoids), MAPK signalling pathways, plant hormone signalling, gluconeogenesis, fructose- and mannose metabolism, nitrogen metabolism and peroxisomes. | (Rao *et al.*, 2023) |
| Transcriptome | *Brassica rapa* subspecies *chinensis*  (pak choi) | Seeds germinated for 12d in vermiculite, then transferred to 2.5 L hydroponics nutrient solution for 3 weeks, then treated with or without 10 𝞵M Na_2_SeO_3_ or Na_2_SeO_4_ and with or without 10 𝞵M Cd(NO_3_)_2_ (6 treatments) for 24, 72 and 168 hours (1, 3 or 7 days), after which roots were used for transcriptome analysis. | Selenite but not selenate effectively lowered Cd concentrations in the shoot, while both Se treatments lowered Cd levels in the roots after 7d of treatment. The shoot Se levels of selenite treated plants was very low (~1 mg Se/kg dry shoot) compared to selenate treated plants (~21.5 mg Se/kg dry shoot), while roots Se levels were more similar between selenite (~15.5 mg/kg dry root) and selenate treated plants (~24 mg Se/kg dry root). Cd decreased shoot Se levels in both selenate and selenite treatments, and root Se level of selenate treated plants. Selenite was more readily transformed into SeMet and SeMeCys, with only ~25% of Se as SeO_4_^2-^ in shoots and 8-11% of Se as SeO32- or SeO42- in roots, while selenate-treated plants contained 67-68% of Se as SeO_4_^2-^ in the shoots and 30-38% of Se as SeO_3_^2-^ or SeO_4_^2-^ in the roots. Cd treatment did not change the Se speciation in a major way. Selenite treatment upregulated genes involved in lignin biosynthesis, suberin biosynthesis and phytochelatins, as well as stress signalling genes. Selenate treatment decreased the expression of some Cd-responsive genes, such as PCS2, ABCC6, GPAT6, which were even more activated with selenite + Cd. This inverse transcriptomic response in the phytochelatin detoxification pathway (PCS2 and ABCC6) by selenate might explain part of the lower effectiveness in root Cd retention. | (Yu *et al.*, 2023) |
| Transcriptome | *Medicago sativa* (alfalfa, cultivar Kangsai, a Se rich variety) | 0.75 kg/hm^2^ Na_2_SeO_3_ was sprayed on 20 cm tall alfalfa plants three times for three weeks, then shoots were harvested. | Se treatment significantly increased height and biomass of plants, as well as the Se content (~18 mg Se/kg dry shoot), soluble sugar- and GSH content, photosynthetic efficiency and chlorophyll levels. Se treatment also reduced the levels of H_2_O_2_, MDA, and superoxide. Transcriptome comparison between control and Se treatment indicates responses of transcription factors, DNA replication and repair mechanisms, photosynthesis and carbohydrate metabolism as well as protein biogenesis and processing. | (Wang *et al.*, 2023a) |
| Transcriptome | *Triticum aestivum* (four cultivars with white, blue, purple and black grain colors) | Wheat plants were grown in a silty clay loam field ( 0.4 mg Se/kg soil) in Shanxi province, China. Plants were sprayed with a 0 or 50 mg Se/L solution for a total of 0 or 37.5 g Na_2_SeO_3_ /ha. Transcriptome analysis was performed on grains that were ripened for one month. | Se treatment increased the concentrations of chlorophyll *a* and *b* and carotenoids as well as Se level. Interestingly, the blue, purple and black wheat varieties showed a higher uptake of Se than the white wheat variety, even at the control treatment, while the Se treatment reduced these relative differences. The transcriptomic differences between the varieties were much larger than the control-treatment differences as seen from the principal component analysis (PCA).  Genes related to anthocyanin-, phenylpropanoid- and flavonoid biosynthesis were significantly upregulated by Se treatment. Other processes significantly affected by Se treatment were (among others) **glutathione metabolism** (white& blue variety), **phenylpropanoid metabolism** (white, blue and black var.), **flavonoid biosynthesis** (blue, purple & black), **anthocyanin metabolism** (purple & black), **thiamine metabolism** (blue), **nitrogen& amino acid metabolism** (blue, purple & black; Cyanoamino acid, Phe, Arg, Pro, Ala, Asp, Glu, Gly, Ser, Thr, Trp, Tyr, Val, Leu, Iso, Cys, Met), **lipid metabolism** (white, purple & black; **glycerophospholipid metabolism,** alpha linolenic acid**)**, **plant hormone signalling** (white), and **protein biogenesis and degradation** (white, blue & purple; phagosome, ribosome), **ubiquinone and terpenoid biosynthesis (**blue), **plant-pathogen interaction** (blue), **lipid metabolism** (blue; unsaturated fatty acids, ), **nicotinate and nicotinamide metabolism (**blue), **starch and sucrose** **metabolism** (blue, purple, black), **photosynthesis** (purple & black), **MAPK signalling** (purple & black), **amino sugar and nucleotide sugar metabolism** (blue, purple & black), **taurine and hypotaurine metabolism** (purple & black), **alkaloid metabolism** (black) and **cutin-, suberin- and wax biosynthesis** (purple, black) | (Xia *et al.*, 2023) |
| Transcriptome | *Glycine max*  (Soybean variety Dongnong 690) | 3.8L hydroponics treated with 0, 20 or 100 𝞵M of selenium nanoparticles (100-600 nm size) for 7 days. Root and leaves were used for transcriptome analysis | Treatment with nano selenium resulted in an increased Se content of root (218- and 518 mg Se/kg dry root for 20- and 100 𝞵M treatments resp.) and leaf (15.5- and 42.9 mg Se/kg dry leaf for 20- and 100 𝞵M treatments resp.) compared to control (3.20 mg Se/kg dry root, 1.45 mg Se/kg dry leaf). SeMet was the dominant selenocompound in the leaves (55-84%) and roots (20.5 – 78.7%), with SeCys_2_ and MeSeCys becoming more prominent at higher Se treatments (6-17% in leaves, 19-25% in roots). Inorganic species of Se were mainly in SeO_4_^2-^ form, varying between 20-24% in root and 11-14% in leaf, while SeO_3_^2-^ abundance in Se-treated plants was low (2-7% in root and leaf). | (Xiong *et al.*, 2023) |
| Transcriptome, ionome, metabolome | *Oryza sativa* | 1 week old rice seedlings were grown in hydroponics with 0 or 0.5 mg/L Na_2_SeO_3_ and 0 or 1 mg/L CdCl_2_ for one week. Roots were sampled for transcriptome analysis. | Cd treatment resulted in ~65 mg Cd/kg dry shoot, which was attenuated by Cd+Se treatment to ~14 mg Cd/kg dry shoot, while 900 mg Cd/kg dry root was attenuated to ~225 mg Cd/kg dry root by combined Cd+Se treatment. Se levels reached high concentrations in Se-treatment (~95 mg Se/kg dry shoot; ~350 mg Se/ kg dry root) and significantly lower levels in Se+Cd treatments (~27 mg Se/ kg dry shoot; ~200 mg Se/kg dry root). However, no growth rate- or biomass data is presented, so it is not clear what the effect on plant biomass production was. Combined Se+Cd significantly increased content of lignin, hemicellulose, callose and pectins. Cluster analysis of the transcriptomic data shows that Cd and control treatments have large within-group variation, while the Se and Se+Cd treatments show a more consistent root transcriptome. Major gene-ontology terms over-enriched in the Cd vs. Cd+Se comparison were related to 1) Iron homeostasis, 2) water homeostasis and transport, 3) scopolin beta-glucosidase activity, 4) peroxidase activity and 5) cofactor catabolic process. Major KEGG pathways over-enriched in the Cd vs. Cd+Se comparison were related to 1) phenylpropanoid metabolism, 2) starch and sucrose metabolism, 3) linolenic acid metabolism, 4) taurine and hypotaurine metabolism, 5) nitrogen- and amino acid metabolism (cyanoamino acid, Tyr, Cys, Met), 6) plant hormone signal transduction, 7) flavonoid- and alkaloid biosynthesis and arachidonic acid metabolism. | (Wang *et al.*, 2023c) |
| Metabolome | *Zea mays* | 15-day old seedlings were treated with ultrapure water (control treatment) or ultrapure water with 2 or 20 mg/L selenonanoparticles or 2 or 20 mg/L Na_2_SeO_3_ for 5 days after which leaves were harvested. | Se treatments increased catalase activity, and high Se treatments (20 mg/L) also increased GSH content and (ascorbate) peroxidase activity. Treatment with 20 mg/L nano-Se also significantly altered metabolic profiles, especially relating to 1) nitrogen and amino acid metabolism (Ala, Asp, Glu, Arg, Tyr, Cys, Met, Gly, Ser, Thr, Pro, Trp, Phe), 2) secondary metabolites (alkaloids, and betalain), 3) central carbon metabolism (TCA cycle, glyoxylate and dicarboxylate, C5-brnached dibasic acid), 4) glutathione metabolism and 5) amino sugar- nucleotide sugar-, purine and pyrimidine metabolism. | (Wang *et al.*, 2023b) |
| Transcriptome and metabolome | *Oryza sativa* | 5L hydroponics with IRRI nutrient solution. 1w old seedlings were treated with or without 0.5 mg/L Na_2_SeO_3_ and with or without 1 mg/L CdCl_2_ for 1 week before harvest (CK, Se, Cd and Cd+Se treatments). Roots samples for transcriptome and metabolome analysis | Selenite treatment significantly reduced root and shoot Cd uptake in the Se+Cd treatment (~12 mg Cd/kg dry shoot; ~210 mg Cd/kg dry root) compared to Cd treatment (~50 mg Cd/kg dry shoot; ~750 mg Cd/kg dry root). Se treatment reduced root surface & tip numbers, enhanced the concentration of tartaric acid in the root exudates as well as root levels of auxin (IAA) and jasmonic acid (JA) and their derivatives and precursors. Transcriptome analysis indicated upregulation of genes involved in biosynthesis of JA and gibberellin (GA53), but not IAA. Root growth inhibition is hypothesized to correlate with signal transduction of IAA and GA53, altering polar IAA transport and homeostasis. | (Wu *et al.*, 2024) |
| Genotypic screening & transcriptome | *Triticum aestivum* (var. Jimai 22; this study generated Se-rich mutant of this variety) | Two-week-old seedlings were treated with 0 or 10 𝞵M Na_2_SeO_3_ for 9 hours, transcriptome of whole seedling was performed. Methods similar to (Feng & Ma, 2021) | Seed Se content was significantly increased in mutant wheat line **J52-2** (~0.205 mg Se/kg dry seed) compared to the **wildtype** **J22** (~0.13 mg Se/kg dry seed). The Se-rich J52-2 mutant also had an increased POD and SOD activity in salt treatment compared to wildtype, which was supported by higher gene expression of some SOD and POD genes, and better retained germination rates- and root lengths at 300𝞵M Na_2_SeO_3_ and 200 mM NaCl treatment. Further analysis of the transcriptome reveal that J52-2 sports a strongly increased expression of (among others) various GST genes, lipoxygenases, zinc transporter and metallothionein, linoleate 9S lipoxygenase, peroxygenases, CoA-biosynthesis genes, multiple dirigent proteins, heat shock proteins and chaperones, alternative oxidase and MMT, while J52-2 showed decreased expression of chlorophyll biosynthesis genes, a nicotianamide synthase, multiple peroxidases, some chaperones/heat shock proteins, APS2, ACO, SAMS, ABC transporter (Al^3+^ tolerance associated) | (Shi *et al.*, 2024) |
| Transcriptome | *Broussonetia papyrifera* (paper mulberry) | Leaves were taken, placed on agar for regenerative shoot formation. These plantlets were placed back in the greenhouse and were submitted to spraying once a week with 0, 200, 400 or 800 𝞵M Na_2_SeO_3_ or Na_2_SeO_4_ for four weeks. Leaves were taken for RNA analysis. | The authors focus on the expression of NAC genes in *B. papyrifera* and find that selenate treatment correlates with the expression of *BpNAC59* and *BpNAC62*, while selenite treatment correlates well with *BpNAC55*. The role of *BpNAC* transcription factors in *B. papyrifera* Se responses is therefore quite interesting and warrants further investigation. | (Guo *et al.*, 2024) |
| Transcriptome and metabolome | *Malus domesticus* (Apple, variety ﻿G935) | Tissue culture seedlings were planted on foam boards and supplied with 1w ½ Hoagland’s, then 1x Hoagland’s. Healthy plants were then exposed to 0, 3, 6, 9, 12, 24 and 48 𝞵M Na2SeO3 for 28 days. Roots were used for metabolome- and transcriptome analysis. | Low Se treatment increased apple growth, with 9 𝞵M selenite treatment increased the total dry weight by 84% compared to control treatment. However, high Se treatments (>24 𝞵M) reduced apple growth, disturbing photosynthesis, carbon- and nitrogen metabolism and causing oxidative damage, with 48 𝞵M-treated plants also showing reduced root growth and reducing total dry weight by more than half. Soluble protein, catalase, peroxidase and superoxide dismutase activity and photosynthetic performance peaked at 9 𝞵M yet reduced at >12 𝞵M Se treatments. Interestingly, the hormesis curve is inversed in free amino acid, NH_4_^+^ and NO_3_^-^ content, which showed a minimum at the 9 𝞵M treatment and maximum at 48 𝞵M, while glycose, fructose, sucrose and sorbitol also increased along the stronger Se treatments, as did MDA content. These results present another example of Se hormesis and suggest that high sugar- and free amino acid levels are a symptom of inhibited growth, perhaps due to the halted cell growth and development, causing a buildup of raw material. From the transcriptomic- and metabolomic analysis it is clear that control and 9 𝞵M Se treatments show a more similar gene expression fingerprint, while the 24 𝞵M Se treatment shows a broadly different pattern. Genes affected by Se treatment were (among others) related to 1) **nitrogen and amino acid metabolism** (Trp, Tyr, cyanoamino acid, Cys, Met,), 2) **sugar metabolism** (starch- and sucrose metabolism, fructose, galactose and mannose metabolism**)**, 3) **phenylpropanoid metabolism**, 4) **central carbon metabolism** (glycolysis/ gluconeogenesis, pentose and glucuronate, pentose phosphate pathway), 5) **secondary metabolism** (stilbenoid- terpenoid- diarylheptanoid- and gingerol biosynthesis, carotenoid biosynthesis, alkaloid biosynthesis), 6) **taurine and hypotaurine metabolism,** 7) **cutin-, suberin- and wax biosynthesis**, 8) **MAPK signalling**, 9) **plant hormone signalling** (zeatin, a cytokinin; brassinosteroid), 10) **DNA replication**, 11) **ABC transporters**, 12) **glutathione metabolism**, 13) **lipid metabolism** (alpha linolenic acid metabolism, glycosphingolipids biosynthesis) and 14) **glycan degradation** (glycosaminoglycan degradation). Metabolome data further indicates alterations in 15) **cofactor metabolism** (thiamine, CoA, pyridoxine), 16) **flavonoid biosynthesis** and 17) vitamin C (ascorbate) metabolism. | (Liu *et al.*, 2024) |
| Transcriptome | *Cyphomandra betacea* | Seedlings were grown in moist perlite watered with 1x Hoagland solution, with or without addition of 0.1 mg/L Na_2_SeO_3_ and/or 1 𝞵M abscisic acid (ABA); four treatments total (CK, ABA, Se, Se+ABA). Leaves were used for transcriptomic analysis. | Compared to control, treatments with Se and/or ABA generally had a negative impact on plant physiology, reducing above- and belowground biomass significantly, along with chlorophyll- and carotenoid content. However, activity of superoxide dismutase (SOD), peroxidase (POD) and catalase (CAT) were generally increased by the treatments, and Se treatments drastically increased plant Se content (~5.5 mg Se/kg dry shoot & ~5.5 mg Se/kg dry root). ABA treatments strongly increased the expression of SULTR1;2, CGS, MS1, APR1, APS1, SAT3 and PHT1;4 compared to control and Se treatment alone, suggesting that ABA promotes S transport and assimilation pathways and thereby aid in Se accumulation and assimilation as well. [red. There is a link between ABA levels and chloroplastic sulfur, linked to the ABA3 gene required for aldehyde oxidase (AO) activity] | (Wang *et al.*, 2024a) |
| Transcriptome | *Oryza sativa*  (var. ﻿Xiuzhan 15) | 2w old seedlings transplanted to 300g soil with or without 30 mg/kg CdCl_2_ and/or 30 mg/kg nano-Se (two types) or Na_2_O_3_ for one month. Roots were used for transcriptome analysis. | Cd treatment more than halved the shoot biomass of the rice plants and drastically reduced photosynthetic capacity, yet the 30 mg/kg selenite treatment alone or with Cd performed even worse, while nano-Se treated plants either showed identical shoot biomass or minor decreases when applied together with Cd. The Se levels of the selenite treatment were much higher (~225 mg Se/kg dry root, ~130 mg Se/kg dry shoot) compared to the nano-Se treatments (10 - 20 mg Se/kg dry root, 1.5 - 2.5 mg Se/kg dry shoot). This brings home the message that nano-Se has a much lower bioavailability, and therefore toxicity, compared to ionic forms such as selenite. Investigating the transcriptional differences in Cd and Cd+nano-Se treated plants reveals a significant response of genes involved in (among others) 1) **phenylpropanoid biosynthesis**, 2) **sugar metabolism** (starch-, sucrose- and galactose metabolism), 3) **central carbon metabolism** (glycolysis/ gluconeogenesis, pyruvate metabolism), 4) am**ino sugar- and nucleotide sugar metabolism**, 5) **nitrogen and amino acid metabolism** (Cys, Met), 6) **secondary metabolism** (diterpenoid- flavonoid- cutin-, suberin- and wax biosynthesis), 7) **plant hormone signalling**, 8) **MAPK signalling**, 9) **peroxisomes**. In-depth pathway analysis indicates that Se alleviated the Cd-induced upregulation of glycolysis (glucose 🡪 TCA cycle) and changes to antioxidant- cysteine- and nitrogen metabolism, as well as upregulating the Cd-supressed auxin-dependent signalling pathway. | (Shang *et al.*, 2024) |
| Metabolome | *Triticum aestivum* | 15-day old wheat seedling grown in Cd-contaminated soils were sprayed with 0, 10, 20 or 40 mg/L nano-Se or 0, 50, 100 or 200 mg/L nano-SiO_2_/ nano-ZnO or MnO_2_. Spraying was done 12 times in total, and 5 days after the spraying samples for metabolomics were taken. | Selenium and silicon nanoparticles were most effective at reducing the Cd uptake of wheat, decreasing Cd content by 33-35%. The grain yield was also increased by nano-Se treatment (+33%). Si- and Se-nanoparticles downregulated Cd transport proteins NRAMP5 and LCT1, while upregulating the vacuolar sequestration genes HMA3 and TM20. | (Wang *et al.*, 2024b) |
| Transcriptome | *Oryza sativa & Brassica chinensis* (rice and pak choi) | Seedlings were placed in 600g soil, then after 7d treated with or without 50 ml of 100 mM NaCl solution (salt treatment), then after 7d the salt-treated plants were treated at the roots with 50 ml of 0, 100, 300, or 500 mg/L chitosan nano-Se particles for 7 days. Rice leaves were used for transcriptome analysis. | Se-chitosan nanoparticles alleviated the salt-induced reduction in dry weight, while the highest (500mg/L) treatment did not do as well as the lower treatments (100- and 300 mg/L). The nanoparticles increased antioxidant enzyme activity, such as superoxide dismutase, catalase and peroxidase. Malondialdehyde (MDA) levels were increased by NaCl treatment but brought back to control levels by nano-Se treatment. Interestingly, the Na and K levels in the root and shoot were generally not altered by the nanoparticles, suggesting that the nanoparticles mainly addressed the toxicity effect of Na, rather than prevent its uptake. Transcriptomic analysis indicates that the gene expression fingerprint of control and 300 mg/L nano-Se + NaCl treatment were quite similar, while the NaCl treatment was much different. Compared to salt treatment alone, the nano-Se + NaCl treatment increased expression of genes involved in (among others) 1) **sulfur metabolism**, 2) **nitrogen- and amino acid metabolism** (Tyr, pyridoxine metabolism, cyanoamino acid metabolism), 3) **secondary metabolism** (terpenoid-, carotenoid-, flavonoid- and phenylpropanoid biosynthesis), 4) **lipid metabolism** (linoleic acid metabolism, alpha linolenic acid metabolism), 5) **plant-pathogen interaction**, 6) **base excision repair**, 7) **galactose metabolism** and 8) **MAPK signalling**. | (Fang *et al.*, 2024) |
| Transcriptome | *Medicago sativa* | Plants were field grown at an experimental station in XinXiang, China (0.48 𝞵g Se/kg background). 20cm tall plants were sprayed with 0 or 500 mg/kg Na2SeO3 three times for three weeks. Mature leaves were used for transcriptome analysis. | Se treatment increased Se levels significantly (~38 mg Se/kg dry leaf), while plant height and biomass were slightly but significantly reduced. Photosynthesis was significantly inhibited by Se treatment, and antioxidant metabolism was unbalanced: the peroxidase activity was reduced and the H_2_O_2_ content was increased. Fiber- and lignin content of alfalfa was significantly increased by Se treatment, while free protein levels were reduced. Compared to control conditions, the Se condition significantly upregulated genes involved in (among others): 1) **aromatic compound biosynthesis** (phenylpropanoid-, flavonoid-, stilbenoid-, diarylheptanoid-, gingerol-, Tyr, Trp and Phe metabolism), 2) **lipid metabolism** (alpha-linolenic acid metabolism, sphingolipid metabolism, ether lipid metabolism, fatty acid degradation, linoleic metabolism), 3) **glutathione metabolism**, 4) **secondary metabolism** (limonene- and pinene degradation), 5) **cyano amino acid metabolism**, 6) **pyridoxine metabolism** and 7) **sugar metabolism** (gluconeogenesis, galactose metabolism). Processes downregulated by Se treatment were (among others) 1) **aromatic compound metabolism** (flavone- and flavanol metabolism), 2) **lipid metabolism** (linoleic acid-, alpha-linolenic acid-, glycerolipid and arachidonic acid metabolism), 4) **secondary metabolism** (terpenoid-, diterpenoid-, triterpenoid and sesquiterpenoid metabolism), 7) **sugar metabolism** (starch and sucrose metabolism, pentose- and gluconerate interconversions), 8) **peroxisomes,** 9) protein degradation (protein processing in ER, phagosome, autophagy), 10) amino acid metabolism (valine, leucine, isoleucine, beta-alanine and lysine metabolism), 11) plant hormone metabolism and 12) MAPK signalling. | (Hu *et al.*, 2024) |
| Metabolomics and proteomics | *Triticum aestivum* | Plants were grown in 1.5kg natural soil from an experimental station in Beijing, China. Pesticide (﻿bensulfuron methyl) was added to the soil, and the plants were sprayed with either 0 or 5 mg/L nano-Se and/or 10 mg/L melatonin (four treatments: CK, Se, MT, Se+MT) | Combined nano-Se and melatonin treatment was more effective in reducing pesticide damage than either treatment alone. Metabolomic and proteomic analysis indicated that combining the two treatments activated phenylpropanoid-, phenol- and flavonoid biosynthesis pathways in both leaves and root. In addition, the treatments adjusted the rhizosphere microbial communities, increasing soil urease and fluorescein diacetate enzymatic activity. | (Zhou *et al.*, 2024) |
| Transcriptome and metabolomics | *Solanum lycopersicum*  (tomato, cultivar Micro-Tom) | Plants were cultivated in ¼ Hoagland’s hydroponics treatment, and 21-day old seedling were exposed to 0 or 2 𝞵M Na_2_SeO_3_ for 5-7 days. Roots and leaves were used for transcriptomic analysis. | Se treatment increased plant height, root dry weight and shoot dry weight significantly, as well as increasing the soluble sugar- and chlorophyll content of the leaves and photosynthetic parameters. Genes affected by Se in the leaves are related to (among others) 1) signalling (auxin, ABA, cytokinin, ethylene, protein kinases, regulation of histone methylation), 2) regulation of metabolism (regulation of nitrogen compounds metabolism, regulation of glucan biosynthetic process, response to fatty acid), while genes affected by Se in the roots are related to (among others) 3) aromatic compound biosynthesis (flavanol biosynthesis), 4) ion transporter activity (nitrate transmembrane transporter activity), 5) Rubisco activity, 6) antioxidant metabolism and 7) heme- and chlorophyll biosynthesis. Metabolomics results confirm an increased concentration of auxin, jasmonic acid and salicylic acid in the leaves, and increased cytokinin in the roots, potentially playing a role in the response of tomato to Se. | (Li *et al.*, 2024) |
| Transcriptome | *Nicotiana tabacum* (tobacco, var. Yunyun 87) | Tobacco seedlings were grown in 4.5L ½ Hoagland’s hydroponics solution. 28d old seedling were treated with or without 50 𝞵M K_2_Cr_2_O_7_. Cr-treated plants were treated with or without 2 𝞵M Na_2_SeO_3_ and/or 1 𝞵M Na_2_MoO_4_ for 2 weeks, after which root and shoot samples were taken. | Cr treatment significantly reduced tobacco root- and shoot length and dry weight, which was partly restored by separate Cr+Se or Cr+Mo treatment, and completely restored by Cr+Se+Mo treatment (except for root dry weight). The biomass values negatively correlated with shoot Cr content, but positively correlated with root Cr content: Cr treatment resulted in the lowest root Cr content (~650 mg Cr/kg DW) and highest shoot Cr content (~65 mg Cr/kg DW), while Cr+Se+Mo treatment therefore had the highest root Cr content (~1575 mg Cr/kg DW) and lowest shoot Cr content (~25 mg Cr/kg DW). Root-based Cr detoxification and sequestration was therefore key in retaining normal plant physiology. While Cr treatment drastically lowered levels of pectin, cellulose and hemicellulose in root and shoot, Cr+Se+Mo partially restored these almost to control levels. On a transcriptomic level, the Cr+Se+Mo transcriptional fingerprint much more strongly resembled the control transcriptome both in leaves and roots, as compared to the Cr/ Cr+Se/ Cr+Mo treatments, which grouped together more closely. Compared to control, the Cd treatment significantly affected genes involved in (among others) 1) sugar metabolism (starch-, sucrose-, fructose-, and mannose metabolism, glycolysis & gluconeogenesis), 2) aromatic compound biosynthesis (phenylpropanoid- and phenylalanine metabolism), 3) sulfur metabolism (cysteine-, methionine-, glucosinolate and glutathione metabolism), 4) peroxisomes and 5) ABC transporters. Almost exactly the same processes are significantly differentially regulated in the Cr vs. Cr+Se+Mo treatment, which is not surprising remembering the similarity between Cr+Se+Mo and control condition. Between Cr and Cr+Se, there are also some overlapping processes with adjusted expression, in addition to 6) alpha-linolenic metabolism, 7) flavonoid biosynthesis and 8) plant hormone signalling. | (Qu *et al.*, 2024) |
| Transcriptome | *Triticum aestivum* (wheat cultivar Ningchun 4) | Plants were grown in 1x Hoagland’s hydroponics. One-month old plants were exposed to 0 or 10 𝞵M CdCl_2_ and 0 or 10 𝞵M nano-Se for 3 days. Roots were used for transcriptomics | Nano-Se application significantly reduced Cd accumulation in root (-56%) and shoot (-37.3%), while increasing cell wall Cd content by 54% and increasing lignin, pectin and hemicellulose contents. The transcriptomic data confirms upregulation of genes involved in cell wall biosynthesis. Combined treatment with Cd and Se also reduced root and shoot Se levels, suggesting a synergistic effect of both metal(loids) on uptake, transport and sequestration mechanisms, perchance a direct chemical interaction that would immobilize both chemicals. | (Di *et al.*, 2024) |
| Transcriptomics and proteomics | *Oryza sativa* | Rice seedlings were placed in 500g rice soil, watered with ¼ Hoagland solution. When seedlings were in 2-leaf stage, the soil was treated with or without 3 mg/kg Cd (added as CdCl_2_). Cd-treated plants were also treated with 0, 1 or 5 mg/kg Se (added as Na_2_SeO_3_) (4 treatments: CK, Cd, Cd + low Se and Cd + high Se) | Compared to control, Cd treatment significantly reduced fresh weight, plant height, chlorophyll- and carotenoid content. Levels of peroxide, malondialdehyde and protein carbonyl levels were increased by Cd but reduced by simultaneous low Se treatment. Genes involved in 1) cell wall metabolism, 2) energy production and 3) enzymatic and non-enzymatic antioxidants were increased in activity in low Se+Cd compared to Cd treatment. Genes involved in phytohormone signalling (IAA, ABA, JA) were also upregulated, suggesting that these hormones play a role in the Se response and perhaps the Se-mediated increase in Cd tolerance. | (Zhu *et al.*, 2024) |
| Metabolomics | *Medicago sativa* (alfalfa, cv. ﻿Tapioszelei 1) | Alfalfa seeds were grown in a greenhouse with soil from the university garden of Debrecen, Hungary. The soil was treated with 0 or 50 mg Se/kg as Na_2_SeO_3_, and plants were sampled after 39 days | The Se levels of control plants was quite low (0.1 mg Se/kg dry stem; 0.86 mg Se/kg dry leaf) but Se treatment significantly increased this (55.42 mg Se/kg dry stem; 145.6 mg Se/kg dry leaf). Metabolomic analysis indicated the presence of various selenosugar selenometabolites, along with dihydroxypropionyl-selenohomocysteine- and selenohomolanthionine derivatives. | (Domokos‑Szabolcsy et al., 2024) |
| Transcriptome | ﻿*Brassica oleracea* var. *Italica* (broccoli cv. BOP15-80) | Broccoli sprouts were cultured in a climate chamber, and 7d old plants were sprayed with 0, 40 or 400 𝞵M Na_2_SeO_3_ and harvested after 0, 1, 6 or 12 hours. | After 6 and 12 hours, both Se treatments significantly increased the sulforaphane content of broccoli sprouts. Genes involved in sulphurophane biosynthesis were identified from the transcriptomic data, and glutathione (GSH) was identified as having both 1) an important role as S donor in glucosinolate biosynthesis and 2) as a redox buffer in Se exposure. Three transcription factors (TFs) with direct binding capability to the promotor region of glucosinolate biosynthesis gene SUR1 were identified via yeast-one hybrids. The TFs GATA22, ERF12-like and MYB108 were identified to positively regulate SUR1 expression. | (Mao *et al.*, 2025) |
| Genotype screening and transcriptome | *Brassica rapa spp. pekinensis* (Chinese cabbage, 39 genotypes) | The 39 genotypes were cultivated in 8L 1x Hoagland’s nutrient solution. 30d old seedlings were then exposed to 0, 1, or 2.5 mg/L Na_2_SeO_3_ for 15 days. Transcriptomic analysis was performed on 0 and 5 mg/L Na_2_SeO_3_ treatments of two genotypes (P2 and P6) | The authors screened 39 genotypes of Chinese cabbage for high Se uptake and identified high Se genotype P2 and low-Se genotype P6. Subsequent transcriptomic analysis indicated that the high-Se variety P2 had a significantly higher expression of a nitrate transporter from the PTR family (*BrNP2.20*) compared to the low Se variety P6. Heterologous expression of *BrNP2.20* in yeast- and *Arabidopsis thaliana* increased sensitivity to 50 𝞵M Na_2_SeO_3_ and uptake of Na_2_SeO_3_ (60 𝞵M), respectively. These results suggest that this nitrate transporter has a moonlighting function as a selenite transporter. | (Hu *et al.*, 2025) |
| Transcriptome | *Cardamine violifolia* | 42d old seedlings were grown in 3.5L ¼ Hoagland solution with 0, 20 or 100 mg/L Na_2_SeO_3_ and 0, 50 or 100 mg/L CdCl_2_ for 15 days. Roots and shoots were used for transcriptomic analysis. | Compared to the control condition, the fresh weight of the 100 mg/L Cd-treated plants was slightly lower, and even lower when also treated with Se. However, simultaneous Se treatment did lower the level of H_2_O_2_, malondialdehyde (MDA) and increased activity of superoxide dismutase and ascorbate peroxidase. The main response of the transcriptome to Se and Cd exposure was found in 1) upregulating lignin biosynthetic processes, 2) downregulating photosynthesis and 3) altering root cell wall formation. Gene coexpression cluster analysis further indicated a strong correlation between selenocompound concentrations and expression of a gene cluster involved in 3) cell wall modification 4) root hair cell differentiation & development, 5) ABC transporter activity, 6) inorganic phosphate transporter activity and 7) lipid transporter activity. These results show a correlation between Se uptake, Cd exposure and development of root hair cells with active phosphate processes. Aside from the main focus on Cd and Se, high Se treatment significantly reduced iron levels in the shoot compared to control. | (Rao *et al.*, 2025) |
| Metabolome | *Arabidopsis thaliana* (thale cress, ecotype Col-0) | Seedlings were grown on mineral nutrient agar with 1% sucrose and 0.5% agarose. Seedlings were treated with 0, 2, 10, 20 or 50 𝞵M K_2_SeO_4_ | Increasing Se treatments significantly reduced the fresh weight of *A. thaliana* seedlings at every step. Expression of SULTR1;2 and APR3 was increased at the 2 𝞵M treatment but reduced again at the 10 𝞵M treatment level. The levels of non-sulfur secondary defence metabolites increased with Se treatment, while the levels of glucosinolates (sulfur defence metabolites) were reduced by Se treatment. Various amino acids (Gln, Thr, Ala, Pro, Glu, Asp, Lys, Ile, Val, Gly, Leu, Tyr, Phe) were also reduced in level in Se compared to control conditions, indicating a strong overall reduced amino acid level. In general, Se treatment of *A. thaliana* resulted in increased levels of carbon defence compounds, and reduced level of sulfuric- and nitrogen containing amino acids and glucosinolates.  [red. this could be interpreted in the light of the Se-induced S deficiency response] | (Li *et al.*, 2025) |

**References**

**Byrne SL, Durandeau K, Nagy I, Barth S**. **2010**. Identification of ABC transporters from Lolium perenne L. that are regulated by toxic levels of selenium. *Planta* **231**: 901–911.

**Cakr O, Turgut-Kara N, ArI S, Zhang B**. **2015**. De novo transcriptome assembly and comparative analysis elucidate complicated mechanism regulating *Astragalus chrysochlorus* response to selenium stimuli. *PLoS ONE* **10**: 1–16.

**Campos ACAL, van Dijk WFA, Ramakrishna P, Giles T, Korte P, Douglas A, Smith P, Salt DE**. **2021**. 1,135 ionomes reveal the global pattern of leaf and seed mineral nutrient and trace element diversity in *Arabidopsis thaliana*. *Plant Journal* **106**: 536–554.

**Cao D, Liu Y, Ma L, Jin X, Guo G, Tan R, Liu Z, Zheng L, Ye F, Liu W**. **2019**. Transcriptome analysis of differentially expressed genes involved in selenium accumulation in tea plant (Camellia sinensis). *PLoS ONE* **13**.

**Chao DY, Baraniecka P, Danku J, Koprivova A, Lahner B, Luo H, Yakubova E, Dilkes B, Kopriva S, Salt DE**. **2014**. Variation in sulfur and selenium accumulation is controlled by naturally occurring isoforms of the key sulfur assimilation enzyme ADENOSINE 5’-PHOSPHOSULFATE REDUCTASE2 across the arabidopsis species range. *Plant Physiology* **166**: 1593–1608.

**Cheng H, Li L, Dong J, Wang S, Wu S, Rao S, Li L, Cheng S, Li L**. **2023**. Transcriptome and physiological determination reveal the effects of selenite on the growth and selenium metabolism in mung bean sprouts. *Food Research International* **169**.

**Di X, Jing R, Qin X, Liang X, Wang L, Xu Y, Sun Y, Huang Q**. **2024**. The role and transcriptomic mechanism of cell wall in the mutual antagonized effects between selenium nanoparticles and cadmium in wheat. *Journal of Hazardous Materials* **472**: 134549.

**Dimkovikj A, Van Hoewyk D**. **2014**. Selenite activates the alternative oxidase pathway and alters primary metabolism in *Brassica napus* roots: Evidence of a mitochondrial stress response. *BMC Plant Biology* **14**: 1–15.

**Domokos‑Szabolcsy É, Soós Á, Kovács B, Kovács Z, Dernovics M**. **2024**. Water-soluble organic selenometabolites of alfalfa (Medicago sativa L.) green biomass-derived fractions. *Journal of Trace Elements in Medicine and Biology* **86**.

**Dou L, Tian Z, Zhao Q, Xu M, Zhu Y, Luo X, Qiao X, Ren R, Zhang X, Li H**. **2021**. Transcriptomic Characterization of the Effects of Selenium on Maize Seedling Growth. *Frontiers in Plant Science* **12**: 1–16.

**Fang J, Peng Y, Zheng L, He C, Peng S, Huang Y, Wang L, Liu H, Feng G**. **2024**. Chitosan-Se Engineered Nanomaterial Mitigates Salt Stress in Plants by Scavenging Reactive Oxygen Species. *Journal of Agricultural and Food Chemistry* **72**: 176–188.

**Feng X, Ma Q**. **2021**. Transcriptome and proteome profiling revealed molecular mechanism of selenium responses in bread wheat (Triticum aestivum L.). *BMC Plant Biology* **21**: 1–16.

**Freeman JL, Tamaoki M, Stushnoff C, Quinn CF, Cappa JJ, Devonshire J, Fakra SC, Marcus MA, McGrath SP, van Hoewyk D, *et al.*** **2010**. Molecular mechanisms of selenium tolerance and hyperaccumulation in *Stanleya pinnata*. *Plant Physiology* **153**: 1630–1652.

**Gong XP, Liang X, Wu CH, Guo Y, Zhao Y, Li SS, Li XH, Kong FM**. **2016**. Selenium effects and quantitative trait locus (QTL) mapping for mineral nutrient efficiency traits in wheat at the seedling stage. *Journal of Plant Nutrition* **39**: 1087–1102.

**Guo L, Liao Y, Deng S, Li J, Bu X, Zhu C, Zhang W, Cong X, Cheng S, Chen Q, *et al.*** **2024**. Genome-wide analysis of NAC transcription factors and exploration of candidate genes regulating selenium metabolism in Broussonetia papyrifera. *Planta* **260**: 1–15.

**Guo K, Yao Y, Yang M, Li Y, Wu B, Lin X**. **2020**. Transcriptome sequencing and analysis reveals the molecular response to selenium stimuli in Pueraria lobata (willd.) Ohwi. *PeerJ* **2020**.

**Van Hoewyk D, Garifullina GF, Ackley AR, Abdel-Ghany SE, Marcus MA, Fakra S, Ishiyama K, Inoue E, Pilon M, Takahashi H, *et al.*** **2005**. Overexpression of AtCpNifS enhances selenium tolerance and accumulation in *Arabidopsis* . *Plant Physiology* **139**: 1518–1528.

**Van Hoewyk D, Takahashi H, Inoue E, Hess A, Tamaoki M, Pilon-Smits EAH**. **2008**. Transcriptome analyses give insights into selenium-stress responses and selenium tolerance mechanisms in *Arabidopsis* . *Physiologia Plantarum* **132**: 236–253.

**Hu X, Chen Y, Xu W**. **2025**. Brassica rapa selenium transporter NPF2.20 (BrNPF2.20) accounts for Se-enrichment in Chinese cabbage. *Ecotoxicology and Environmental Safety* **289**.

**Hu H, Hu J, Wang Q, Xiang M, Zhang Y**. **2022**. Transcriptome analysis revealed accumulation-assimilation of selenium and physio-biochemical changes in alfalfa (Medicago sativa L.) leaves. *Journal of the Science of Food and Agriculture* **102**: 4577–4588.

**Hu HF, Hu JK, Wang QD, Xu ML, Fan HY**. **2024**. Integrated Agronomic Traits, Nutritional Quality, and Physiological Indices under Excessive Selenium: Anatomical Characters and miRNA-mRNA Expression Profiles Analyze Selenium-Tolerance in Alfalfa. *Russian Journal of Plant Physiology* **71**: 1–17.

**Huang C, Ying H, Yang X, Gao Y, Li T, Wu B, Ren M, Zhang Z, Ding J, Gao J, *et al.*** **2021**. The *Cardamine enshiensis* genome reveals whole genome duplication and insight into selenium hyperaccumulation and tolerance. *Cell Discovery* **7**.

**Hung CY, Holliday BM, Kaur H, Yadav R, Kittur FS, Xie J**. **2012**. Identification and characterization of selenate- and selenite-responsive genes in a Se-hyperaccumulator Astragalus racemosus. *Molecular Biology Reports* **39**: 7635–7646.

**Hussain S, Yin H, Peng S, Khan FA, Khan F, Sameeullah M, Hussain HA, Huang J, Cui K, Nie L**. **2016**. Comparative transcriptional profiling of primed and non-primed rice seedlings under submergence stress. *Frontiers in Plant Science* **7**: 1–16.

**Jia W, Liu Y, Shi L, Chu X**. **2020**. Investigation of Differentially Expressed Proteins Induced by Alteration of Natural Se Uptake with Ultrahigh-Performance Liquid Chromatography Quadrupole Orbitrap Uncovers the Potential Nutritional Value in Se-Enriched Green Tea. *Journal of Agricultural and Food Chemistry* **68**: 6316–6332.

**Jiang L, Lu Y, Zheng L, Li G, Chen L, Zhang M, Ni J, Liu Q, Zhang Y**. **2020**. The algal selenoproteomes. *BMC Genomics* **21**.

**Kök AB, Mungan MD, Doğanlar S, Frary A**. **2020**. Transcriptomic analysis of selenium accumulation in Puccinellia distans (Jacq.) Parl., a boron hyperaccumulator. *Chemosphere* **245**.

**Li H, Mori T, Moriyama R, Fujita M, Hatanaka G, Shiotsuka N, Hosomi R, Maruyama-Nakashita A**. **2025**. Non-Targeted Metabolome Analysis with Low-Dose Selenate-Treated Arabidopsis. *Plants* **14**: 322.

**Li W, Wang Y, Li J, Guo X, Song Q, Xu J**. **2024**. Selenite improves growth by modulating phytohormone pathways and reprogramming primary and secondary metabolism in tomato plants. *Plant Physiology and Biochemistry* **214**: 108930.

**Li L, Yu J, Yuan H, Zha S, Deng K, Xiao X, Luo Y, Cheng S, Cheng H**. **2019**. High-density kinetic analysis of the metabolomic and transcriptomic response of Ginkgo biloba flavonoids biosynthesis to selenium treatments. *Notulae Botanicae Horti Agrobotanici Cluj-Napoca* **47**: 792–803.

**Li D, Zhou C, Ma J, Wu Y, Kang L, An Q, Zhang J, Deng K, Li JQ, Pan C**. **2021**. Nanoselenium transformation and inhibition of cadmium accumulation by regulating the lignin biosynthetic pathway and plant hormone signal transduction in pepper plants. *Journal of Nanobiotechnology* **19**: 1–14.

**Liu C, Zhou G, Qin H, Guan Y, Wang T, Ni W, Xie H, Xing Y, Tian G, Lyu M, *et al.*** **2024**. Metabolomics combined with physiology and transcriptomics reveal key metabolic pathway responses in apple plants exposure to different selenium concentrations. *Journal of Hazardous Materials* **464**: 132953.

**Mao S, Wang J, Guo Z, Huang H, Wang S, Fei D, Liu J, Wu Q, Nie J, Wu Q, *et al.*** **2025**. Improving sulforaphane content in broccoli sprouts by applying Se: transcriptome profiling and coexpression network analysis provide insights into the mechanistic response. *Physiologia Plantarum* **177**: 1–16.

**Németh A, García Reyes JF, Kosáry J, Dernovics M**. **2013**. The relationship of selenium tolerance and speciation in Lecythidaceae species. *Metallomics* **5**: 1663–1673.

**Ouerdane L, Both EB, Xiang J, Yin H, Kang Y, Shao S, Kiszelák K, Jókai Z, Dernovics M**. **2020**. Water soluble selenometabolome of Cardamine violifolia. *Metallomics* **12**: 2032–2048.

**Poggi V, Del Vescovo V, Di Sanza C, Negri R, Hochkoeppler A**. **2008**. Selenite transiently represses transcription of photosynthesis-related genes in potato leaves. *Photosynthesis Research* **95**: 63–71.

**Qi WY, Li Q, Chen H, Liu J, Xing SF, Xu M, Yan Z, Song C, Wang SG**. **2021**. Selenium nanoparticles ameliorate Brassica napus L. cadmium toxicity by inhibiting the respiratory burst and scavenging reactive oxygen species. *Journal of Hazardous Materials* **417**: 125900.

**Qin Y, Cai Q, Ling Y, Chen X, Xu J, Huang G, Liang S, Yuan X, Yang XM, Lu D, *et al.*** **2023**. Arbuscular mycorrhizal fungi improve selenium uptake by modulating root transcriptome of rice (Oryza sativa L.). *Frontiers in Plant Science* **14**: 1–13.

**Qu L, Xu Z, Huang W, Han D, Dang B, Ma X, Liu Y, Xu J, Jia W**. **2024**. Selenium-molybdenum interactions reduce chromium toxicity in Nicotiana tabacum L. by promoting chromium chelation on the cell wall. *Journal of Hazardous Materials* **461**: 132641.

**Rao S, Cong X, Liu H, Hu Y, Yang W, Cheng H, Cheng S, Zhang Y**. **2022**. Revealing the Phenolic Acids in *Cardamine violifolia* Leaves by Transcriptome and Metabolome Analyses. *Metabolites* **12**.

**Rao S, Gong J, Liu H, Liu X, Cheng S, Cheng H, Cong X**. **2023**. Metabolome and Transcriptome Analyses Provide Insights into Glucosinolate Accumulation in the Novel Vegetable Crop *Cardamine violifolia*. *Agronomy* **13**: 2760.

**Rao S, Gou Y, Yu T, Cong X, Gui J, Zhu Z, Zhang W, Liao Y, Ye J, Cheng S, *et al.*** **2021a**. Effects of selenate on Se, flavonoid, and glucosinolate in broccoli florets by combined transcriptome and metabolome analyses. *Food Research International* **146**: 110463.

**Rao S, Yu T, Cong X, Lai X, Xiang J, Cao J, Liao X, Gou Y, Chao W, Xue H, *et al.*** **2021b**. Transcriptome, proteome, and metabolome reveal the mechanism of tolerance to selenate toxicity in *Cardamine violifolia*. *Journal of Hazardous Materials* **406**: 124283.

**Rao S, Yu T, Cong X, Xu F, Lai X, Zhang W, Liao Y, Cheng S**. **2020**. Integration analysis of PacBio SMRT- and Illumina RNA-seq reveals candidate genes and pathway involved in selenium metabolism in hyperaccumulator Cardamine violifolia. *BMC Plant Biology* **20**.

**Rao S, Zhu D, Liu H, Chen L, Liu X, Yang W, Cheng H, Cheng S, Cong X**. **2025**. Cadmium distinctly affects selenium accumulation in Cardamine violifolia roots and shoots. *Industrial Crops and Products* **229**: 120975.

**Ren H, Li X, Guo L, Wang L, Hao X, Zeng J**. **2022**. Integrative Transcriptome and Proteome Analysis Reveals the Absorption and Metabolism of Selenium in Tea Plants [Camellia sinensis (L.) O. Kuntze]. *Frontiers in Plant Science* **13**.

**Ribeiro DM, Silva Júnior DD, Cardoso FB, Martins AO, Silva WA, Nascimento VL, Araújo WL**. **2016**. Growth inhibition by selenium is associated with changes in primary metabolism and nutrient levels in *Arabidopsis thaliana*. *Plant Cell and Environment* **39**: 2235–2246.

**Roda FA, Marques I, Batista-Santos P, Esquível MG, Ndayiragije A, Lidon FC, Swamy BPM, Ramalho JC, Ribeiro-Barros AI**. **2020**. Rice Biofortification With Zinc and Selenium: A Transcriptomic Approach to Understand Mineral Accumulation in Flag Leaves. *Frontiers in Genetics* **11**: 1–14.

**Ruszczyńska A, Konopka A, Kurek E, Torres Elguera JC, Bulska E**. **2017**. Investigation of biotransformation of selenium in plants using spectrometric methods. *Spectrochimica Acta - Part B Atomic Spectroscopy* **130**: 7–16.

**Salin H, Fardeau V, Piccini E, Lelandais G, Tanty V, Lemoine S, Jacq C, Devaux F**. **2008**. Structure and properties of transcriptional networks driving selenite stress response in yeasts. *BMC Genomics* **9**: 1–14.

**Sams CE, Panthee DR, Charron CS, Kopsell DA, Yuan JS**. **2011**. Selenium regulates gene expression for glucosinolate and carotenoid biosynthesis in *Arabidopsis* . *Journal of the American Society for Horticultural Science* **136**: 23–34.

**Shang H, Li C, Cai Z, Hao Y, Cao Y, Jia W, Han L, White JC, Ma C, Xing B**. **2024**. Biosynthesized Selenium Nanoparticles as an Effective Tool to Combat Soil Metal Stresses in Rice (Oryza sativa L.). *ACS Nano*.

**Shi T, Wang Y, Li Y, Sui X, Dong CH**. **2024**. Generation of selenium-rich wheat mutants and exploration of responsive genes for selenium accumulation. *Plant Cell Reports* **43**: 1–14.

**Sun H, Wang X, Li H, Bi J, Yu J, Liu X, Zhou H, Rong Z**. **2020**. Selenium modulates cadmium-induced ultrastructural and metabolic changes in cucumber seedlings. *RSC Advances* **10**: 17892–17905.

**Tamaoki M, Freeman JL, Pilon-Smits EAH**. **2008**. Cooperative ethylene and jasmonic acid signaling regulates selenite resistance in *Arabidopsis* . *Plant Physiology* **146**: 1219–1230.

**Wang J, Cappa JJ, Harris JP, Edger PP, Zhou W, Pires JC, Adair M, Unruh SA, Simmons MP, Schiavon M, *et al.*** **2018**. Transcriptome-wide comparison of selenium hyperaccumulator and nonaccumulator *Stanleya* species provides new insight into key processes mediating the hyperaccumulation syndrome. *Plant Biotechnology Journal* **16**: 1582–1594.

**Wang Q, Hu J, Hu H, Li Y, Xiang M, Wang D**. **2022**. Integrated eco-physiological, biochemical, and molecular biological analyses of selenium fortification mechanism in alfalfa. *Planta* **256**: 1–18.

**Wang Q, Hu J, Lou T, Li Y, Shi Y, Hu H**. **2023a**. Integrated agronomic, physiological, microstructure, and whole-transcriptome analyses reveal the role of biomass accumulation and quality formation during Se biofortification in alfalfa. *Frontiers in Plant Science* **14**: 1–20.

**Wang X, Lu W, Zhao Z, Hao W, Du R, Li Z, Wang Z, Lv X, Wang J, Liang D, *et al.*** **2024a**. Abscisic acid promotes selenium absorption, metabolism and toxicity via stress-related phytohormones regulation in Cyphomandra betacea Sendt. (Solanum betaceum Cav.). *Journal of Hazardous Materials* **461**: 132642.

**Wang M, Mu C, Lin X, Ma W, Wu H, Si D, Ge C, Cheng C, Zhao L, Li H, *et al.*** **2024b**. Foliar Application of Nanoparticles Reduced Cadmium Content in Wheat (Triticum aestivum L.) Grains via Long-Distance “Leaf-Root-Microorganism” Regulation. *Environmental Science and Technology* **58**: 6900–6912.

**Wang M, Wang Y, Ge C, Wu H, Jing F, Wu S, Li H, Zhou D**. **2023b**. Foliar Selenium Nanoparticles Application Promotes the Growth of Maize (Zea mays L.) Seedlings by Regulating Carbon, Nitrogen and Oxidative Stress Metabolism. *Scientia Horticulturae* **311**: 111816.

**Wang LZ, Wu KY, Liu ZQ, Li ZF, Shen J, Wu ZH, Liu H, You LX, Yang G Di, Rensing C, *et al.*** **2023c**. Selenite reduced uptake/translocation of cadmium via regulation of assembles and interactions of pectins, hemicelluloses, lignins, callose and Casparian strips in rice roots. *Journal of Hazardous Materials* **448**: 130812.

**Wang F, Yang J, Hua Y, Wang K, Guo Y, Lu Y, Zhu S, Zhang P, Hu G**. **2023d**. Transcriptome and Metabolome Analysis of Selenium Treated Alfalfa Reveals Influence on Phenylpropanoid Biosynthesis to Enhance Growth. *Plants* **12**.

**Wu L, Liu T, Xu Y, Chen W, Liu B, Zhang L, Liu D, Zhang H, Zhang B**. **2019a**. Comparative transcriptome analysis of two selenium-accumulating genotypes of Aegilops tauschii Coss. in response to selenium. *BMC Genetics* **20**: 1–11.

**Wu KY, Wang LZ, Wu ZH, Liu ZQ, Li ZF, Shen J, Shi SJ, Liu H, Rensing C, Feng R**. **2024**. Selenite reduced cadmium uptake, interfered signal transduction of endogenous phytohormones, and stimulated secretion of tartaric acid based on a combined analysis of non-invasive micro-test technique, transcriptome and metabolome. *Plant Physiology and Biochemistry* **206**: 108107.

**Wu Q, Wu Q, Wang J, Mao S, Xu H, Liang M, Yuan Y, Liu M, Huang K**. **2019b**. Comparative transcriptome analyses of genes involved in sulforaphane metabolism at different treatment in Chinese kale using full-length transcriptome sequencing. *BMC Genomics* **20**: 1–13.

**Xia Q, Shui Y, Zhi H, Ali A, Yang Z, Gao Z**. **2023**. Exogeneous selenium enhances anthocyanin synthesis during grain development of colored-grain wheat. *Plant Physiology and Biochemistry* **200**: 107742.

**Xiong Y, Xiang X, Xiao C, Zhang N, Cheng H, Rao S, Cheng S, Li L**. **2023**. Illumina RNA and SMRT Sequencing Reveals the Mechanism of Uptake and Transformation of Selenium Nanoparticles in Soybean Seedlings. *Plants* **12**.

**Yang X, Liao X, Yu L, Rao S, Chen Q, Zhu Z, Cong X, Zhang W, Ye J, Cheng S, *et al.*** **2022**. Combined metabolome and transcriptome analysis reveal the mechanism of selenate influence on the growth and quality of cabbage (Brassica oleracea var. capitata L.). *Food Research International* **156**.

**Yu Y, Liu Z, Luo LY, Fu PN, Wang Q, Li HF**. **2019**. Selenium Uptake and Biotransformation in Brassica rapa Supplied with Selenite and Selenate: A Hydroponic Work with HPLC Speciation and RNA-Sequencing. *Journal of Agricultural and Food Chemistry*.

**Yu Y, Wang Q, Wan Y, Huang Q, Li H**. **2023**. Transcriptome analysis reveals different mechanisms of selenite and selenate regulation of cadmium translocation in Brassica rapa. *Journal of Hazardous Materials* **452**: 131218.

**Zeng R, Farooq MU, Wang L, Su Y, Zheng T, Ye X, Jia X, Zhu J**. **2019**. Study on differential protein expression in natural selenium-enriched and non-selenium-enriched rice based on itraq quantitative proteomics. *Biomolecules* **9**.

**Zhang LH, Abdel-Ghany SE, Freeman JL, Ackley AR, Schiavon M, Pilon-Smits EAH**. **2006a**. Investigation of selenium tolerance mechanisms in *Arabidopsis thaliana*. *Physiologia Plantarum* **128**: 212–223.

**Zhang L, Ackley AR, Pilon-Smits EAH**. **2007**. Variation in selenium tolerance and accumulation among 19 *Arabidopsis thaliana*accessions. *Journal of Plant Physiology* **164**: 327–336.

**Zhang L, Byrne PF, Pilon-Smits EAH**. **2006b**. Mapping quantitative trait loci associated with selenate tolerance in *Arabidopsis thaliana*. *New Phytologist* **170**: 33–42.

**Zhang F, Li X, Wu Q, Lu P, Kang Q, Zhao M, Wang A, Dong Q, Sun M, Yang Z, *et al.*** **2022**. Selenium Application Enhances the Accumulation of Flavones and Anthocyanins in Bread Wheat (Triticum aestivum L.) Grains. *Journal of Agricultural and Food Chemistry* **70**: 13431–13444.

**Zhang C, Xu B, Geng W, Shen Y, Xuan D, Lai Q, Shen C, Jin C, Yu C**. **2019a**. Comparative proteomic analysis of pepper (Capsicum annuum L.) seedlings under selenium stress. *PeerJ* **2019**.

**Zhang C, Xu B, Zhao CR, Sun J, Lai Q, Yu C**. **2019b**. Comparative de novo transcriptomics and untargeted metabolomic analyses elucidate complicated mechanisms regulating celery (Apium graveolens L.) responses to selenium stimuli. *PLoS ONE* **14**.

**Zhou C, Miao P, Dong Q, Li D, Pan C**. **2024**. Multiomics Explore the Detoxification Mechanism of Nanoselenium and Melatonin on Bensulfuron Methyl in Wheat Plants. *Journal of Agricultural and Food Chemistry* **72**: 3958–3972.

**Zhou Y, Tang Q, Wu M, Mou D, Liu H, Wang S, Zhang C, Ding L, Luo J**. **2018**. Comparative transcriptomics provides novel insights into the mechanisms of selenium tolerance in the hyperaccumulator plant *Cardamine hupingshanensis*. *Scientific Reports* **8**.

**Zhu S, Sun S, Zhao W, Yang X, Mao H, Sheng L, Chen Z**. **2024**. Utilizing transcriptomics and proteomics to unravel key genes and proteins of Oryza sativa seedlings mediated by selenium in response to cadmium stress. *BMC Plant Biology* **24**: 1–22.

**Ziegler G, Nelson R, Granada S, Krishnan HB, Gillman JD, Baxter I**. **2018**. Genomewide association study of ionomic traits on diverse soybean populations from germplasm collections. *Plant Direct* **2**: 1–14.

**Zou Y, Han C, Wang F, Tan Y, Yang S, Huang C, Xie S, Xiao X**. **2021**. Integrated Metabolome and Transcriptome Analysis Reveal Complex Molecular Mechanisms Underlying Selenium Response of Aloe vera L. *Journal of Plant Biology* **64**: 135–143.
